# Probing cell identity hierarchies by fate titration and collision during direct reprogramming

**DOI:** 10.15252/msb.202211129

**Published:** 2022-09-15

**Authors:** Bob A Hersbach, David S Fischer, Giacomo Masserdotti, Deeksha, Karolina Mojžišová, Thomas Waltzhöni, Diego Rodriguez‐Terrones, Matthias Heinig, Fabian J Theis, Magdalena Götz, Stefan H Stricker

**Affiliations:** ^1^ Institute of Stem Cell Research, Helmholtz Zentrum München German Research Center for Environmental Health Oberschleißheim Germany; ^2^ Division of Physiological Genomics, Biomedical Center Munich Ludwig‐Maximilians University Munich Germany; ^3^ Graduate School of Systemic Neurosciences, Biocenter Ludwig‐Maximilians University Munich Germany; ^4^ Institute of Computational Biology, Helmholtz Zentrum München German Research Center for Environmental Health Oberschleißheim Germany; ^5^ TUM School of Life Sciences Weihenstephan Technical University of Munich Freising Germany; ^6^ Department of Informatics Technical University of Munich Munich Germany; ^7^ Core Facility Genomics Helmholtz Zentrum München Oberschleißheim Germany; ^8^ German Excellence Cluster of Systems Neurology Biomedical Center Munich Munich Germany; ^9^ Present address: Research Institute of Molecular Pathology (IMP) Vienna Austria

**Keywords:** direct reprogramming, Collide‐seq, fate conversion, reprogramming, scRNA‐seq, Chromatin, Transcription & Genomics, Development

## Abstract

Despite the therapeutic promise of direct reprogramming, basic principles concerning fate erasure and the mechanisms to resolve cell identity conflicts remain unclear. To tackle these fundamental questions, we established a single‐cell protocol for the simultaneous analysis of multiple cell fate conversion events based on combinatorial and traceable reprogramming factor expression: Collide‐seq. Collide‐seq revealed the lack of a common mechanism through which fibroblast‐specific gene expression loss is initiated. Moreover, we found that the transcriptome of converting cells abruptly changes when a critical level of each reprogramming factor is attained, with higher or lower levels not contributing to major changes. By simultaneously inducing multiple competing reprogramming factors, we also found a deterministic system, in which titration of fates against each other yields dominant or colliding fates. By investigating one collision in detail, we show that reprogramming factors can disturb cell identity programs independent of their ability to bind their target genes. Taken together, Collide‐seq has shed light on several fundamental principles of fate conversion that may aid in improving current reprogramming paradigms.

## Graphical abstract

Collide‐seq, a single‐cell protocol for analysing how different reprogramming transcription factors achieve conversion and how cells resolve cell identity conflicts, reveals several fundamental principles of fate conversion.

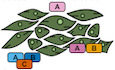

## Introduction

**Figure 1:**
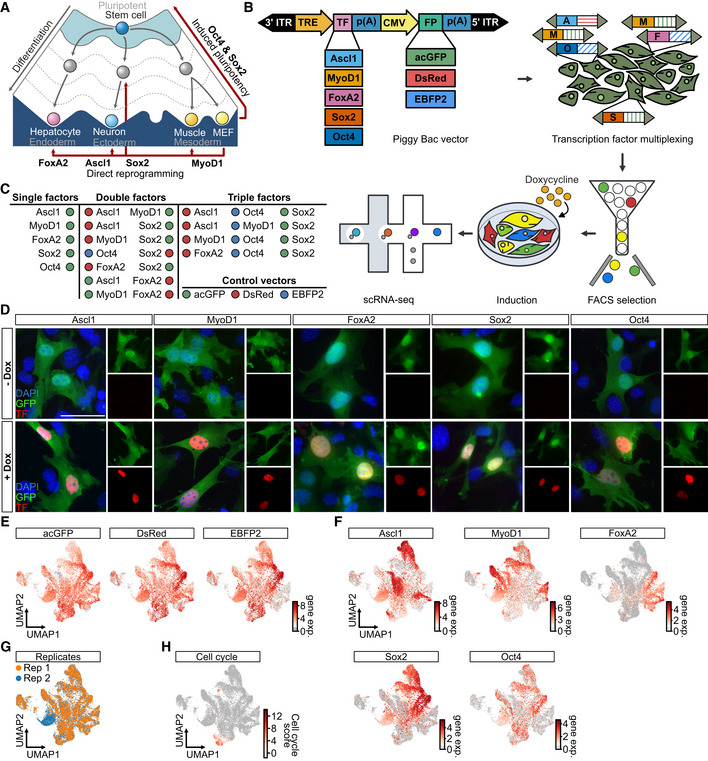
A multiplexed strategy for the comparison and collision of cell fate conversions ASchematic depiction of Waddington's landscape highlighting key transcription factors during development, induction of pluripotency, and direct reprogramming.BSchematic outline describing key aspects of Collide‐seq including (i) PiggyBac vectors used (top left), (ii) multiplexing of reprogramming factors (top right), (iii) selection of positive cells (bottom right) (iv) pooling of cells and transgene induction (bottom middle) and (v) scRNA‐seq (bottom left).COverview of all experimental conditions included in the Collide‐seq experiment including single, double, and triple transcription factor combinations.DRepresentative immunofluorescence images of MEFs 48 h after transgene induction stained for the indicated reprogramming factors. Scale bar represents 50 μm (*n* = 3 biological replicates).ELog fluorophore expression superimposed on Uniform Manifold Approximation and Projection (UMAP) embedding of the dataset. Shown are fluorophore expression levels (gene exp.) on a logarithmic (ln) scale.FLog transgene expression superimposed on a UMAP embedding of the dataset. Shown are transgene expression levels (gene exp.) on a logarithmic (ln) scale.GUMAP embedding of scRNA‐seq dataset colored by technical replicate. Orange: Replicate 1, Blue: Replicate 2.HCell‐cycle score superimposed on UMAP embedding of the dataset. See Unsupervised analysis of single‐cell RNA‐seq section in Materials and Methods for further details. Source data are available online for this figure.

Mammalian development, through a complex set of intrinsic and extrinsic signals, leads to a plethora of functionally distinct cell types characterized by gene regulatory networks that are stable under physiological conditions (Vickaryous & Hall, 2006; Enver *et al*, 2009; Graf & Enver, 2009). Surprisingly, forced expression of key fate‐determining transcription factors is not only sufficient to perturb these networks, but also to successfully convert one cell type into the other, a process referred to as transdifferentiation or direct reprogramming (Fig 1A; Iwafuchi‐Doi & Zaret, 2014; Morris, 2016). Direct reprogramming was first described when fibroblasts were transdifferentiated into contracting muscle cells (Davis *et al*, 1987; Weintraub *et al*, 1989) and was later extended to other paradigms, including the direct conversion of ectoderm‐derived glia into neurons (Heins *et al*, 2002). Since then, reprogramming has been established for many cell types and even across germ layers, such as the conversion of fibroblasts into neurons, hepatocytes, and most strikingly, induced pluripotent stem cells (Takahashi & Yamanaka, 2006; Vierbuchen *et al*, 2010; Huang *et al*, 2011).

The current wealth of different reprogramming paradigms suggests that virtually any cell might be converted into any other cell type. However, despite the variety of available reprogramming paradigms and efforts to investigate the underlying molecular mechanisms (Buganim *et al*, 2012; Chronis *et al*, 2017; Fu *et al*, 2018; Dall'Agnese *et al*, 2019; Velychko *et al*, 2019; Cates *et al*, 2021; Kempf *et al*, 2021; Yagi *et al*, 2021) several fundamental questions remain unanswered. For example, it is currently unknown whether different fate conversions share similar principles, i.e., whether general mechanisms to achieve the loss of starter cell identity exist. Similarly, it is also unclear to which extent the expression levels of reprogramming factors influence both loss and gain of cell identity during reprogramming. For instance, do transcriptomic changes scale with reprogramming factor expression levels and, as a result, fate conversion occurs in a gradual manner, or do critical thresholds exist instead? This distinction would be particularly relevant for members of larger transcription factor families, such as basic helix loop–helix (bHLH) proteins, which share low‐affinity sites and are consequently subject to binding site competition (Long *et al*, 2016). Finally, another widely overlooked aspect of cell fate conversion is that it always entails a cell identity conflict, i.e., the existing cell identity gets challenged by a reprogramming transcription factor attempting to induce a conflicting program. We know, however, very little about the molecular basis of cell identity conflicts, particularly on which molecular level they collide and how cells resolve such conflicts. For example, can mismatched transcriptional programs be present simultaneously for a significant amount of time or does competition for co‐factors and/or DNA binding interfere with the full implementation of programs? Major obstacles to address these questions from publicly available data are the lack of systematic side‐by‐side experiments and the widespread use of optimized medium conditions during different reprogramming protocols, imposing a strong bias on gene expression (Chen *et al*, 2014; Kim *et al*, 2015; Kleijkers *et al*, 2015; Ledur *et al*, 2017). Hence, no direct comparisons of the impact various fate‐instructing reprogramming factors have on starter cell identity have been performed to date. Similarly, addressing whether expression levels of reprogramming factors are significant for converting the cellular transcriptome has also not been systematically evaluated so far. Finally, although combinations of compatible factors have been used to obtain specific cell types of interest (Takahashi & Yamanaka, 2006; Vierbuchen *et al*, 2010; Huang *et al*, 2011; Sekiya & Suzuki, 2011) it is unclear how cells respond to conflicting fates being imposed on the cell at the same time.

To explore cell identity hierarchies and mechanisms of fate collision, we developed Collide‐seq, implementing novel experimental approaches and analysis tools to quantify reprogramming factor levels and investigate, on a single cell level, how fibroblasts convert into lineages of different germ layers and how cell identity conflicts are resolved.

## Results

### A multiplexed strategy for the comparison and collision of cell fate conversions

In order to gain a mechanistic insight into cell fate conversion, we chose experimental conditions that allowed a systematic comparison of the impact different reprogramming factors have on the transcriptome. This included the choice of a versatile starter cell, mouse embryonic fibroblasts (MEFs), which have been extensively used in numerous reprogramming paradigms (Davis *et al*, 1987; Takahashi & Yamanaka, 2006; Vierbuchen *et al*, 2010; Caiazzo *et al*, 2011; Huang *et al*, 2011; Li *et al*, 2011; Nemajerova *et al*, 2012; Ring *et al*, 2012; Chanda *et al*, 2014; Colasante *et al*, 2015; Lim *et al*, 2016; Guo *et al*, 2017; Xiao *et al*, 2018). Furthermore, we focused our analysis on an early time point (72 h) to study the primary events of cell fate conversion and opted for a neutral cell culture medium, devoid of strong signaling molecules that would favor certain lineages over others and thereby alter the transcriptome independently of reprogramming factor function. Next, we selected a diverse panel of reprogramming factors: Ascl1, MyoD1, FoxA2, Sox2, and Pou5f1 (hereafter referred to as Oct4), which represent well‐known master regulators of different cellular identities belonging to distinct germ layers (Fig 1A and B; i.e., neuronal, myogenic, hepatogenic, multipotent and pluripotent, Table 1; Davis *et al*, 1987; Takahashi & Yamanaka, 2006; Grinnell *et al*, 2007; Vierbuchen *et al*, 2010; Chen *et al*, 2011; Huang *et al*, 2011; Li *et al*, 2011; Tsai *et al*, 2011; Wu *et al*, 2011; Ring *et al*, 2012; Chanda *et al*, 2014; Lim *et al*, 2016; Nakamori *et al*, 2017). Although the somatic reprogramming factors (Ascl1, MyoD1, FoxA2, and Sox2) are often used in combination with complementary factors, they are considered key drivers of conversion within their lineage and, under defined media conditions, are capable of achieving reprogramming on their own (Davis *et al*, 1987; Ring *et al*, 2012; Chanda *et al*, 2014; Guo *et al*, 2017). Additionally, the inclusion of both Sox2 and Oct4 allowed us to further investigate whether pluripotency factors act as general enablers of cell fate changes as recently suggested (Deleidi *et al*, 2011; Kim *et al*, 2011; Peskova *et al*, 2019; Sharma *et al*, 2019).

**Table 1:** Overview of different reprogramming factor and the characteristics of their target cells.

TF	Target cell	Germ layer	Potency	Cell‐cycle status
Ascl1	Neuron	Ectoderm	Terminally differentiated	Postmitotic
MyoD1	Skeletal muscle	Mesoderm	Terminally differentiated	Postmitotic
FoxA2	Hepatocyte	Endoderm	Terminally differentiated	Mitotic
Sox2	Neural stem cells	Ectoderm	Multipotent	Mitotic
Oct4	Pluripotent stem cell	Pregerm layer	Pluripotent	Highly proliferative

**Figure EV1:**
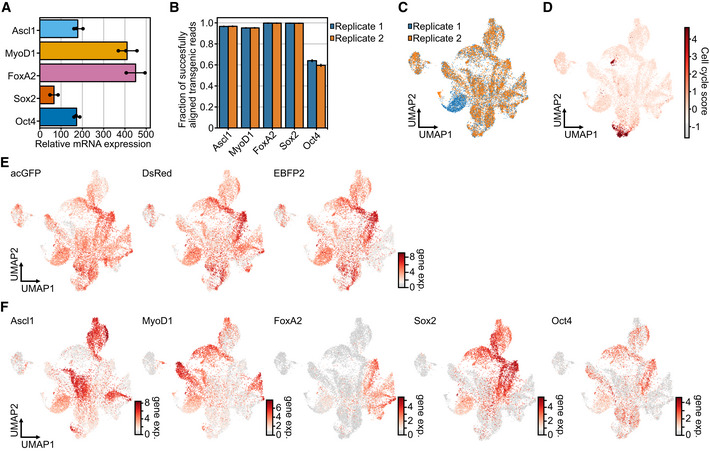
Analysis of transgene induction and detection AQuantification of transgene induction using qRT–PCR. Shown is the mean fold change of doxycycline‐induced samples over noninduced samples for the indicated transcription factors after 48 h (*n* = 2/3). Error bars represent 95% confidence interval. Primers used for qPCR are listed in Appendix Table S2.BFraction of UMIs mapped to transgenic allele within cells expressing a single reprogramming factor. Reads for transgenic transcripts were distinguished from endogenous transcripts using a custom annotation based on single nucleotide polymorphisms (See Generation of a modified gene annotation section in Materials and Methods for further details). Shown are 15,768 cells per barplot with the 95% confidence interval as error bars.CUniform Manifold Approximation and Projection (UMAP) embedding of scRNA‐seq data colored by technical replicate (Replicate 1: blue, Replicate 2: orange) after ambient RNA correction.DCell‐cycle score superimposed on UMAP embedding of ambient corrected data (See Unsupervised analysis of single‐cell RNA‐seq data in Materials and Methods).ELog fluorophore expression superimposed on a UMAP embedding after ambient RNA correction. Shown are fluorophore expression levels (gene exp.) on a logarithmic (ln) scale.FLog transgene expression superimposed on a UMAP embedding after ambient RNA correction. Shown are transgene expression (gene exp.) levels on a logarithmic (ln) scale. Source data are available online for this figure.

We cloned these factors into a PiggyBac vector, placing their expression under the control of a doxycycline‐dependent promoter (TRE), and added constitutively expressed fluorescent reporters (acGFP, EBFP2, DsRed; Fig 1B and C). A major benefit of using PiggyBacs, is the ability to obtain polyadenylated transcripts for the reporter and the transgene, facilitating the detection and quantification of transgene levels by scRNA‐seq platforms. Furthermore, between 1 and 10 PiggyBac vectors typically integrate, leading to a broad range of transcript expression between different cells (Yusa *et al*, 2009). By using a doxycycline‐inducible promoter, we further ensured synchronous expression and a defined experimental starting point. Importantly, no protein expression was detected in the absence of doxycycline, but a strong nuclear signal was seen 48 h after induction, indicating tight chemical control of reprogramming factor expression (Figs 1D and EV1A). Constitutive fluorescent reporter expression cassettes, on the other hand, enabled the enrichment and pooling of cells prior to transgene induction, i.e., initiation of cell fate conversion and scRNA‐seq.

Next, we nucleofected MEFs with constructs for the individual factors (Ascl1, MyoD1, FoxA2, Sox2, and Oct4) and their combinations. This yielded a total number of 17 different conditions, including cells expressing only the fluorescent reporters as a negative control (Fig 1C). After collecting an equal number of cells per condition and subsequently pooling them, cells were plated in a single well and transgene expression was induced for 72 h prior to single‐cell RNA sequencing (scRNA‐seq) using the 10x Genomics Chromium platform. In total, this returned ∼17,000 cells (Replicate 1: 8,709 cells, Replicate 2: 8,219 cells) for downstream analysis after applying strict quality control criteria (see Materials and Methods). As expected, nearly all cells expressed fluorescent reporters and at least one reprogramming factor (Fig 1E and F). Moreover, the expression of each transcription factor was localized to distinct parts of the *Uniform Manifold Approximation and Projection* (UMAP; preprint: McInnes *et al*, 2018) embedding, indicating that reprogramming factor expression and transcriptomic effects were well correlated (Fig 1E and F). These attained cell states were overall also highly similar between both technical replicates, demonstrating that our method yields robust results (Fig 1G). Furthermore, reprogramming factor transcripts were confirmed to be mostly derived from PiggyBacs, as a custom single nucleotide polymorphism (SNP) based alignment strategy attributed the majority of reprogramming factor UMIs to the transgenic allele (Fig EV1B, see Materials and Methods). This indicated that self‐reinforcement plays a minor role at the initial stage of cell conversion. In addition, the observed cellular heterogeneity was also not a technical artifact originating from ambient effects, as shown by applying ambient RNA correction to this data (Young & Behjati, 2020; Fig EV1C–F, see Materials and Methods). Finally, cell‐cycle analysis indicated that most cells expressing a reprogramming factor had exited the cell cycle after 72 h, irrespective of whether the expressed reprogramming factor ultimately steers towards a postmitotic cell fate (Ascl1 and MyoD1) or not (FoxA2, Oct4, and Sox2; Fig 1H). Altogether, our experimental paradigm, which we will hereafter refer to as Collide‐seq, allowed reading out the multiplexed reprogramming factor expression, which correlated well with induced transcriptomic effects, thus making it a suitable system to explore the general principles of early reprogramming events.

### Demultiplexing combinatorial reprogramming factor expression reveals discrete induction of distinct transcriptomic states

**Figure 2:**
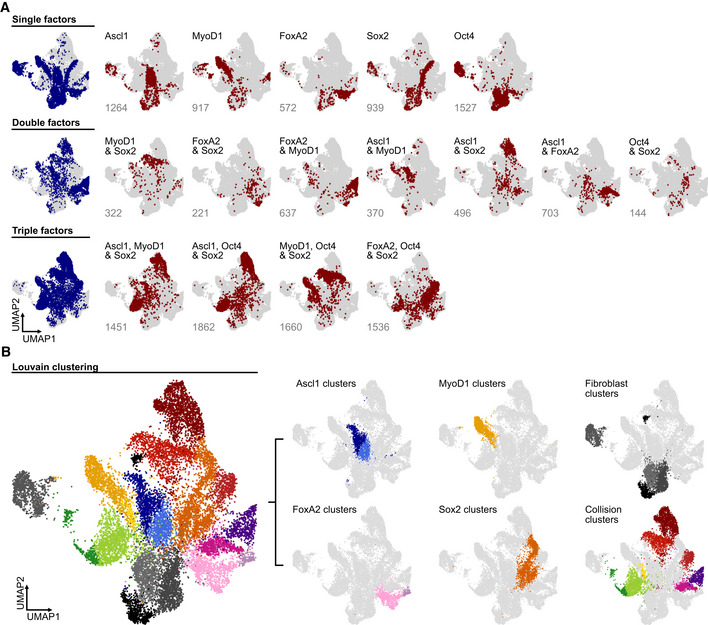
Demultiplexing of combinatorial reprogramming factor expression reveals discrete induction of distinct lineages AVisualization of condition assignment outcome (see Computational demultiplexing section in Materials and Methods for further details) superimposed on a Uniform Manifold Approximation and Projection (UMAP) embedding of the dataset. Depicted are the total population of cells carrying one, two, or three factors (left panels, blue shades) and the individual conditions (right panels, red shades) for the indicated conditions. For individual conditions, numbers indicate the total number of cells per condition.BLouvain clustering of the entire dataset (left panel) separated into clusters corresponding to the expression of the individual transcription factors, the ground fibroblast state, and the collision of reprogramming factors (right panels). Clusters are labeled according to the predominant transcription factor or cell state.

As we pooled cells from the different conditions to reduce batch effects and allow a systematic analysis of each condition, we next aimed to demultiplex the dataset into its individual conditions. Previous approaches for single‐cell knock‐out state inference (Dixit *et al*, 2016) are liable to yield false discoveries that derive from the knock‐out state being modeled as a mixture model of gene expression. Hence, we devised a novel computational approach to assign cells to their maximum *a posteriori* fit condition, leveraging the unique signature of transgene and fluorophore expression by cells from each condition (Fig 2A, Appendix Fig S1A, see Materials and Methods). Importantly, we did not use any additional transcriptomic information to assign conditions, as this would possibly bias assignment. This model was regularized by the set of conditions defined in the experimental setup (Fig 1C) and the inferred conditions mapped to distinct parts of the cell state space (Fig 2A). Accordingly, Louvain clustering stratified these states into distinct clusters corresponding to Ascl1 (blue shades), MyoD1 (yellow/orange shades), FoxA2 (pink/purple shades) and Sox2 (vermillion/red shades) induced transcriptional changes (Fig 2B). The Oct4‐positive cells, however, did not separate into a distinct cluster but were rather intermingled with the original fibroblast population (Fig 2A and B, fibroblasts, gray shades). Furthermore, the fibroblast population showed four clusters, three of which were close to each other, potentially representing different cell‐cycle states (Fig 1H), while one was more separated and possibly contained damaged cells given the relatively low gene and read count numbers (Fig 2B, left most fibroblast cluster, Appendix Fig S1B). Most importantly, several clusters contained cells expressing more than one factor, indicating that collision between factors yields distinct cell states at 72 h postinduction (Fig 2A and B). These collision states did not show increased fractions of mitochondrial reads, a hallmark of cellular stress (Ilicic *et al*, 2016; Luecken & Theis, 2019), implying that these states are viable (Appendix Fig S1C). Taken together, these results revealed distinct transcriptomic consequences induced by each factor and their combinations, which are well detected and tolerated for at least 72 h after transgene induction, thereby validating these single factors as sufficient to induce distinct transcriptomic changes.

### Comparing principles of fate conversion between single reprogramming factors

**Figure 3:**
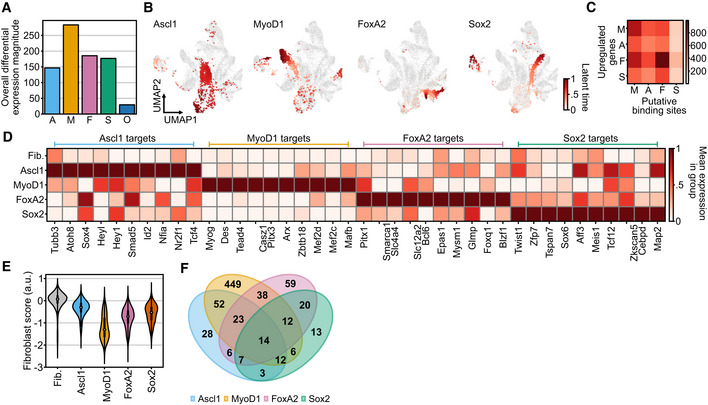
Comparing principles of fate conversion between distinct lineages ABar plot of transgene‐wise coefficient vector magnitude as a measure of induced transcriptomic changes by individual reprogramming transcription factors (see Differential expression analysis section in Materials and Methods for further details, A = Ascl1, M = MyoD1, F = FoxA2, S = Sox2, O = Oct4).BLatent pseudotime (lineage progression) superimposed on a Uniform Manifold Approximation and Projection (UMAP) of the dataset computed with scVelo (Bergen *et al*, 2020; see RNA velocity and CellRank analysis section in Materials and Methods for further details).CHeatmap showing the intersection of upregulated genes in individual factor conditions (*y*‐axis) vs. putative binding sites derived from a publicly available ChIP dataset for MyoD1 (M), Ascl1 (A), FoxA2 (F), and Sox2 (S) (*x*‐axis; Oki *et al*, 2018).DMatrixplot showing the relative mean expression of key target genes related to the different lineages for the indicated single factor conditions and control vector expressing fibroblasts (Fib.).EFibroblast score (see Unsupervised analysis of single‐cell RNA‐seq data in Materials and Methods and Appendix Table S1 for further details) for single factor conditions compared with fibroblasts expressing only control vectors (Fib. = Fibroblasts). The number of data points per violin plot is the number of cells per matched condition shown in Fig 2A. For each violin, the center dot represents the median, the centerline defines the range and the solid box marks the interquartile range (IQR).FVenn diagram of downregulated gene overlap between single factor conditions. Downregulated genes were determined by performing differential expression between each condition and the control vector carrying fibroblasts (See Differential expression section in Materials and Methods for further details).

Having established and verified the validity of Collide‐seq, we next aimed to identify the common and unique properties of different fate conversions. We first focused on transcriptomic changes in cells expressing a single reprogramming factor. To this end, we fit a generalized linear model to the cellular expression vectors as a function of transgene expression and computed the overall differential expression magnitude for each factor across all genes (see Materials and Methods). This can be interpreted as the deviation from the original fibroblast identity induced by each factor and revealed a clear hierarchy: MyoD1 had the highest magnitude while Ascl1, FoxA2, and Sox2 had smaller but comparably strong effects (Fig 3A, see Materials and Methods). Oct4 failed to significantly perturb cell identity on its own, hence, we focused on the four somatic factors for this part of the analysis.

A main advantage of Collide‐seq is that, due to the multiplexing of conditions in a single biological replicate, most confounding factors (i.e., culture conditions, culture media differences, etc.) are excluded. Hence, we could separate this general differential expression magnitude (Fig 3A) into two parts: (i) gene activation events related to fate acquisition and (ii) transcriptional repression associated with starter cell identity erasure. For fate acquisition, demultiplexing and Louvain clustering revealed that all four factors show a wide range of reprogramming factor expression with strong cell‐to‐cell variation (Appendix Fig S2A). Nevertheless, when individually expressed, each of them induced a distinct transcriptomic state (Fig 2B) and their lineage changes could also be resolved through an individual RNA velocity model (Bergen *et al*, 2020; Fig 3B). Moreover, using published ChIP‐seq data (Oki *et al*, 2018), a substantial overlap between putative binding sites and genes induced in our dataset, particularly for Ascl1, MyoD1 and FoxA2 was found (Fig 3C). This indicates that, at least for fate acquisition, a large fraction of transcriptional changes occurring at these early experimental time points might be direct consequences of reprogramming factor expression (Fig 3C). Furthermore, and despite the use of neutral culture conditions, each factor induced a distinct transcriptomic signature, characterized by unique target gene expression (Fig 3D) and gene ontology (GO) enrichments fitting to the corresponding lineages (Appendix Fig S2B–E). Overall, these results confirmed the competence of Ascl1, MyoD1, FoxA2, and Sox2 to trigger transcriptomic changes of cell fate conversion towards their respective lineages.

In addition to the differential expression magnitude (Fig 3A) that indicated each factor drives the transcriptomic state away from the original fibroblast identity, we also aimed to specifically measure the loss of fibroblast identity induced by each factor. To this end, we first devised a fibroblast score based on a literature‐derived list of fibroblast marker genes (Appendix Table S1, see Materials and Methods). Applying this score to the single factor data showed that all factors cause fibroblast identity loss after 72 h of reprogramming factor induction, though MyoD1 was most potent in this regard (Fig 3E). A more global analysis validated this observation, as genes encoding for extracellular matrix and cell periphery proteins, core parts of the fibroblast proteome, were significantly enriched among the genes downregulated by all four factors (Appendix Figs S3A–D and S4A–D, see Materials and Methods). Interestingly, however, these genes were rarely shared among all factors, suggesting that there is no common gene set or mechanism through which fibroblast identity is erased (Fig 3F). Notably, we did find substantial pairwise overlap in downregulated gene sets (i.e., 23% Sox2‐FoxA2, 21% FoxA2‐MyoD1, 35% Ascl1‐MyoD1), suggesting similar strategies for some factor pairs, while others possibly follow different repressive schemes. In line with having the biggest impact on the fibroblast transcriptome, MyoD1 was most potent in repressing the fibroblast signature, reflected by the fact that most of its downregulated genes were not shared with the other factors (Fig 3F). Overall, this analysis revealed that few similarities exist between different reprogramming factors when it comes to achieving fibroblast identity repression.

### Collision of multiple reprogramming factors reveals mostly antagonistic effects

**Figure 4:**
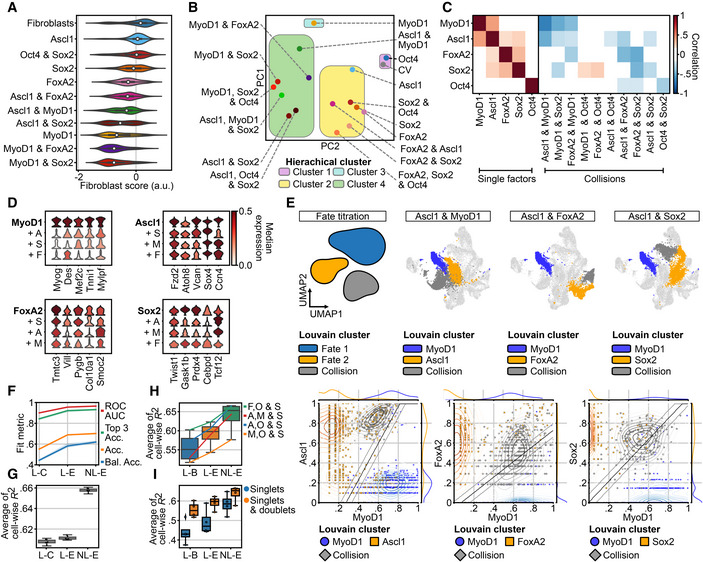
Factor collision reveals antagonistic effects of a deterministic system AFibroblast score (see Unsupervised analysis of single‐cell RNA‐seq data section in Materials and Methods for further details) for single and double factor conditions in comparison to control vector carrying fibroblasts. The number of data points per violin plot is the number of cells per matched condition shown in Fig 2A. For each violin, the center dot represents the median, the centerline defines the range and the solid box marks the interquartile range (IQR).BPCA scatter plot of first (PC1, vertical) and second (PC2, horizontal) loading of the different conditions after collapsing into pseudobulk samples. Colored rectangles correspond to clusters defined in Fig EV2B. CV: Control vector expressing fibroblasts.CCorrelation matrix of linear model effects for single (left panel) and double factor (right panel) collisions. Color defines direction of correlation of gene‐wise coefficient vectors (negative = blue, positive = red) and color tone depicts size of correlation. See Differential expression analysis section in Materials and Methods for further details.DStacked violin plots showing the standardized median expression of several lineage marker genes for single factor conditions (bold) and upon collision with the indicated factors (A = Ascl1, M = MyoD1, F = FoxA2, S = Sox2).EConceptual summary of fate titration analysis (upper left panel) and data‐derived examples for the indicated combinations of factors. Shown are the Louvain cluster assignments for the indicated collisions (upper panels) and the decision boundaries for indicated intermediate fates according to transcription factor levels (lower panels). Transcription factor levels shown on the *x*‐ and *y*‐axis are log‐normalized expression values scaled into the dynamic range of the single‐positive condition. Cells are colored according to their Louvain cluster identity. See Fate titration section in Materials and Methods for further details.F, GPredictive performance for a linear model of the categorical condition variable (L‐C: Linear condition.), a linear model of the log of the transgene expression (L‐E: Linear expression), and a nonlinear model of the log of the transgene expression (NL‐E: Nonlinear expression) in randomly held‐out test cells (*n* = 1,184 cells). For the task of Louvain cluster assignment prediction (F), shown are the area under the receiver–operator characteristic curve (ROC AUC), top 3 accuracy (Top 3 Acc.), Accuracy (Acc.), and class‐balanced accuracy (Bal. Acc.). For the task of prediction of log‐normalized expression values of highly variable genes (G), shown is the cell‐wise R2 (G). See Supervised modeling section in Materials and Methods for further details. For each box in G, the centerline defines the median, the height of the box is given by the interquartile range (IQR), the whiskers are given by 1.5 * IQR, and the outliers are given as points beyond the minimum or maximum whisker.H, IPredictive performance on held‐out reprogramming conditions for models and metrics as described in (H), with the exception of the baseline model, which is a linear model of binary transgene presence (L‐B: Linear binary). The hold‐out task in (H) is a particular triple‐positive condition as indicated in the legend (*N* = 4). The hold‐out task in (I) is the set of all triple‐positive conditions and models are either trained on single‐positive conditions only or on single‐ and double‐positive conditions (*N* = 4). See Supervised modeling section in Materials and Methods for further details. For each box, the centerline defines the median, the height of the box is given by the interquartile range (IQR), the whiskers are given by 1.5 * IQR, and the outliers are given as points beyond the minimum or maximum whisker.

**Figure EV2:**
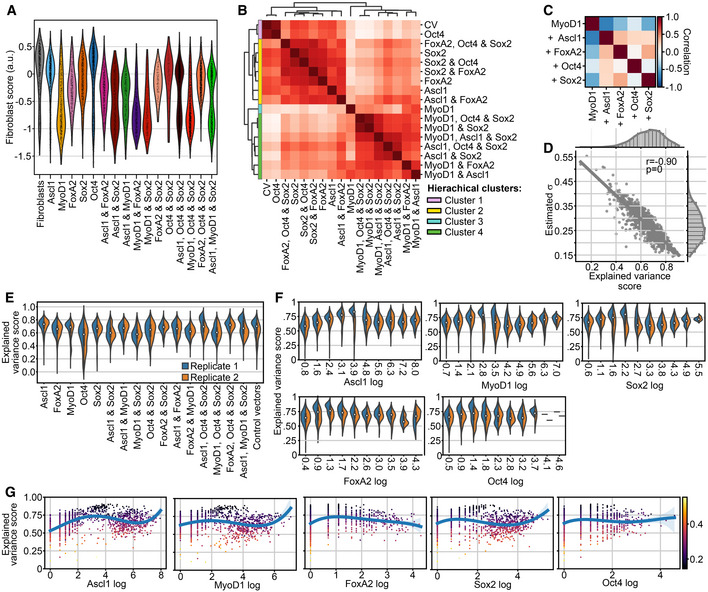
Transcriptomic effects of factor collision and general determinism in Collide‐seq data AFibroblast score (see Unsupervised analysis of single‐cell RNA‐seq data section in Materials and Methods and Appendix Table S1 for further details) for indicated conditions. The number of data points per violin plot is the number of cells per matched condition shown in Fig 2A.BSimilarity of pseudobulk (mean expression per condition) principal components per condition clustered hierarchically. Colors of clusters correspond to those used in Fig 4B.CCorrelation matrix of linear model effects for correlation between individual factors and collision state (see Differential expression analysis section in Materials and Methods for further details). Color defines direction of correlation (negative = blue, positive = red), and color tone depicts size of correlation (darker = higher).DCorrelation of predictive accuracy, measured as explained variance, on held‐out test data with the predicted uncertainty of the model, measured as the mean of the predicted standard deviation by gene.EExplained variance of model prediction by condition and replicate. For each violin, the center dot represents the median, the centerline defines the range and the solid box marks the interquartile range (IQR).F, GExplained variance of cells binned by individual transgene expression (F) or individually with trend fit (G). In (G), the color indicates the predicted uncertainty measured as the mean standard deviation over output genes. For each violin in F, the center dot represents the median, the centerline defines the range and the solid box marks the interquartile range (IQR).

Building on the single factor data for the removal of starter cell identity, we next asked whether multiple factors synergize or antagonize in fate erasure. Using the same fibroblast score as above, factor collisions were shown to have intricate effects on switching off fibroblast identity (Fig 4A). For instance, the combination of Ascl1 and Sox2 repressed fibroblast identity more efficiently than either Ascl1 or Sox2 alone (Fig 4A). By contrast, cells expressing both Ascl1 and MyoD1 maintained a much higher fibroblast score than those expressing MyoD1 alone, while neither Sox2 nor FoxA2 had such a negative impact on MyoD1 (Fig 4A). Furthermore, the addition of Sox2 and Oct4 did not improve identity loss for any of the other factors, suggesting that these pluripotency genes do not act as general enablers by facilitating starter cell identity removal, as has been suggested before (Deleidi *et al*, 2011; Kim *et al*, 2011; Peskova *et al*, 2019; Sharma *et al*, 2019). This reinforces the concept that different molecular mechanisms for cell identity removal exist and demonstrates that these mechanisms can act synergistically in some instances, yet antagonistic or neutral outcomes are more common (Figs 4A and EV2A).

We then analyzed how transcriptomic changes caused by one reprogramming factor are perturbed by other factors. For this, we first collapsed the cells within each condition to their mean, forming pseudobulk samples, and performed hierarchical clustering (Fig EV2B). Next, we performed a principal component analysis of these pseudobulk samples and overlaid it with the hierarchical cluster labels (Figs 4B and EV2B, see Materials and Methods). This revealed that no factor was clearly dominant over all other factors, although in some of the combinations one reprogramming factor had significant dominance over the other (Fig 4B). Strikingly, MyoD1 only expressing cells were significantly different from all other conditions (Figs 4B, and EV2B and C). This unique transcriptional identity of the MyoD1 pseudobulk sample supports our findings on its differential expression magnitude (Fig 3A) and its striking potency in erasing fibroblast identity (Fig 3E), indicating MyoD1 is the putative most potent reprogramming factor in our setting. Nevertheless, the striking dissimilarity between the MyoD1‐only condition and double/triple factor combinations containing MyoD1 indicated that the other factors can perturb the myogenic trajectory significantly (Fig 4B).

Following up on this, we next quantified the degree of synergism and antagonism in collisions by analyzing pairwise interactions between factors in a linear model of cellular expression vectors. The interaction coefficients in this model can be interpreted as positive (synergistic) or negative (antagonistic) effects that are evoked by the collision on the expression vector of the single factor conditions. We correlated the coefficient vectors of each term in the linear model to understand their relative effect on gene expression (Fig 4C). For the individual reprogramming factors, mostly positive correlation coefficients were found, indicating a sign change in a similar direction (Fig 4C), likely caused by similarities in fibroblast identity removal. By contrast, the effects observed upon the co‐expression of two or more reprogramming factors produced a generally negative or neutral correlation, thus indicating an antagonistic or nonsubstantial change (Fig 4C). This demonstrated that fate collisions are often detrimental to the individual fate, as conversions are partially undone by the collision. Notably, we again found no indication that pluripotency factors Oct4 and Sox2 further enhance cellular conversion in any of the combinations at this early time point. In terms of strength, the negative correlation for the collision between MyoD1 and Ascl1 was strongest. As single factors, their effects showed a high correlation coefficient (Fig 4C, intersection of MyoD1 and Ascl1 and vice versa) confirming commonalities in their induced programs, which have been reported previously (Lee *et al*, 2020). However, their co‐expression did not result in synergism, as might be expected, but rather displayed a very strong antagonistic effect (Fig 4C, intersection of MyoD1, and Ascl1 and MyoD1). Although perturbations could be seen for many other factors as well, they were clearly most prominent for the MyoD1 program. This pronounced sensitivity of the MyoD1 induced program was also seen when myogenic genes were examined, as the expression of several known MyoD1 targets was strongly perturbed by the other factors (Fig 4D). By contrast, Ascl1, Sox2, and FoxA2 retained the potency to express marker genes of their respective linage during fate collision better (Fig 4D).

### Fate titration analysis reveals determinism of cell states driven by reprogramming factor levels

In relation to the collision effects described above, we speculated that under competitive conditions the relative expression levels of the transcription factors may influence the degree of perturbation. Thus, we devised “fate titration analysis” (FTA) to attribute transcriptomic states to reprogramming factor levels, modeling each cell as its own perturbation experiment with unique reprogramming factor expression levels and cell state readout (see Materials and Methods). Transcriptomic states were represented by a clustering label and this grouping was used to assign cells to either of the two opposing fates (Fig 4E). Strikingly, when applying FTA to collisions including our most potent single factor, MyoD1, we consistently found that only cells expressing very low levels of the perturbing factor converted to the MyoD1 transcriptional program (Fig 4E). However, even at relatively low levels of expression, the perturbing factor redirected the transcriptome to a collision state that was markedly different from the two individual fates and, in some cases, cells were even directed to the state of the perturbing factor (Fig 4E). This response seemed to be relatively independent of MyoD1 expression levels, as it occurred over the entire spectrum of MyoD1 expression (Fig 4E). Overall, these results indicated that the potential of reprogramming factors to induce global transcriptomic changes might be uncoupled from their sensitivity to perturbation.

The above FTA results also suggested that there may be determinism in gene expression states driven by reprogramming factor expression levels. One can think of determinism in the context of Waddington's landscape, describing not only the range of possible cell states but also their relative stability as a function of reprogramming factor expression levels. We characterized the degree to which cell states can be predicted in our data with classification models of cell states, such as logistic regression of cluster assignments (see Materials and Methods). Indeed, cluster assignment could be fit with 0.70 accuracy (0.96 ROC AUC, Fig 4F), showing that we could predict the general transcriptomic state of a cell based on its reprogramming factor expression levels, thereby demonstrating determinism in the system at the level of cluster identities A stronger demonstration of determinism is the ability of a mathematical model to extrapolate transcriptomic states to unseen conditions. We performed this extrapolation on the cell‐wise RNA vectors and were able to explain more variance with a nonlinear model than with a linear model (*R* ^2^ of 0.61 vs. 0.65, Fig 4G). Hence, outcome cell states are nonlinear functions of reprogramming factor expression levels. This notion fits well to the above‐described fate collisions, which yield cell states in combinatorial reprogramming that are not simply the sum of the induced fates. We further hypothesized that the transcription factor combinations did not only yield a predictable cell state for a given transgene expression level but also resulted in predictable cell state stability. Here, stability can be measured as gene expression variance where a low variance implies that a given transgene configuration instructs a very stable cell state. Indeed, we were able to correlate the modeled gene‐wise variance with prediction errors showing that variance estimation is possible here (*R* ^2^ = 0.90; Fig EV2D). When employing this, we found that variance does not increase with transgene levels, revealing that indeed each factor induces a stable transcriptomic program rather than a stochastic gene expression pattern (Fig EV2E–G). Lastly, we evaluated the ability of the identified rules of determinism to predict transcriptomic states in unseen genetic conditions (out‐of‐domain generalization). Expecting the generalizability of the fate decision‐making rules learned by this model to increase with the complexity of the training data, we first predicted cell states in unseen triple‐transgene conditions at an *R* ^2^ of 0.63 (Fig 4H). Indeed, the predictive performance on all models was increased on triple‐positive condition holdouts if the training data consisted of single‐ and double‐positive conditions rather than single‐positive conditions only (Fig 4I). Overall, this analysis suggested that the cell‐wise gene expression distribution is globally deterministic in Collide‐seq. The genetic circuits underlying fate decision‐making can be abstracted with supervised machine learning models as nonlinear effects that reflect synergism and antagonism of factors. Taken together, the factor collisions shown here demonstrated that all tested somatic reprogramming factors possessed the potency to perturb the others, with no truly dominant factor. Furthermore, this disturbance was highly deterministic, as generalizable gene expression patterns could be learned with supervised machine learning models, indicating that model‐aided design of reprogramming conditions for desired outcome fates is possible if enough gene expression data are provided.

### Fate competition between the bHLH factors MyoD1 and Ascl1

**Figure 5:**
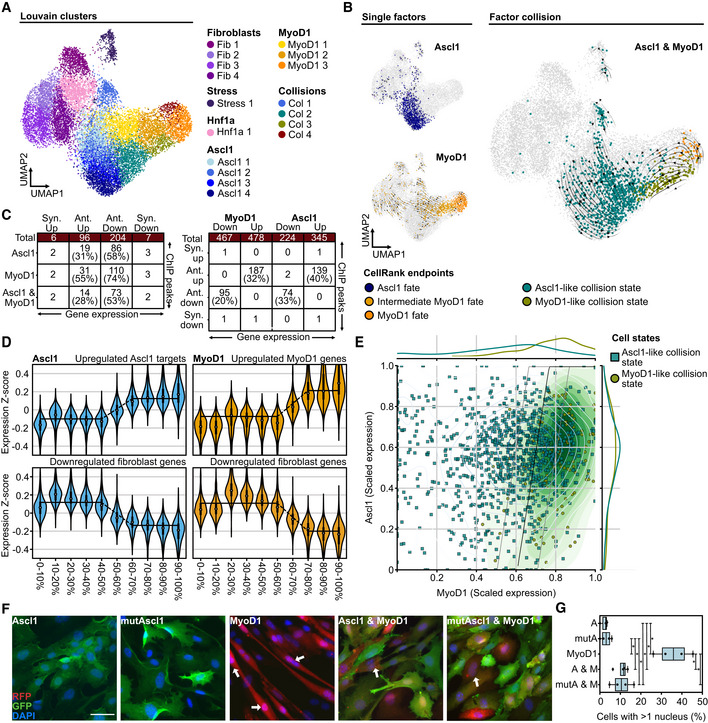
Fate competition between the bHLH factors MyoD1 and Ascl1 ALouvain clustering superimposed on a Uniform Manifold Approximation and Projection (UMAP) embedding of the second Collide‐seq experiment. Clusters are labeled according to the predominant transcription factor or cell state.BCondition‐based RNA velocity and CellRank for the indicated conditions. Shown are UMAP embeddings of all cells in the dataset (gray) with cells positive for the indicated transcription factor(s) colored by the terminal fate assigned by CellRank to indicate lineage endpoints (Bergen *et al*, 2020). See RNA velocity and CellRank analysis section of Materials and Methods for further details.CIntersections of genes bound by Ascl1 and/or MyoD1 based on ChIP‐seq data (Lee *et al*, 2020; see ChIP‐seq data analysis and synergism and antagonism annotation sections in Materials and Methods for further details) with gene sets that correspond to synergistic and antagonistic up‐ and downregulation. We defined synergism and antagonism based on differential expression analysis (See Differential expression analysis section in Materials and Methods for further details). Genes were considered synergistic or antagonistic when their expression in double‐positive conditions was higher (synergism) or lower (antagonism) than expected by adding their expression in the single factor conditions together. Shown is the size of the intersection (number of genes) and the relative size of this intersection with respect to the respective differentially expressed gene sets as defined by the column.DLog‐normalized expression z‐score of genes up‐ (top panels) and downregulated (bottom panels) by Ascl1 (left, blue violins) and MyoD1 (right, yellow violins) vs. their scaled expression levels (*x*‐axis) binned into 10% intervals. For each violin, the center dot represents the median, the centerline defines the range and the solid box marks the interquartile range (IQR).EFate titration plot of Ascl1 and MyoD1 double‐positive cells (see Fate titration section in Materials and Methods for further details). Shown are decision boundaries for indicated fates according to transcription factor levels. MyoD1 and Ascl1 expressions shown on the *x*‐ and *y*‐axis are log‐normalized expression values scaled into the dynamic range of the single‐positive condition (see Fate titration analysis section in Materials and Methods for further details). Cells are colored according to their lineage endpoint as determined by CellRank.FRepresentative immunofluorescence images of cells 3 days after transgene induction. Cells were stained for the fluorescent reporter present on the PiggyBac construct of the reprogramming factor. Arrows indicate multinucleated cells. Scale bar represents 50 μm. mutAscl1 = mutant Ascl1.GQuantification of the percentage of cells with more than one nucleus among all transfected cells within a given condition. Data points represent biological replicates (*n* = 4). For each box, the centerline defines the median, the height of the box is given by the interquartile range (IQR), the whiskers are given by 1.5 * IQR, and the outliers are given as points beyond the minimum or maximum whisker. Pairwise comparisons were performed with the Mann–Whitney *U* test and correction for multiple testing performed with Benjamini–Hochberg correction. *P < 0.05 (MyoD1 vs. Ascl1: *P* = 0.03, MyoD1 vs. mutAscl1: *P* = 0.03, MyoD1 vs. Ascl1 and MyoD1: *P* = 0.03, MyoD1 vs. mutAscl1 and MyoD1: *P* = 0.03, Ascl1 vs. Ascl1 and MyoD1: *P* = 0.03, Ascl1 vs. mutAscl1 and MyoD1: *P* = 0.03, mutAscl1 vs. Ascl1 and MyoD1: *P* = 0.03, mutAscl1 vs. mutAscl1 and MyoD1: *P* = 0.03). Source data are available online for this figure.

**Figure EV3:**
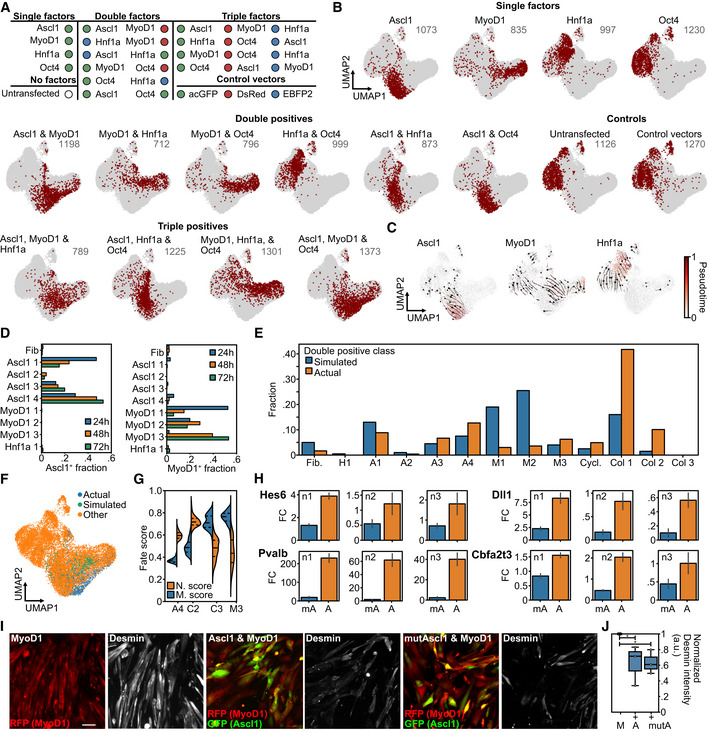
Investigation of Ascl1 and MyoD1 collision at three defined time points ASchematic overview of experimental conditions in second Collide‐seq experiment.BVisualization of assignment outcome (see Computational demultiplexing section in Materials and Methods for further details) for individual cells superimposed on a Uniform Manifold Approximation and Projection (UMAP) embedding of second Collide‐seq dataset. Depicted are cells positive for the indicated conditions and their total number.CCondition‐based RNA velocity trajectories. Shown are RNA velocities computed within single positive cells, projected on a UMAP embedding and computed on the transcriptomes of all cells (see RNA velocity and CellRank section in Materials and Methods for further details). Superimposed is the latent pseudotime (lineage progression coordinate) computed with scVelo (Bergen *et al*, 2020) in the context of the velocity computation.DRelative abundance of cells from Ascl1 (left) and MyoD1 (right) single‐positive conditions within Louvain clusters (*x*‐axis) colored by the time point of their collection.EFraction of cells per Louvain cluster colored according to whether they were actual or *in silico* simulated double‐positive cells.FSimulated double positives and actual double positives colored and superimposed on UMAP.GNeuronal (N.score) and myogenic (M.score) score for Ascl1 and MyoD1 double‐positive cells residing in collision states.HComparison of target gene induction by Ascl1 and mutAscl1 using qRT–PCR. Shown is the fold change of Ascl1 and mutAscl1 induced gene expression for the indicated genes as compared to untransfected fibroblasts 48 h after induction. Each panel represents a biological replicate (*n*1‐3). Primers used for qRT–PCR are listed in Appendix Table S2. Error bars represent standard deviation for technical replicates within each biological replicate. *n* = 3 biological replicates.IRepresentative images of Desmin fluorescence intensity after 72 h of transgene induction for the indicated conditions. Scale bars represent 100 μm. mutAscl1 = mutant Ascl1.JNormalized Desmin fluorescence intensity for indicated conditions (see Fluorescence intensity quantification section in Materials and Methods for further details). For each box, the centerline defines the median, the height of the box is given by the interquartile range (IQR), the whiskers are given by 1.5 * IQR, and the outliers are given as points beyond the minimum or maximum whisker. Pairwise comparisons were performed with the Mann–Whitney *U* test and correction for multiple testing performed with Benjamini–Hochberg correction. *P < 0.05 (MyoD1 vs. Ascl1 and MyoD1: *P* = 0.048, MyoD1 vs. mutAscl1 and MyoD1: *P* = 0.048, Ascl1 and MyoD1 vs. mutAscl1 and MyoD1 = 0.5). *n* = 3 biological replicates. Source data are available online for this figure.

Although each collision state is potentially different, we deemed following up the strong antagonistic interaction between MyoD1 and Ascl1 to be particularly interesting for several reasons. First, Ascl1 and MyoD1 are both bHLH factors that share several binding sites and interactors (Lee *et al*, 2020; de Martin *et al*, 2021). Second, our data indicated that Ascl1 does not only perturb the myogenic program induced by MyoD1, but also impairs fibroblast identity loss by the myogenic factor (Fig 4A). We thus set out to study their competition in more detail by setting up a second Collide‐seq experiment (Fig EV3A). For this experiment, we included a 24 and 48 h time point, in addition to our original 72 h time point, to get more insight into the temporal dynamics of fate collision. Furthermore, we chose to use Oct4 and Hnf1a, a fate determinant in liver development (Lau *et al*, 2018), over FoxA2 and Sox2 as they were expected to be less potent transcription factors, allowing us to investigate with little confounding how a third fate factor would influence Ascl1 and MyoD1 competition. Applying the same computational approach as above demultiplexed the data into the different experimental conditions (Fig EV3B) and Louvain clustering stratified the different transcriptomic states (Fig 5A). Distinct transcriptomic states for Ascl1, MyoD1, and Hnf1a only expressing cells could be detected and further confirmed by condition‐wise RNA velocity analysis (Figs 5A and EV3C). Moreover, a strong positive correlation between the velocity‐based pseudotime and the time these three reprogramming factors were expressed demonstrated that duration of expression, rather than expression levels, moves fate conversions forward (Fig EV3D). Concentrating our analysis on Ascl1 and MyoD1 only expressing cells, these cells were found in two separate groups of adjacent clusters, referred to as Ascl1 and MyoD1 clusters, respectively (Fig 5A). Cells expressing Ascl1, together with either Hnf1a or Oct4, were mostly found in one of the Ascl1 clusters (78% Ascl1 and Hnf1a, 84% Ascl1 and Oct4), while those that were positive for MyoD1 and either Hnf1a or Oct4 mostly resided in the MyoD1 clusters (75% MyoD1 and Hnf1a, 84% MyoD1 and Oct4; Figs 5A and EV3B). Cells expressing both Hnf1a and Oct4 mimicked the position of the Hnf1a single positives (Figs 5A and EV3B). This indicated a clear hierarchy between these four factors with Ascl1 and MyoD1 being most dominant, followed by Hnf1a and Oct4.

**Figure EV4:**
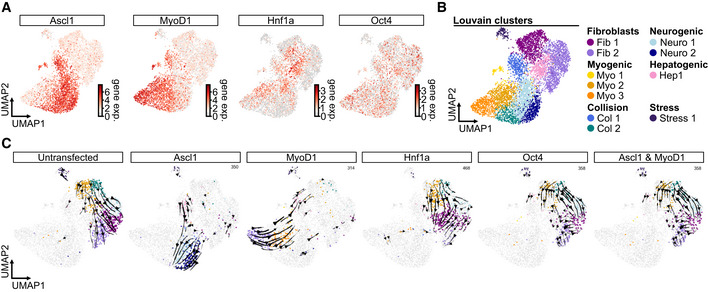
Validation of collision effects in a biological replicate ALog‐normalized transgene expression superimposed on Uniform Manifold Approximation and Projection (UMAP) embedding of separate 48 h dataset. Shown are total count‐normalized transgene expression levels (gene exp.) on a logarithmic (ln) scale.BLouvain clustering superimposed on UMAP embedding.CCondition‐based RNA velocity on a UMAP computed on cells from designated conditions. Shown is a UMAP of all cells in the dataset (gray). Superimposed are cells from the indicated condition colored in with the terminal fate assigned by CellRank (Lange *et al*, 2022), see RNA velocity and CellRank section in Materials and Methods for further details).

To better understand the collision of the two most dominant factors, Ascl1 and MyoD1, we next computed lineage endpoints using the previously calculated RNA velocity vectors in combination with CellRank (Lange *et al*, 2022, see Materials and Methods), grouping cells to shared fates in an unbiased manner. For Ascl1 and MyoD1 only expressing cells, a single unique lineage endpoint was found (Fig 5B). Using a large number of cells within the Ascl1 and MyoD1 lineages, together with the increased temporal resolution obtained by sampling at different time points (24, 48, and 72 h), we were able to stratify the previously identified collision states into two Louvain clusters (Fig 5B). Importantly, these transcriptomic states did not occur in the single transgene conditions and the predicted lineage endpoints were distinct from the single‐positive Ascl1 and MyoD1 endpoints (Fig 5B). Furthermore, *in silico* doublet simulation showed such cells would occupy very different transcriptomic states, suggesting that this collision state is not merely the average of the individual fates (Fig EV3E and F). We also confirmed that the occurrence of collision states between Ascl1 and MyoD1 is robust, as similar endpoints in a biological replicate dataset at 48 h were found (Fig EV4A–C). Importantly, these collision endpoints were dominant over the endpoints for both individual factors, abolishing the purely Ascl1 attractor and only leaving few cells at the purely MyoD1 attractor (Fig 5B). Furthermore, based on their overall similarity to the respective single factor lineages, one collision state was found to be more similar to the Ascl1 program, while the other was more similar to the MyoD1 program (Ascl1‐like intermediate, MyoD1‐like intermediate, Fig EV3G). Finally, overlapping our data with published ChIP‐seq data (Lee *et al*, 2020) confirmed that the co‐expression of Ascl1 and MyoD1 results almost exclusively in antagonistic or neutral effects concerning target gene expression, in accordance with our above findings on factor collision and despite using a different computational approach (Figs 4C and 5C).

Importantly, the better temporal resolution and improved sampling of the entire transgene expression spectrum in our second Collide‐seq experiment also allowed us to determine the relationship between reprogramming factor expression levels and the transcriptomic induction of new lineages. To this end, we first looked at general target fate regulation, determining the general correlation between transgene and target gene expression levels by binning cells according to their Ascl1 or MyoD1 expression levels and examining the target gene expression for each bin (Fig 5D). Strikingly, a clear gene expression threshold at which both Ascl1 and MyoD1 either induced or repressed the expression of their target genes was found (Fig 5D). Hence, both the activation and repression of target genes seems to behave in a binary fashion, rather than a linear or exponential manner. Consequently, above this threshold, transcriptomic states were similar between cells within a single condition and changed little with additional increases in the bHLH reprogramming factor level (Fig 5D).

Building on this, we probed how expression levels of Ascl1 and MyoD1 influence the decision between collision states, again using FTA (see Materials and Methods). This showed that only cells with very high MyoD1 expression moved towards the more MyoD1‐like collision state or the MyoD1‐only state (Fig 5E). At comparable levels of the respective transcription factors, cells mostly shifted into the Ascl1‐like collision state (Fig 5E). We quantified the degree of separation of both states with a linear classifier and were indeed able to distinguish cells from both states with high accuracy (0.72, Materials and Methods). This analysis illustrated the close call of fate competition by these very potent fate inducers: MyoD1 is dominant over Ascl1 at very high concentrations, but even low Ascl1 levels redirected a large proportion of cells to a more Ascl1‐like state. This balanced cell fate competition is particularly intriguing, given that neurons are developmentally more distant from fibroblasts, while muscle cells derive from the same germ layer.

### Phenotypic analysis reveals DNA binding independence of the colliding factor

In the above‐described results, we restricted our analysis to transcriptomic data on fate collisions. However, important readouts in reprogramming include changes in cellular morphology and function. For example, a major event in myogenesis is the fusion of newly formed myoblasts, giving rise to multinucleated myotubes (Sampath *et al*, 2018). To investigate whether the collision between Ascl1 and MyoD1 affected such functional aspects of reprogramming, we nucleofected MEFs with either Ascl1, MyoD1, or their combination and performed immunocytochemistry at 3 days post‐transgene induction (Fig 5F). Indeed, induction of MyoD1 resulted in the generation of a substantial amount of multinucleated cells with myotube‐like morphology (Fig 5F and G). By contrast, the addition of Ascl1 resulted in a significant reduction of multinucleated muscle‐like cell numbers (Fig 5F and G) and a marked decrease in the protein expression of muscle marker Desmin (Fig EV3I and J). To test whether this Ascl1‐mediated perturbation of the myogenic program is a consequence of the collision of the two transcriptional programs, or rather reflects competition between the two proteins themselves, we employed a mutant version of Ascl1 (mutAscl1) carrying two mutations in its basic domain: E131R132 to A131Q132 (see Materials and Methods). These residues are highly conserved in bHLH proteins and critical to DNA binding (Turner & Weintraub, 1994; Farah *et al*, 2000). Consequently, mutAscl1 displayed a strongly reduced ability to activate the expression of direct Ascl1 targets (Fig EV3H). However, using mutAscl1 for fate collision with MyoD1 showed that it was as capable as wild‐type Ascl1 in reducing both the number of multinucleated cells as well as Desmin expression levels (Figs 5F and G, and EV3I and J). Overall, these results indicated that phenotypic perturbations observed upon the collision of Ascl1 and MyoD1 seem to be independent of Ascl1 DNA binding.

### Competitive inhibition between Ascl1 and MyoD1 impairs pioneer factor activity

**Figure 6:**
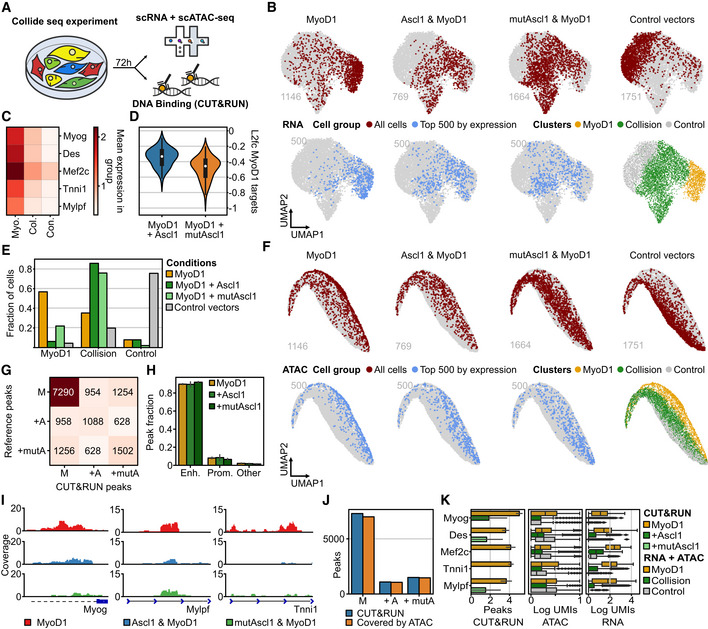
Competitive inhibition between Ascl1 and MyoD1 impairs pioneer factor activity ASchematic overview of third Collide‐seq experiment.BVisualization of assignment outcome (see Computational demultiplexing section in Materials and Methods for further details) for individual cells superimposed on Uniform Manifold Approximation and Projection (UMAP) embedding of the scRNA‐seq part of the experiment. Depicted are cells positive for the indicated conditions using all cells (top panels) and the top 500 highest transgene expressing cells (bottom panels). Bottom right panel depicts grouped Louvain clustering based on the factors present. MyoD1 = MyoD1 only, Collision = Ascl1 & MyoD1 and mAscl1 & MyoD1, Control = control vector carrying fibroblasts.CMatrixplot showing the relative expression of key myogenic genes between different clusters as indicated in Fig 6B. Myo = MyoD1, Col = Collision, Con = Control.DLog2 fold change of myogenic gene expression upon Ascl1 & MyoD1 (blue) and mAscl1 & MyoD1 (orange) collision as compared to MyoD1 only expressing cells. The number of data points per violin plot is the number of cells per matched condition shown in Fig 6B. For each violin, the center dot represents the median, the centerline defines the range and the solid box marks the interquartile range (IQR).EFraction of cells for each experimental condition among the different Louvain clusters.FVisualization of assignment outcome (see Computational demultiplexing section in Materials and Methods for further details) for individual cells superimposed on a UMAP embedding of the scATAC‐seq part of the experiment. Depicted are cells positive for the indicated conditions using all cells (top panels) and the top 500 highest transgene expressing cells (bottom panels). Bottom right panel depicts grouped Louvain clustering based on the factors present MyoD1 = MyoD1, Collision = Ascl1 & MyoD1 and mAscl1 & MyoD1, Control = Control vector carrying fibroblasts.GNumber of CUT&RUN peaks from a condition on the *y*‐axis overlapping with CUT&RUN peaks from a condition on the *x*‐axis. The presented number of overlapping peaks is the average over two CUT&RUN replicates of both conditions. (M = MyoD1, +A = Ascl1 & MyoD1, +mA = mAscl1 & MyoD1).HCUT&RUN peak classification averaged between two independent biological replicates depicted as a fraction of the total number of CUT&RUN peaks (Enh., Enhancers; Prom., Promoters). For each barplot, the 95% confidence interval is shown as error bars.IRepresentative Integrative Genome Browser (IGV, see CUT&RUN peak visualization in Materials and Methods for further details; Robinson *et al*, 2011) tracks for the indicated samples in CUT&RUN replicate 1 out of 2.JBar plot showing the number of averaged CUT&RUN peaks (blue) over two independent biological replicates that are covered by scATAC‐seq (orange).KCUT&RUN (left panel), scATAC (middle panel), and scRNA‐seq (right panel) signal for key myogenic marker genes for the indicated conditions. CUT&RUN signal is averaged for two biological replicates. The number of data points per barplot of the CUT&RUN data is two replicates, and per box plot of ATAC and RNA data is the number of cells per matched condition shown in Fig 6B. For each barplot, the 95% confidence interval is shown as error bars. For each box, the centerline defines the median, the height of the box is given by the interquartile range (IQR), the whiskers are given by 1.5 * IQR, and the outliers are given as points beyond the minimum or maximum whisker.

**Figure EV5:**
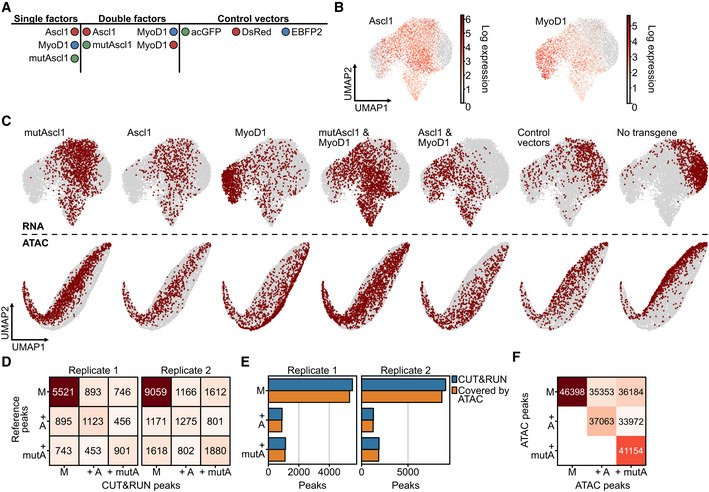
Multimodal analysis of Ascl1 and MyoD1 collision ASchematic overview of experimental conditions for third Collide‐seq experiment.BLog transgene expression superimposed on a Uniform Manifold Approximation and Projection (UMAP) embedding for Ascl1 (left panel) and MyoD1 (right panel). Shown are transgene expression levels on a logarithmic (ln) scale.CVisualization of assignment outcome (see Computational demultiplexing section in Materials and Methods for further details) for individual cells superimposed on scRNA‐seq UMAP embedding (top panels) and scATAC‐seq UMAP embedding (bottom panels). Depicted are cells positive for the indicated conditions.DNumber of CUT&RUN peaks from a condition on the *y*‐axis overlapping with CUT&RUN peaks from a condition on the *x*‐axis. The presented number of overlapping peaks is presented for both CUT&RUN replicates shown in Fig 6G.EBar plot showing the number of CUT&RUN peaks covered by scATAC‐seq for two biological replicates.FConfusion matrix showing the number of ATAC peaks in the different experimental conditions.

To further examine how Ascl1 and mutAscl1 perturb MyoD1 function, we set up a third Collide‐seq experiment including only Ascl1, mutAscl1, and MyoD1 (Figs 6A and EV5A) and performed joined single‐cell profiling of gene expression and chromatin accessibility (see Materials and Methods). As before, we first confirmed the detection of transgene expression and demultiplexed the dataset into its individual conditions (Figs 6B and F, and EV5B and C). In agreement with the cell reprogramming assays (Fig 5F), collision with either Ascl1 or mutAscl1 caused a marked decrease in myogenic gene expression (Fig 6C and D). Supporting this notion, only very few colliding cells were found in the MyoD1 cluster (Fig 6E). These transcriptomic states were paralleled by condition‐specific chromatin states, which also showed a disruption in double positives (Fig 6F). To further investigate whether this effect on cell fate collision is directly due to a disturbance of MyoD1 binding to its targets, we profiled DNA binding for MyoD1 using *cleavage under targets and release using nuclease* (CUT&RUN; Skene & Henikoff, 2017; Fig 6A). CUT&RUN for MyoD1 revealed an average of 7,290 binding sites (Figs 6G and EV5D). Fitting to the role of a developmental transcription factor, most of these binding sites were localized to gene regulatory elements, such as putative enhancer elements and gene promoters (Fig 6H). Strikingly, although the nature of bindings sites remained unchanged in collision conditions, a vast reduction in DNA binding was observed when MyoD1 collided with either wild‐type or mutAscl1 (Figs 6G and H, and EV5D). Indeed, coverage in the proximity of critical target genes revealed markedly reduced binding of MyoD1 upon collision with both Ascl1 and mutAscl1 (Fig 6I). As both Ascl1 and MyoD1 have been reported to act as pioneer transcription factors (Wapinski *et al*, 2017, Wapinski *et al*, 2013; Casey *et al*, 2018; Dall'Agnese *et al*, 2019), we hypothesized that Ascl1 might hamper the pioneer activity of MyoD1. Overlapping our CUT&RUN data with our scATAC‐seq and scRNA‐seq data for these factors showed a near‐perfect coverage of CUT&RUN peaks by cumulative scATAC‐seq peaks, confirming that the binding sites map to chromatin that is at least transiently open during the process (Figs 6J and EV5E). Since the presence of both Ascl1 and mutAscl1 significantly decreased the general capability of MyoD1 to bind to and open chromatin (Figs 6G and EV5F), which included several key myogenic lineage genes, we concluded that they impact MyoD1's ability to act as a pioneer factor (Fig 6K). Taken together, these results demonstrate that Ascl1 drives MyoD1 away from its binding sites and makes it unable to induce the expression of myogenic target genes, thus resulting in a fate collision state.

## Discussion

**Figure 7:**
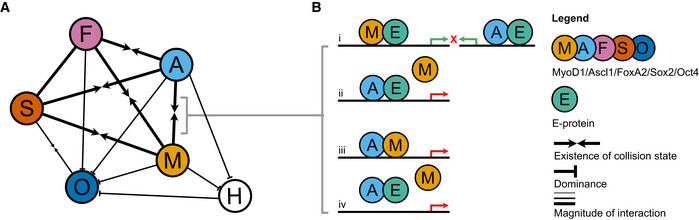
Proposed model ASchematic summary of factor hierarchies deducted from our experiments. Strength of interaction between factors is reflected by the line width. Nature of interaction (occurrence of collision state or dominance) is depicted by arrowheads (collision state) or bar ends (dominance).BSchematic overview of the possible mechanisms for Ascl1 and MyoD1 collision. (i) collision between transcriptional programs, (ii) competition between (shared) binding sites, (iii) formation of an inactive heterodimer, and (iv) cofactor competition.

Here, we have simultaneously compared and collided different cell fate conversions using Collide‐seq (Fig 7A). To our knowledge, a comparison of different reprogramming factors has, so far, only been performed with the aim of finding optimal conditions to drive cells towards a single given fate (Protze *et al*, 2012; Yang *et al*, 2019; Luginbühl *et al*, 2021). However, to what extent different factors achieve cell conversion through similar or different mechanisms has received little attention. Collide‐seq allowed exploring this question and revealed that there is a substantial difference in how different reprogramming factors erase the original identity (Fig 3F). This is interesting because it indicates that, although stable under physiological conditions, several different entry points exist to erase cell identity and characterizing and manipulating them might provide much‐needed strategies to improve existing reprogramming paradigms. Furthermore, fate erasure correlated well with factor potency overall, here quantified as perturbation magnitude in gene expression space with a linear model (Fig 3A). Strikingly, we found that the mesodermal factor MyoD1 was most potent in imposing its fate and repressing the original mesodermal fibroblast identity. This might seem surprising since not only MyoD1 but also Ascl1, FoxA2, Sox2, and Oct4 have been attributed pioneer factor activity and are all considered key drivers of their respective reprogramming paradigms (Iwafuchi‐Doi & Zaret, 2014; Zaret & Mango, 2016; Zaret, 2020; Sunkel & Stanton, 2021). Indeed, Ascl1 and MyoD1 are, on their own, sufficient to reprogram MEFs into induced neurons and muscles, respectively (Davis *et al*, 1987; Chanda *et al*, 2014). Additionally, Sox2 and Oct4 have been reported to individually reprogram MEFs into neural progenitor cells and IPSCs as well, albeit under specialized media conditions (Li *et al*, 2011; Ring *et al*, 2012). As such, we expected a similar ability between these factors to regulate their primary targets. Moreover, we predicted that nonmesodermal factors would silence more fibroblast genes due to larger differences in the active gene regulatory networks between starter and target cells. Instead, we find that nonmesodermal factors downregulate fewer genes as compared to the mesodermal factor MyoD1, suggesting that a special relationship between starter cells and reprogramming factors exists, possibly related to germ layer identity. In line with this notion is MyoD1's inefficiency to transdifferentiate cells into myocytes when a starter cell of nonmesodermal origin is used, indicating that MyoD1's potency might be indeed dependent on the cell of origin (Davis *et al*, 1987; Weintraub *et al*, 1989). Taken together, our results support the hypothesis that transdifferentiation into more closely related cell types might be more thorough and/or efficient (Hochedlinger & Plath, 2009; Morris & Daley, 2013). Therefore, therapeutic cell conversions may best be limited to related cell types if possible (Morris & Daley, 2013).

Interestingly, the strong reprogramming ability of MyoD1 on its own did not hold in competitive conditions. We discovered this surprising sensitivity of the pioneer factor through simultaneous induction of multiple divergent fates. MyoD1's program was effectively perturbed by all other factors, except Oct4, and this effect was largely independent of the expression levels of MyoD1 and the colliding factors (Fig 4E). Although we generally found antagonistic effects of colliding factors more frequently than synergism, one of the factors tested, Ascl1, was particularly disruptive and the only one capable of hindering MyoD1's potential to erase the fibroblast identity (Fig 4A). To our knowledge, these experiments are the first to systematically position different reprogramming factors against each other. Some of our findings are, however, reminiscent of the previously proposed “seesaw model” (Shu *et al*, 2013), in which opposing lineage factors are thought to cancel each other out, stabilizing a pluripotent stem cell identity. Conceptually close as well are cell fusion experiments (Cowan *et al*, 2005; Brown & Fisher, 2021). Such experiments showed that pluripotency is often dominant over somatic cell identities. However, our fate collision showed no dominance of pluripotency programs, nor did we detect any hierarchy that would reflect a developmental order. This indicates that competition is probably resolved on the factor rather than the lineage level. Similarly, several studies have suggested that induction of pluripotency factors might be an effective way to remodel the epigenome and make differentiated cells more amenable to fate changes (Deleidi *et al*, 2011; Kim *et al*, 2011; Peskova *et al*, 2019; Sharma *et al*, 2019). However, our data shows that Oct4 and Sox2 do not act as immediate cell fate enablers enhancing the transcriptional effects of the somatic reprogramming factors (Figs 4C, and EV2A and B). Instead, Oct4 affected the individual somatic reprogramming trajectories very little during fate collision. These findings are in line with studies showing that, during the early phases of iPSC reprogramming, Oct4 overexpression mostly leads to off‐target gene expression patterns (Velychko *et al*, 2019). Moreover, the same authors also found that, when using the Yamanaka factors, Oct4 has a neglectable role in cell fate erasure, writing “loss of fibroblast identity […] appears to be independent of exogenous Oct4.” This also fits with the concept that Oct4 might create a more open and instable chromatin environment that facilitates the actions of other factors (Kim *et al*, 2011), a process that could be rather slow (Taberlay *et al*, 2011) and hence not detectable during the first 72 h examined here. This would also explain why Sox2 and Oct4 double‐positive cells show only few regulated targets associated with pluripotency, although two‐factor iPS reprogramming of fibroblasts has been conducted before (Huangfu *et al*, 2008; Kim *et al*, 2008; Nemajerova *et al*, 2012).

Collide‐seq also enabled us to quantify the influence of reprogramming factor expression levels on cell conversion. Leveraging this with a machine learning approach, we were able to reveal a high degree of determinism in this system by demonstrating that the induced perturbations yield predictable cell states. We also characterized transcription factor abundance‐dependent fate choices with fate titration analysis, showing that Collide‐seq can be used to titrate transcription factors against each other to screen fate outcomes. Interestingly, the resulting transcriptomic states were found to be a nonlinear function of the reprogramming factor expression levels, which is both highlighted by the emergence of collision states in fate titration analyses and the step‐like saturating dependency of gene activation of transcription factor induction (“binary switch”). In other words, cell fate conversion does not scale gradually with increasing reprogramming factor expression levels but instead depends on whether a critical expression threshold is reached. This observation is highly reminiscent of a previously described switch system in blood cell differentiation, where transcriptomic changes corresponding to cell fate decisions also occur abruptly rather than gradually (Huang *et al*, 2007). Taken together, this finding indicates that an optimal level of reprogramming factors expression exists, which is sufficient for reprogramming but also minimizes the risk of failure due to cellular stress as a result of transcription factor overexpression. Thus, identifying optimal reprogramming factor expression levels could significantly improve existing reprogramming protocols. Furthermore, the cell‐wise perturbation effects described here may also be leveraged for gene regulatory network modeling, as they tend to be stronger than available data based on single‐cell knock‐out data. Hence, they may facilitate the development of bottom‐up models for fate collision on the chromatin and transcriptome level in the future (preprint: Kamimoto *et al*, 2020).

Overall, our study unraveled several key principles of cellular reprogramming by systematically comparing reprogramming factors and applying collisions between them, thereby revealing a deterministic system that mapped the dominance or comparable strength of the different factors (Fig 7A). For example, the ability of Ascl1 to induce its lineage and remove fibroblast identity was similar to FoxA2 and Sox2 (Fig 3A and E). MyoD1, on the other hand, was found to be considerably more potent (Fig 3A and E). However, this potency of MyoD1 was not reflected under competitive conditions, as their relative strengths reversed, making Ascl1 the strongest driver that directed the majority of cells towards an Ascl1‐like intermediate (Fig 5E). Correspondingly, we also observed a clear loss of MyoD1 pioneer activity upon collision with Ascl1 (Fig 6G, J and K). Together, these results revealed a profound effect of competition on MyoD1 function and prompted us to further investigate how Ascl1 achieves this. To this end, it is important to note that Ascl1 and MyoD1 are both basic helix loop–helix (bHLH) factors, which can function as either homo‐ or heterodimers (Massari & Murre, 2000; Wang & Baker, 2015; Murre, 2019; de Martin *et al*, 2021). Consequently, we considered four molecular mechanisms to explain these effects: (i) collision between transcriptional programs, (ii) competition between (shared) binding sites, (iii) formation of an inactive heterodimer, and (iv) cofactor competition (Fig 7B). Interestingly, we found that collision is hardly diminished by employing mutAscl1 (Figs 5F and EV3H), indicating that fate collision is likely not a result of transcriptional competition or DNA binding site competition. An alternative explanation might therefore be that Ascl1 and MyoD1 form nonfunctional heterodimers that bind DNA but do not activate transcription. Our CUT&RUN data, however, shows that many MyoD1 binding sites are lost upon collision with Ascl1, making this option also improbable. Thus, competition for possible dimerization partners appears to be the most plausible explanation for the observed effects (Fig 7B). Indeed, competition on this level has already been suggested for MyoD1 and the bHLH factor Twist (Spicer *et al*, 1996). The finding that the fate collision between MyoD1 and Ascl1 is triggered by competition between the factors themselves rather than their interaction with DNA or their programs is highly relevant for the design of novel reprogramming approaches since it indicates that the master transcription factors expressed in the cell of origin rather than the molecular abilities of the expressed reprogramming factors might define whether certain factors possess reprogramming potential in certain settings. It is important to note, however, that not all detected fate collisions might be caused by the same mechanism and that we unfortunately know very little about the factors that protect and define fibroblast identity. Taken together, we investigated fundamental concepts of cell identity conversions using a combination of novel machine learning and molecular biology approaches. These results exposed the underlying principles of cell identity acquisition that could be used to improve current reprogramming strategies through the informed selection of starter cells, cell fate factors, and reprogramming factor expression levels.

## Materials and Methods

### Animals

R26‐M2rtTA knock‐in mice were obtained from Jackson Laboratory (RRID:IMSR_JAX:006965) and maintained in pathogen‐free conditions with 12 h light/dark cycles. Mice were housed in groups of 2–5 animals and had free access to water (acidified and desalinated) and standard rodent chow (Altromin, 1,310 M). Mice were kept as homozygous for the knock‐in. All experimental procedures in this study, performed at the LMU Munich, were in accordance with German and European Union guidelines and approved by the government of Upper Bavaria (Germany) where necessary.

### Mouse embryonic fibroblasts isolation and culture

Mouse embryonic fibroblasts were obtained from E14.5 embryos of R26‐M2rtTA knock‐in mice. Using a dissection microscope (Leica), heads, limbs, vertebral columns, and internal organs were removed to make certain no multipotent cells were present in cultures. After dissection, 2–3 embryos were pooled, and tissue was dissociated in 0.15% Trypsin (Gibco) for 10–15 min to obtain single‐cell suspensions. Cells were plated in a single T75 tissue culture flask per embryo in MEF medium at 37°C and 5% CO_2_ (Dulbecco's Modified Eagle Medium (Gibco #61965) supplemented with 10% FBS (Pan Biotech or Gibco), 1% Sodium Pyruvate (Gibco), 1% HEPES (Gibco) and 1% Penicillin/Streptomycin). Cells were split once at a 1:3 ratio when confluent before freezing. After thawing, cells were grown in T75 culture flasks until confluent before nucleofection.

### Nucleofection

Nucleofections were performed according to the manufacturer's instructions (Lonza, P3 Primary Cell 4D‐Nucleofector™ X). Briefly, 5.0 × 10^5^ cells were counted and spun down at 200 × *g* for 10 min, the supernatant was discarded, and cells were resuspended in 100 μl nucleofection solution master mix (82 μl P3 Primary Cell Nucleofector Solution + 18 μl Supplement 1). Upon resuspension, cells were immediately transferred to previously prepared 1.5‐ml tubes containing DNA mixtures and subsequently transferred to 100 μl Nucleocuvettes. Nucleofection was performed using the CZ‐167 program and 500 μl of prewarmed RPMI 1640 was added immediately after nucleofection (Gibco; 1% Penicillin/Streptomycin). Cells were transferred to a 37°C and 5% CO_2_ incubator for 10 min to recover. Finally, cells were plated in a single well of a 0.1% Gelatin in PBS (ROTI®Cell, Carl Roth) coated 12‐well plate with 1 ml of preconditioned MEF medium and cultured at 37°C and 5% CO_2_. In general, 1 μg of DNA per transcription factor and 2 μg of transposase construct were used for nucleofections. However, for qRT–PCR experiments aimed at quantification of Ascl1 and mutAscl1 downstream targets, transposase was omitted. For expansion, cells were subcultured on 0.1% Gelatin culture vessels at 37°C and 5% CO_2_ until the appropriate cell number for the experiment was reached. For immunocytochemistry experiments, cells were plated directly onto Poly‐D‐Lysine coated glass coverslips.

### Piggybac vector generation

To generate TetOn inducible Piggybac expression vectors for transgene expression, first, a dCas9 expression cassette was removed from PB‐dCas9‐T2A‐GFP‐PolyA‐Blasticidin (A. Köferle, unpublished) through cutting with *SpeI* and *Bsu36I* (New England Biolabs). Next, a Tet Response Element (TRE) followed by a minimal CMV promoter was amplified from pLV‐TetO‐Oct4 (pLV‐tetO‐Oct4 was a gift from Konrad Hochedlinger, Addgene plasmid # 19766; http://n2t.net/addgene:19766; RRID:Addgene_19766) using the primers TetOn Fwd and TetOn Rev (Appendix Table S4). Combining insert and backbone at a ratio of 3:1 in combination with the Gibson Assembly Mastermix (NEB, E2611S) for 30 min at 50°C, PB TetO PolyA was generated. Subsequently, a CMV enhancer element and CMV promoter driving the expression of either acGFP1 or EBFP2 were amplified from AcGFP1‐C1 (AcGFP1‐C1, was a gift from Michael Davidson, Addgene plasmid #54607, http://n2t.net/addgene:54607; RRID: Addgene_54607) and EBFP2‐C1 (EBFP2‐C1 was a gift from Michael Davidson, Addgene plasmid #54665, http://n2t.net/addgene:54665, RRID: Addgene_54,665) using Colors Fwd in combination with acGFP Rev or EBFP2 Rev, respectively (Appendix Table S4). Using the same Gibson Assembly procedure as described above, both inserts were combined with PB TetO PolyA to generate PB TetO acGFP PolyA and PB TetO EBFP2 PolyA. DsRed Express 2 was amplified from pCAG‐Ascl1‐IRES‐DsRed (Heinrich *et al*, 2010) using the primers DsRed Fwd and DsRed Rev, and the CMV enhancer and promoter were amplified from EBFP2‐C (described above) using the primers CMV Fwd and CMV Rev and combined with PB TetO PolyA to generate PB TetO DsRed PolyA (Appendix Table S4). Next, an SV40 polyadenylation cassette was amplified from the AcGFP1‐C1 plasmid using the primers SV40 Fwd (DsRed) or SV40 Fwd (acGFP and EBFP2) with SV40 Rev and combined with all the respective fluorescent reporter backbones to generate PB TetO PolyA acGFP PolyA, PB TetO PolyA EBFP2 PolyA and PB TetO PolyA DsRed PolyA through Gibson assembly (Appendix Table S4). Finally, Ascl1 was amplified from pCAG‐Ascl1‐IRES‐DsRed (Heinrich *et al*, 2010), MyoD1 was amplified from pCAG‐MyoD1‐IRES‐GFP (in‐house), Oct4 was amplified from pLV‐TetO‐Oct4 (see above), Sox2 was amplified from pCAG‐Sox2‐IRES‐GFP (in‐house), FoxA2 was amplified from pLV‐PGK‐FoxA2 (pLV.PGK.mFoxa2 was a gift from Malin Parmar, Addgene plasmid # 33014; http://n2t.net/addgene:33014; RRID:Addgene_33,014) and Hnf1a was amplified from E14.5 mouse liver cDNA. E14.5 mouse liver cDNA was obtained by tissue digestion in TRIzol (Invitrogen) according to manufacturer's instructions and subsequently performing a reverse transcription of 100 ng input total mRNA using the Maxima First Strand cDNA Synthesis Kit (Thermo Fisher Scientific; Appendix Table S4). The inserts of the respective factors were combined with all three fluorescent reporters carrying PiggyBac backbones, predigested with *Mfe1* (New England Biolabs), to generate PB TetO TF PolyA acGFP/EBFP2/DsRed PolyA, for all transcription factors using Gibson Assembly. For FoxA2 and Sox2, two AU‐rich elements were cloned in between the cDNA and polyA site by digesting both backbones with *Mlu1* (New England Biolabs) and combining it with a PCR‐amplified insert of AU‐repetitive sequences (Appendix Table S4) generated by amplification with AU Fwd and AU Rev from a custom DNA oligo (Appendix Table S4). To generate a 3xFLAG tagged MyoD1 construct, MyoD1 was amplified from PB TetO MyoD1 acGFP after digestion with *Hpa1* and *Kpn1* (New England Biolabs) using the MyoD1 FLAG Fwd and MyoD1 FLAG Rev primers (Appendix Table S4). A 3xFLAG repeat was amplified from a custom‐ordered DNA oligo using the FLAG Fwd and Flag Rev primers (Appendix Table S4). Combining both inserts with the digested fluorescent reporter PiggyBac backbones mentioned above yielded PB TetO 3xFLAG MyoD1 PolyA acGFP/EFBP2/DsRed PolyA. To generate mutAscl1, point mutations E131R132 to A131Q132 were introduced in PB TetO Ascl1 acGFP using the site‐directed mutagenesis kit (New England Biolabs). All cloning primer sequences are provided in Appendix Table S4.

### Transgene induction

Transgene expression was induced by the administration of doxycycline (2 μg/ml) every 24 h until the indicated time points, considering its ±22 h half‐life (Cunha *et al*, 2000). The culture medium was replaced by fresh doxycycline‐containing medium for each treatment. For immunocytochemistry experiments, transgene expression was induced the day after nucleofection, and a nontreated sample of the same condition was used as a control. For single‐cell experiments and quantitative polymerase chain reaction (qRT–PCR) experiments aimed at determining reprogramming factor expression levels, cells were first expanded to at T75‐T175 before sorting (see below) and induction of transgene expression. A nontreated sample of the same condition was used as a control. For qRT–PCR experiments aimed at quantification of Ascl1 and mutAscl1 downstream targets, transgene expression was induced the day after nucleofection and a treated sample of untransfected cells was used as a control.

### Immunocytochemistry and image acquisition

For immunocytochemistry, cells were fixed at the indicated time points with 4% paraformaldehyde in phosphate‐buffered saline (PBS) for 10–15 min. Cells were permeabilized by incubation with a blocking solution (3% BSA, 0.5% Triton‐X 100 in PBS) for 30 min. Primary antibodies were incubated in a blocking solution at 4°C overnight or for 2 h at room temperature (Appendix Table S3). Cells were thoroughly washed with PBS to rinse off the primary antibody solution. Secondary antibodies (including DAPI) were incubated in the dark at room temperature for 1 h in a blocking solution followed by thorough washing with PBS (Appendix Table S3). For cells plated on coverslips, coverslips were mounted on glass slides using a water‐based nonfluorescent mounting medium (Aqua Poly/Mount (Polysciences, Warrington, PA)). Stained cells were analyzed using an AxioM2 or Axio Observer epifluorescence microscope (Carl Zeiss) for coverslips and culture plates, respectively. Images were obtained using the ZEN2 Software (Carl Zeiss).

### Fluorescence intensity quantification

To compare Desmin protein expression levels between conditions, images were acquired as described above using identical exposure times within an experiment. Intensities were quantified by loading images with Fiji (Schindelin *et al*, 2012) and generating a mask for each cell using *Image > Adjust > Threshold*. Next, the mean intensity within the masked area was measured using *Analyze > Measure*. For each image, a total of five equally sized regions without any clear Desmin signal were used to determine background levels and their mean was subtracted from the average measured intensity. To compare different experiments, intensity levels were normalized with respect to the MyoD1 only condition.

### Fluorescence‐activated cell sorting

Cells were trypsinized, collected in prewarmed MEF medium, and washed once in PBS. The supernatant was discarded, and cells were resuspended in 1 ml PBS supplemented with 10% FBS before being transferred to FACS tubes by passing through a 40 μm cell strainer. Cells were sorted using a BD FACSARIAIIIu cell sorter (BD Biosciences). Gates were set using untransfected MEFs as a reference. Cells were sorted at flow rates between 2 and 3 (arbitrary units, corresponding to ∼17–25 μl/min) and collected in 1.5‐ml tubes containing 300 μl MEF medium supplemented with 10% additional FBS. For quantification of reprogramming factor levels, 37,500–100,000 cells were sorted per condition and plated in a single well of a 24‐well plate before inducing transgene induction with doxycycline the next day (see above). For single‐cell experiments, 7,000/10,000 cells for each transfected condition and 5,000/7,500 untransfected MEFs were sorted. After sorting, all cells were pooled in a single 15‐ml conical tube and centrifuged at 300 × *g* for 5 min. One milliliter of supernatant was left and supplemented with fresh MEF medium. Cells were plated by taking 1 ml of this suspension containing between 50–60,000 cells in a single 24‐well. For quantification of Ascl1 and mutAscl1 target expression levels, 50,000–100,000 cells per condition were sorted 48 h after induction and immediately processed for RNA isolation (see below).

### RNA extraction and quantitative polymerase chain reaction (qRT–PCR)

For RNA extraction, cells were collected 48 h after transgene induction and RNA was isolated using the ARCTURUS® PicoPure® RNA Isolation Kit (Applied Biosystems) according to the manufacturer's instructions. Genomic DNA was removed using the On‐Column DNase I Digestion Set (Sigma‐Aldrich). For retrotranscription, equal amounts (between 50–100 ng) of total RNA were retrotranscribed using the Maxima First Strand cDNA Synthesis Kit (Thermo Fisher Scientific). First‐strand cDNA was diluted 1:5 in RNAse‐free water and 5 μl was used for each qRT–PCR reaction. qRT–PCR experiments were performed on a QuantStudio 6 (Applied Biosystems) using PowerUp™ SYBR™ Green Master Mix (Applied Biosystems). The expression of each gene was determined in triplicate and relative expression determined using the ΔΔCt method (Livak & Schmittgen, 2001). qRT–PCR Primers are listed in Appendix Table S2.

### *In vitro* reprogramming

For reprogramming experiments, cells were nucleofected as described above and expanded on 0.1% gelatin‐coated culture vessels. After expansions, single‐ (Ascl1, mAscl1, and MyoD1) and double‐positive cells (Ascl1 and MyoD1, mAscl1 and MyoD1) were sorted and plated at a density of ∼12,500 cells per well in a 96‐well plate. The next day, the medium was changed and transgene expression induced as described above. Cells were treated with doxycycline every 24 h and fixed 3 days postinduction for immunochemistry (see above).

### Droplet‐based scRNA‐seq and scATAC‐seq

For scRNA‐seq only samples, cells were trypsinized and collected in a prewarmed MEF medium at the indicated time points. Cells were centrifuged at 300 × *g* for 5 min to remove debris and resuspended in 50–100 μl PBS for cell counting. Cell suspensions were counted, and suspension volume was adjusted to contain approximately 1,000 cells per μl. For a targeted retrieval of 10,000 cells, ∼17,500 cells were loaded, and libraries were prepared using the Chromium Single Cell 3′ Reagent Kits v2 (24, 48, 72 h data with Ascl1, MyoD1, Hnf1a, and Oct4) or v3 (72 h data with Ascl1, MyoD1, FoxA2, Sox2, and Oct4) according to the manufacturer's instructions. Libraries prepared with v2 chemistry were sequenced on a HiSeq4000 whereas v3 libraries were sequenced on a NovaSeq6000. All libraries were sequenced with a 100 bp paired‐end configuration.

For scMultiome (scRNA‐seq + ATAC‐seq) samples, cells were trypsinized and collected in a prewarmed MEF medium 72 h after induction. The cell suspension was centrifuged at 300 × *g* for 5 min at 4°C and resuspended in 50 μl of PBS + 0.04% BSA (Miltenyi Biotec). Cells were pelleted once more by centrifugation at 300 × *g* for 5 min at 4°C. Forty‐five microliter of supernatant was removed and an equal amount of lysis buffer (10 mM Tris–HCl pH 7.4, 10 mM NaCl, 3 mM MgCl_2_, 0.1% Tween‐20, 0.1% Nonidet P40 Substitute, 0.01% Digitonin, 1% BSA, 1 mM DTT, 1 U/ul H_2_O) was added. Cells were lysed on ice and after 5 min 50 μl of wash buffer (10 mM Tris–HCl pH 7.4, 10 mM NaCl, 3 mM MgCl_2_, 1% BSA, 0.1% Tween‐20, 1 mM DTT, 1 U/μl RNAse inhibitor) was added without mixing. Nuclei were pelleted by centrifugation at 500 × *g* for 5 min at 4°C. Ninety‐five microliter of supernatant was removed, taking care not to disturb the pellet, and 45 μl of diluted nuclei buffer (1× Nuclei Buffer (10× Genomics), 1 mM DTT, 1 U/μl RNAse inhibitor) was added without mixing. Nuclei were spun down once more at 500 × *g* for 5 min at 4°C and all supernatant was removed. Nuclei were resuspended in 7 μl of ice‐cold nuclei buffer and 2 μl of nuclei suspension was mixed with 8 μl of diluted nuclei buffer and 10 μl Trypan Blue to determine nuclei concentration. For a targeted retrieval of 10,000 nuclei, the nuclei suspension was diluted to a concentration between 3,280 and 8,060 nuclei per μl. Libraries were prepared using the Chromium Next GEM Single Cell Multiome ATAC + Gene Expression kit according to the manufacturer's instructions and sequenced on an Illumina NovaSeq 6000 sequencer.

### Cleavage under targets and release using nuclease (CUT&RUN)

For CUT&RUN assays, 3xFLAG‐MyoD1 (see above) was used to allow MyoD1 pulldown and DNA binding assessment. The assay was performed using the CUT&RUN assay kit (Cell Signaling Technologies, 86652) according to the manufacturer's instructions. Briefly, 100,000 cells per reaction were collected and bound to Concanavalin A Magnetic beads. Cells were permeabilized and incubated with a primary antibody against FLAG for 2 h at 4°C (Appendix Table S3). Subsequently, cells were incubated with pAG‐MNase for 1 h at 4°C. pAG‐MNase was activated by adding calcium chloride and a 30 min incubation at 4°C. Stop buffer (Cell Signaling Technologies) with 10 pg of Spike‐In DNA (Cell Signaling Technologies) was added to each sample to stop the reaction and obtain normalization reads after sequencing. DNA was purified using phenol/chloroform extraction and ethanol precipitation as described in the manufacturer's protocol. Input samples were generated by collecting 100,000 cells per condition and incubating with DNA extraction buffer (Cell Signaling Technologies) at 55°C for 1 h shaking at ∼750 rpm on a ThermoMixer. Afterwards, samples were cooled down and sonicated with a BioRuptor® Pico (Diagenode) using 10 sets of 30‐s pulses. Fragmented chromatin was isolated together with CUT&RUN samples as described above.

### CUT&RUN Library preparation

DNA sequencing libraries were generated using the SimpleChIP® ChIP‐seq DNA Library Prep Kit for Illumina (Cell Signaling Technologies, 56795) and SimpleChIP® ChIP‐seq Multiplex Oligos for Illumina® (Dual Index Primers, Cell Signaling Technologies, 46538) following the manufacturer's instructions and adapting the protocol where needed as instructed by the manufacturer in the CUT&RUN Assay kit protocol. Briefly, an equal amount of DNA was used for all CUT&RUN and input samples and DNA ends were prepared for adaptor ligation. Note that here incubation temperature was lowered from 65 to 50°C according to the manufacturer's instructions. Next, adapters were ligated to the DNA. Finally, DNA was amplified using PCR and Dual Index primers for Illumina® (Cell Signaling Technologies, 47538). Importantly, anneal and extension time was reduced from 75 to 15 s to avoid amplification of large library fragments per the manufacturer's instructions. Furthermore, all clean‐up steps were performed with 1.1× volume of SPRIselect® beads to increase the capture of smaller DNA fragments. Generated libraries were pooled and sequenced using the MiSeq Reagent Kit v3 and a 2 × 75 bp paired‐end sequencing strategy on an Illumina® MiSeq sequencer.

### Preprocessing of sequencing data

For the alignment of reads, we used the sequences and annotation files for the Mouse genome (GRCm38) from Ensembl (release 97). The synthetic transcription factors and reporter sequences and a custom‐generated annotation were appended to the genome sequence and annotation. The Cell Ranger software (version 3.1.0) run with the command “cellranger mkref” created an index of the genome. The Cell Ranger pipeline run with the command “cellranger count” aligned the reads, generated QC metrics, estimated the number of valid barcodes, and created the count matrices. The command was executed with standard parameters, except that we adjusted the number of expected cells and the chemistry parameter accordingly. AnnData objects of the raw and filtered count matrices were created using the Python package Scanpy (Wolf *et al*, 2018, version 1.4.4). The alignment files (bam) were sorted by barcode using samtools (Li *et al*, 2009, version 1.10) and Velocyto (la Manno *et al*, 2018, version 0.17.17) was run using the “velocyto run” command with the filtered barcodes list from the Cell Ranger run.

### Processing of CUT&RUN data

The CUT&RUN data were processed using the Nextflow‐based ChIP‐seq workflow from nf‐core (version 1.2.2). For the different samples, a single pseudo antibody was defined and the samples of the same conditions sequenced in multiple experiments were defined as replicates. The computational pipeline was run in two independent executions to align against the yeast genome (R64‐1‐1) and against the mouse genome (GRCm38). The aligned reads from the yeast alignment were used for normalization as per the manufacturer's instructions found here: https://www.cellsignal.com/learn‐and‐support/protocols/cut‐and‐run‐protocol.

### CUT&RUN peak visualization

For visualization of CUT&RUN peaks, CUT&RUN data were processed as described above. Processed data were loaded into Integrative Genomics Viewer (IGV, version 2.11.4; Robinson *et al*, 2011). To compare several samples, each sample was loaded as an individual track. For exporting images, *y*‐axes were set to the same scale before exporting.

### Processing of single‐cell multiome data

The single‐cell Multiome‐Seq data were processed using the software cellranger‐arc (version 2.0.0), run with the cellranger‐arc count command with standard parameters and the corresponding genome. To build the reference genome index for mice, GRCm38 the annotation from Ensembl (release 97) was used. The sequences and custom annotations of the synthetic transcription factors and fluorophores were appended to the genome fasta and annotation files. The samples processed with cellranger‐arc count were aggregated using the cellranger‐arc aggr command with the ‐‐normalize = none parameter.

### Generation of a modified gene annotation

The sequences of the overexpressed reprogramming factors differ from the endogenous loci in only a short interval in their UTRs. To distinguish between endogenous and transgene expression, we created synthetic versions of the endogenous genes by replacing their UTRs with the UTR sequences of the transgenes (from Gencode version vM20, https://www.gencodegenes.org), and appended them to the gene annotation. This modified annotation was then used for reading alignment by the Cell Ranger pipeline as described above (Preprocessing of sequencing data). The Cell Ranger pipeline only considers reads that are compatible with a single gene annotation, and therefore, only reads specific to the UTRs of the endogenous genes or of the transgenes are used for UMI counting.

### Computational demultiplexing

The exact analysis is documented in Notebooks I‐1, II‐1, and III‐1 on GitHub (https://github.com/theislab/collideseq_reproducibility). We first grouped cells into induced and noninduced for each transgene and fluorophore by defining an expression threshold for each factor (maximum likelihood assignment of cells to centroids). Secondly, we assigned cells to the condition of their posterior probability, using the initial assignments to define the prior distribution of transgene counts in induced cells (Appendix Fig S2B, the posterior membership probability). This second step allowed us to incorporate the prior knowledge that only a subset of all possible transgene combinations was included in this setup.

We defined condition centroids with two separate models, that yielded similar results in the end. Firstly, we used a gradient‐free optimization algorithm to infer optimal decision boundaries θ between the active and inactive component for each transgene in a joint optimization problem, using the deviation between the inferred distribution of cells over conditions *f* and the input distribution *x* as an objective *l*:

$$l=\left|f\left(\theta \right)-x\right|$$

Here, the maximum likelihood assignment of a cell to a condition $\hat{c}$ consists of its classification based on the decision boundaries:

$$\hat{c}=1\mathrm{if\theta}>\text{xelse}0$$

Secondly, we defined centroids with a 2‐component Gaussian mixture model for each transgene separately. Here, the maximum likelihood assignment of a cell to a condition consists of its assignment to a mixture *k*:

$$\hat{c}=\arg\underset{k}{\max}P\left(x|\mu_{k},\sigma_{k}\right)=\arg\underset{k}{\max}-\log\left(\sigma_{k}\right)-\log\left(2\pi \right)-\frac{1}{2}\frac{{\left(x-\mu_{k}\right)}^{2}}{\sigma_{k}{}^{2}}$$

These mixture models showed the collapse of the inactive component to zero in a couple of cases, in which we then augmented the inferred threshold to achieve similar results to the gradient‐free optimization method.

Posterior membership probability: The maximum likelihood assignments proposed above cannot guard against the assignment of cells into conditions that do not exist, such as a condition with all transcription factors active and all colors inactive. Due to noise in the data, such conditions may indeed be the maximum likelihood estimator of condition membership. However, we have the prior knowledge that only a subset of all transgene conditions exists in the data. We defined a partition function containing only the input conditions K and defined a posterior probability distribution over input conditions using this partition function and Gaussian likelihoods *P* to then assign cells to their maximum a posteriori estimate of membership:

$$\hat{c}=\arg\underset{k}{\max}\frac{P\left(x|\mu_{k},\sigma_{k}\right)}{\sum_{k\subset K}P\left(x|\mu_{k},\sigma_{k}\right)}$$

### Unsupervised analysis of single‐cell RNA‐seq data

The exact analysis is documented in code in Notebooks I‐1, II‐1, and III‐1 on GitHub (https://github.com/theislab/collideseq_reproducibility). We removed cells with high mitochondrial content (> 20% of UMIs are from mitochondrial genes) or low total mRNA counts (< 1,000 expressed genes). We then filtered nonhighly variable genes, normalized the mRNA counts within each cell, log(*x* + 1) transformed the counts, computed PCA with 50 components, computed a k‐nearest neighbor graph with k = 100, and computed UMAP embeddings and Louvain clustering on the graph. We used SoupX (Young & Behjati, 2020) for ambient RNA correction (Notebooks I‐1a, II‐1a, III‐1a on GitHub (https://github.com/theislab/collideseq_reproducibility)). We defined the cell‐cycle score as a z‐score over cells on the sum of the G2M‐phase score and S‐phase score computed with Scanpy (Wolf *et al*, 2018). We computed the fibroblast score as a z‐score defined over cells in the Louvain clusters annotated as noncycling fibroblasts on the sum of log‐normalized fibroblast marker genes per cell: Thy1, Col1a1, Postn, Vim, Prrx1, Timp3, Ccn2, Col5a1, Col1a2, Glipr2, Itgb5, Sh3kbp1, Tex264, Tnc, Cnn1, Fn1, S100a4, Twist2, Snai2, Cav1, Ecm1, Acta2, Col4a1, Col5a2, Mmp2, Mmp14, Mmp23, Col3a1, Cav2, Timp1, Timp2, Fgf7, Vcl, Itgb8.

### Cellular doublet simulation

We considered a doublet simulation between Ascl1 single‐positive cells and MyoD1 single‐positive cells to establish whether this specific doublet could explain the Ascl1 & MyoD1 double‐positive cellular state. As both source distributions are defined by cells from the respective single‐positive conditions, we sampled (*n* = 200) random pairs of simulated doublets, added their transcriptomic states (unprocessed counts), and divided these counts by two to receive simulated doublet cells in the range of total counts of the single‐positive cells. We then performed an unsupervised analysis workflow as described in (Unsupervised analysis of single‐cell RNA‐seq data) on the union of these simulated cells and the real cells under all conditions (Fig EV3E and F). To map the simulated cells into the annotated clusters defined on the real cells, we used the nearest neighbor classifier, only using the real cells with the minimal distance observed to each simulated cell.

### Differential expression analysis

The exact analysis is documented in Notebook I‐3 on GitHub (https://github.com/theislab/collideseq_reproducibility). We used Wald tests on a negative binomial generalized linear model (*GitHub ‐ theislab/diffxpy: Differential expression analysis for single‐cell RNA‐seq data*., no date) fit on cell cycle, condition, and normalization factors testing the condition effect to determine differentially expressed genes along the individual lineages. To restrict the setting to each lineage, we only considered cells from the single‐positive condition of the lineage and the control vector expressing cells for the test. *P*‐values were corrected for multiple testing using Benjamini–Hochberg correction. We declared genes as differentially expressed if they had a corrected *P*‐value of less than 0.01, a fold change of less than 2/3 or more than 3/2, and a mean expression level of at least 0.05 counts. We added the label up‐ or downregulated in multiple places for those differentially expressed genes with positive (up) and negative (down) log fold change. We inferred synergism and antagonism in a differential test on cells from the control condition, two considered single‐positive conditions, and the double‐positive condition of these two, modeling each transgene's effect individually and their interaction and tested and reported the interaction effect to yield synergism and antagonism labels: We defined synergistic effects as positive transgene interaction effects in a differential expression model if the log fold change in the double‐positive condition was positive (a gene is upregulated more strongly in the double‐positive condition than would be expected based on the sum of both transgenes in their individual single‐positive conditions). Additionally, we defined negative transgene interaction effects in a differential expression model as synergistic if the log fold change in the double‐positive condition was negative (stronger downregulation than expected). Conversely, we defined weaker upregulation and weaker downregulation as antagonistic. We defined differential expression magnitude as the L2 norm over all gene‐wise parameter estimators for an individual coefficient defined in the model, only using genes for which this coefficient was significant (FDR‐corrected *P*‐values < 0.01) and which were likely not overfitted (defined as an L1 norm of significant effects within a gene < 100).

### Gene ontology analysis

Gene ontology enrichment analysis was performed using the g:Profiler Python client (Raudvere *et al*, 2019). Differentially expressed genes (Up: log‐2‐foldchange > 2/3, Down: log‐2‐foldchange < −2/3, log‐normalized mean expression > 0.1, and adjusted *P*‐value < 0.01) for each condition were provided as input, and all expressed genes in the dataset were used as background. For the downregulated genes, gene ontology analysis was performed using the cellular component annotations whereas for upregulated genes the annotation biological process was used. The top 20 enriched GO terms were ranked based on *P*‐value and visualized using a custom graphing function.

### ChIP‐seq data analysis and synergism and antagonism annotation

The exact analysis is documented in Notebook I‐3 on GitHub (https://github.com/theislab/collideseq_reproducibility). We used published peak files for both Ascl1 and MyoD1 ChIP (Lee *et al*, 2020). We overlapped these peaks against 5′ ends of gene annotations (GRCm38), extending the annotated interval 5′ end 10 kb upstream and 2 kb downstream.

### RNA velocity and Cellrank analysis

The exact analysis for scVelo (Bergen *et al*, 2020) and CellRank (Lange *et al*, 2022) workflows are documented in code in Notebooks I‐2 and II‐2 on GitHub (https://github.com/theislab/collideseq_reproducibility). The RNA velocities mentioned in the main text are based on the dynamical RNA velocity model implemented in scVelo. Additionally, we fit the dynamical model with lenient gene filtering and a steady‐state model with standard gene filtering. We ran CellRank (Lange *et al*, 2022) on the dynamical RNA velocity model fits separately for each inferred condition using the CFLARE model on a kernel consisting of 50% transcriptomic connectivity and 50% RNA velocities. The attractors were assigned to fates by cell rank and are colored according to the annotated Louvain clusters established in the unsupervised analysis section.

### Neurogenic and myogenic scores

The exact analysis is documented in Notebook II‐4 on GitHub (https://github.com/theislab/collideseq_reproducibility). We defined the neurogenic score as *1‐diffusion pseudotime* from the most mature neurogenic cell: We defined the most mature neurogenic cell as the cell with the highest latent time assigned by scVelo during dynamical RNA velocity inference in the Ascl1 single‐positive condition. The scored cells presented are all cells in neurogenic or myogenic states excluding the cycling cluster. Similarly, we defined the myogenic score within the MyoD1 single‐positive condition. The myogenic score presented in Fig EV3G is computed as above but computed on the set excluding cells in the mature myogenic cluster to compare intermediate myogenic and neurogenic states.

### Fate titration analysis

The exact analysis is documented in Notebooks I‐2 and II‐2 on GitHub (https://github.com/theislab/collideseq_reproducibility). We scaled transgene counts into an active range between the minimum transgene count observed in the positive condition and the 99^th^ percentile of transgene counts in the single‐positive condition (maximum): $x_{\text{scaled}}=\frac{x-x_{\min}}{x_{\max}-x_{\min}}$. We visually compared two groups of cells (cell rank attractor groups or Louvain cluster groups) using 2D kernel density estimators of the joint distribution of each group over both transgenes and using the respective marginal distributions of each group. We fit a weighted logistic regression model with class‐balancing weights between both groups on the scaled transgene counts. We report the accuracy of this classifier on a held‐out test partition of 20% of the data.

### Supervised modeling

All model and analysis code is also documented in Notebook I‐4 on GitHub (https://github.com/theislab/collideseq_reproducibility) and Dataset EV1. We used linear and nonlinear models both for the classification of categorical cluster assignments and regressive prediction of the log‐normalized RNA observations of each cell. We accounted for confounding in all models: We corrected for the sample through a one‐hot encoded predictor and for the cell size through a log‐transformed size factor in the input. In all cases, the input to these models is either the one‐hot encoded condition assignment (categorical model, Fig 4F and G), the binary presence of transgenic transcription factors (binary models, Fig 4H and I), or the log transgenic transcription factor expression per cell (expression models, Fig 4F–I). The nonlinear model was a neural network of fully connected layers with two hidden layers of 64 units each and tanh activation function. We used 1e‐6 L1 and L2 penalties on linear layers. The hyperparameters of all models are also described in the supplied code. We trained all networks until convergence and evaluated performance on 10% of randomly selected test cells (Fig 4F and G) or on entirely held‐out cell sets corresponding to inferred conditions (Fig 4H and I). We trained on all cells that were not in the test set, except in Fig 4I where we tested models trained on only single‐ or single‐ and double‐positive conditions on selected triple‐positive conditions. We used the area under the curve of the receiver–operator characteristic (AUC ROC), accuracy, top‐3 accuracy, and condition class‐balanced accuracy to evaluate classification models and used R2 per cell between observed and predicted log‐normalized expression for regression models.

## Author contributions

**Bob A Hersbach:** Conceptualization; formal analysis; validation; investigation; visualization; methodology; writing – original draft; writing – review and editing. **David S Fischer:** Conceptualization; data curation; software; formal analysis; funding acquisition; validation; investigation; visualization; methodology; writing – original draft; writing – review and editing. **Giacomo Masserdotti:** Conceptualization; supervision; writing – review and editing. **Deeksha:** Validation; investigation. **Karolina Mojžišová:** Software; investigation; methodology. **Thomas Waltzhöni:** Software; formal analysis. **Diego Rodriguez‐Terrones:** Resources; data curation; software; formal analysis. **Matthias Heinig:** Resources; supervision. **Fabian J Theis:** Resources; supervision; funding acquisition; project administration; writing – review and editing. **Magdalena Götz:** Conceptualization; resources; supervision; funding acquisition; project administration; writing – review and editing. **Stefan H Stricker:** Conceptualization; resources; supervision; funding acquisition; writing – original draft; project administration; writing – review and editing.

## Disclosure and competing interests statement

FJT reports receiving consulting fees from Roche Diagnostics GmbH and Cellarity Inc., and an ownership interest in Cellarity, Inc., and Dermagnostix. The authors declare that they have no conflict of interest. FJT is an editorial advisory board member. This has no bearing on the editorial consideration of this article for publication.

## Supporting information




Appendix

Expanded View Figures PDF

Dataset EV1

Source Data for Figure 1

Source Data for Figure 5

Source Data for Expanded View

## Data Availability

Raw sequencing data as well as annotated single‐cell matrices and CUT&RUN peaks are publicly available through the NCBI Gene Expression Omnibus (GEO) under accession number GSE211864. This accession contains all sRNA‐seq data (GSE211862), scMultiome data (GSE211863) and CUT&RUN data (GSE210181) generated during this study. Furthermore, all notebooks used for data analysis are available on GitHub (https://github.com/theislab/collideseq_reproducibility) with generalizable code related to analyzing single cell reprogramming data available as a python package in Dataset EV1 (“tftools”, “fatevision”).
