# Supplementary material for: Probing cell identity hierarchies by fate titration and collision during direct reprogramming
Source: Mol Syst Biol. 2022 Sep 15;18(9):e11129. doi: 10.15252/msb.202211129 (PMC9476893; doi:10.15252/msb.202211129)
Supplement: Supplementary file 1 — Appendix [file MSB-18-e11129-s002.pdf]

Table of Contents

Appendix Figure S1: Demultiplexing & Quality control **2**

Appendix Figure S2: Gene ontology of upregulated genes **3**

Appendix Figure S3: Gene ontology on downregulated genes **4**

Appendix Figure S4: Top 40 differentially expressed genes per condition **5**

Appendix Table S1: Fibroblast score genes **6**

Appendix Table S2: List of qRT-PCR primers **7**

Appendix Table S3: List of antibodies **8**

Appendix Table S4: List of cloning primers & oligos **9**

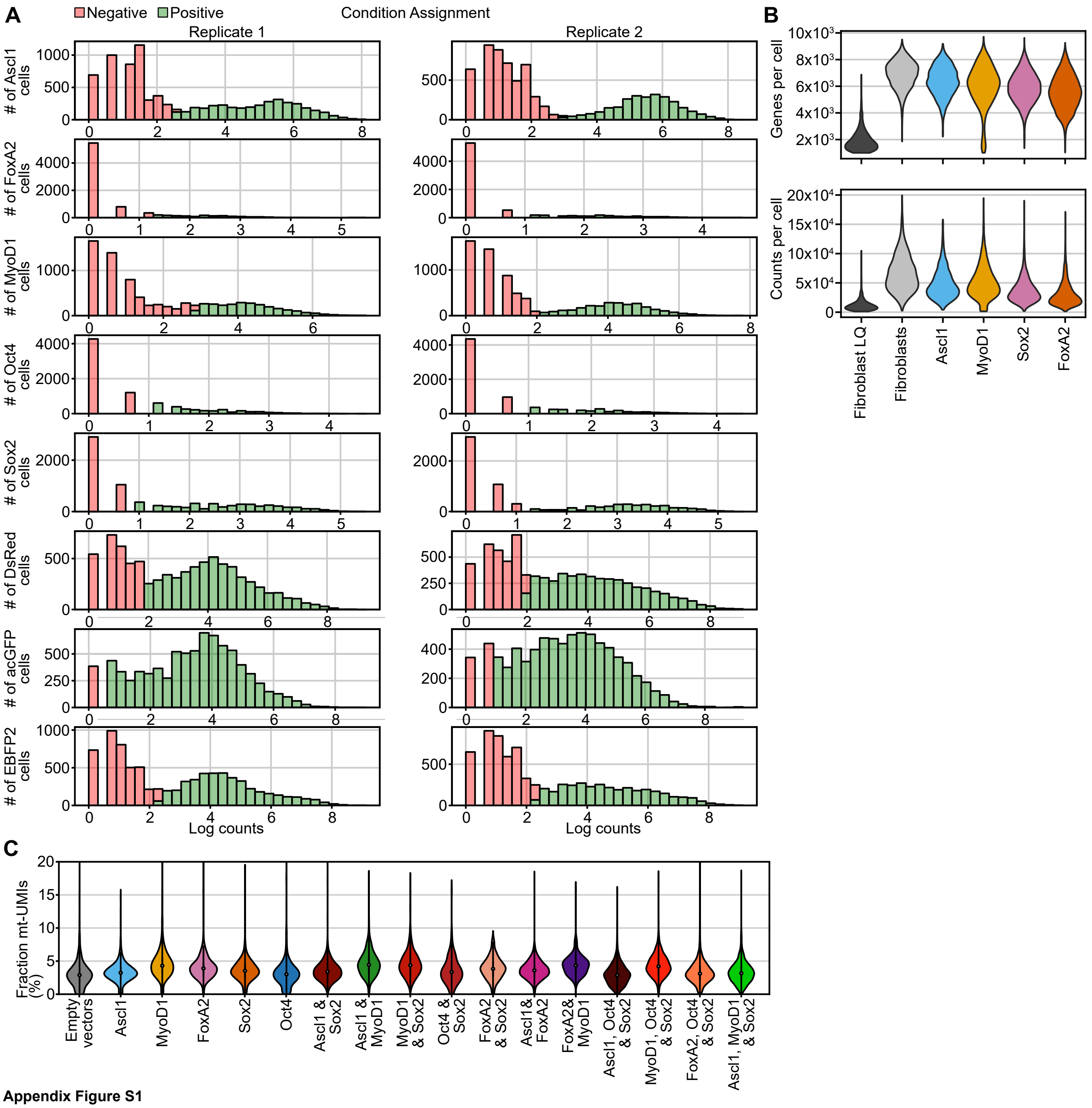

Appendix Figure S1

A. Assigned conditions in first step of algorithm for a gradient-free optimization method based on fluorophore and transgene expression. See computational demultiplexing section in Methods for further details. **B.** Average number of genes (top panel) and UMI counts (bottom panel) for indicated Louvain clusters. **C.** Fraction of mitochondrial UMIs per condition.

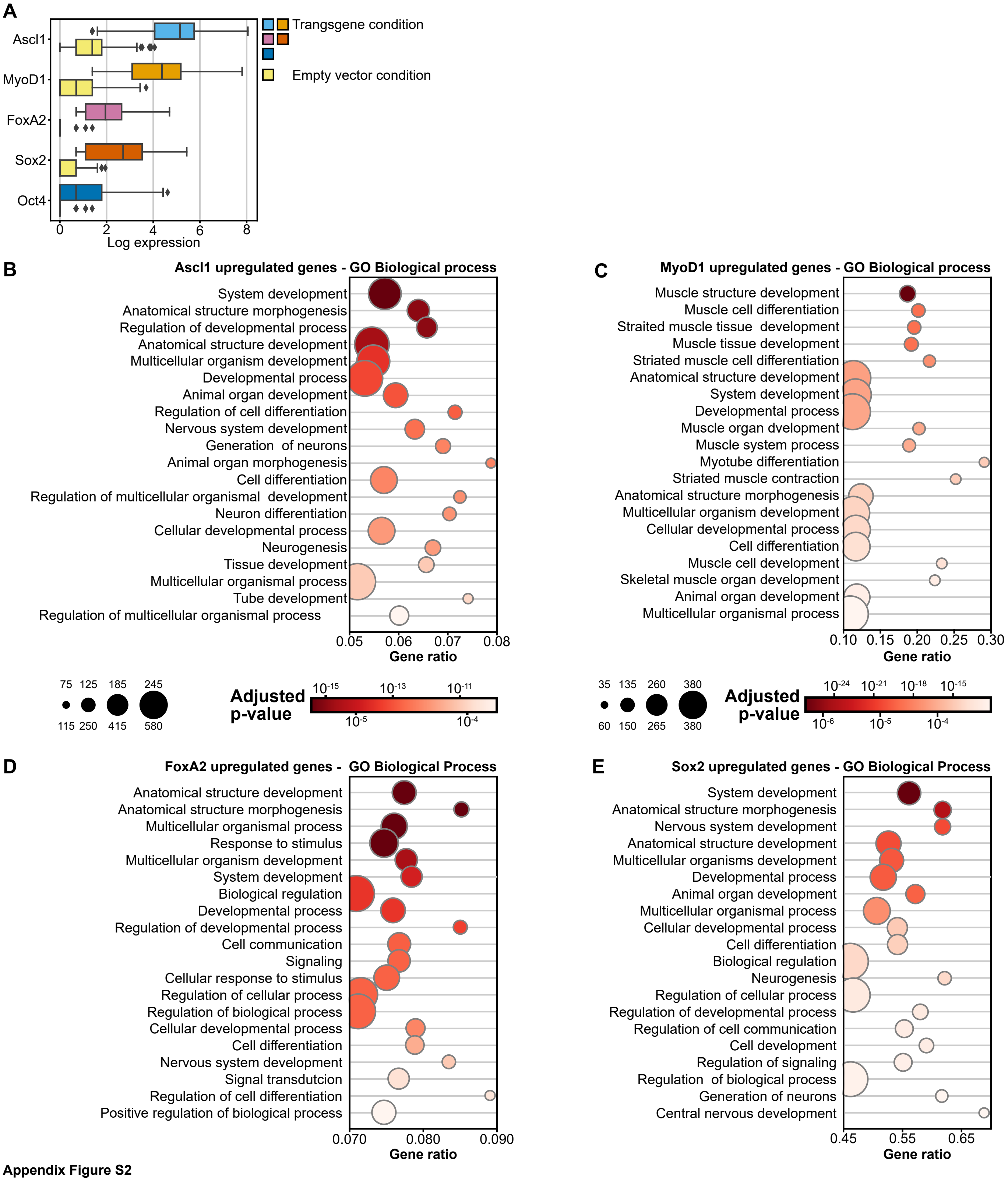

Appendix Figure S2

A. Distribution of log-normalized transcription factor expression levels in single positive conditions (Ascl1: light blue, MyoD1: orange, FoxA2: pink, Sox2: vermillion and Oct4: blue) as well as empty vector controls (yellow). **B-E.** Top 20 terms related to biological process after gene ontology (GO) enrichment analysis using G-profiler (see gene ontology analysis in Methods for further details) for genes upregulated by Ascl1 (**B**), MyoD1 (**C**), FoxA2 (**D**) and Sox2 (**E**). Gene overlap with each term is depicted as the size of the circles and color reflects the adjusted p-value. On the y-axis the gene ratio is shown.

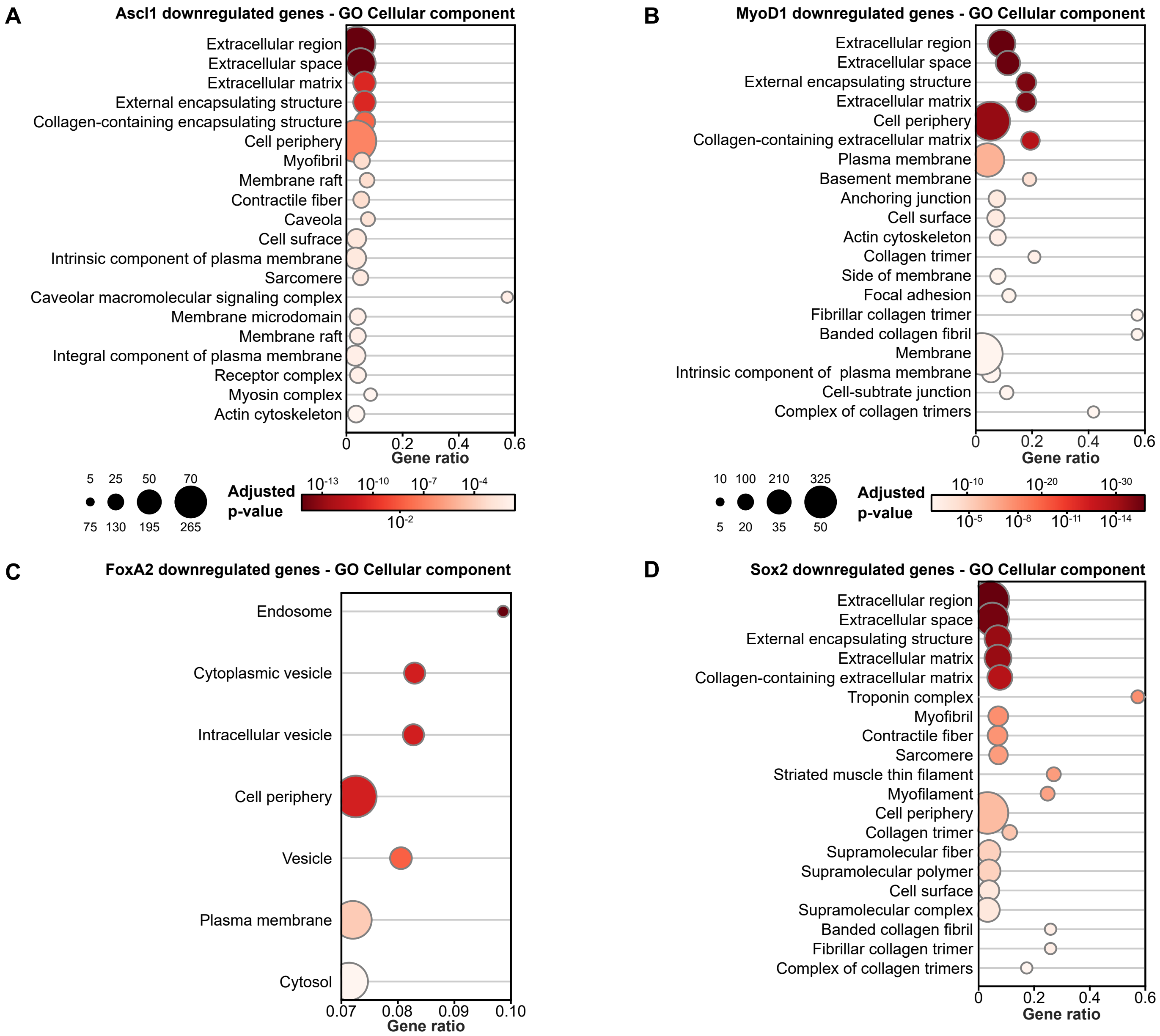

Appendix Figure S3

A-D. Top 20 terms related to cellular component after gene ontology (GO) enrichment analysis using G-profiler (see gene ontology analysis in Methods for further details) for genes downregulated by Ascl1 (**A**), MyoD1 (**B**), FoxA2 (**C**) and Sox2 (**D**). Gene overlap with each term is depicted as the size of the circles (bigger is more overlap) and color reflects the adjusted p-value. On the y-axis the gene ratio is shown.

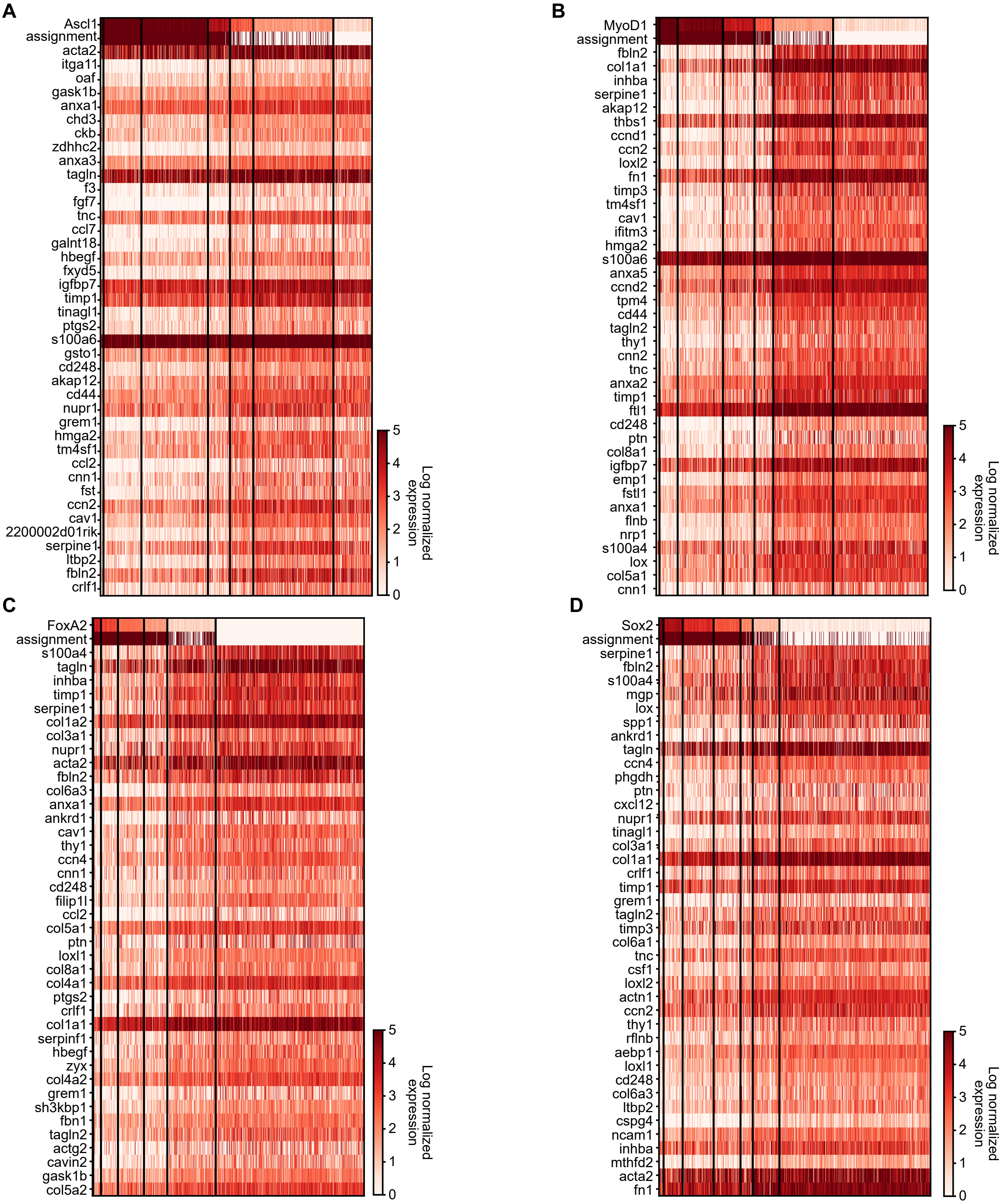

Appendix Figure S4

A-D. Log-normalized target gene expression for the top 40 most downregulated genes as compared to control fibroblasts for Ascl1 (**A**), MyoD1 (**B**), FoxA2 (**C**), Sox2 (**D**).

Appendix Table S1: Fibroblast score genes

Thy1	Ccn2	Tex264	Snai1
Col1a1	Col5a1	Tnc	Cav1
Postn	Col1a2	Cnn1	Ecm1
Vim	Glipr2	Fn1	Acta2
Prrx1	Itgb5	S100a4	Col4a1
Timp3	Sh3kbp1	Twist2	Col5a2
Mmp2	Mmp14	Mmp23	Col3a1
Cav2	Timp1	Timp2	Fgf7
Vcl	Itgb8		

Appendix Table S2: qRT-PCR primers

Target	Species	Forward primer (5' – 3')	Reverse primer (5' – 3')
Ascl1	Rat	TCG GCT ACA GCC TTC CAC	CAA AGC CCA GGT TAA CCA AC
MyoD1	Mouse	TGG CAT GAT GGA TTA CAG CGG	TCA CTG TAG TAG GCG GTG TC
Hnf1a	Mouse	GAG AAA CGC GTG GCT CTG AA	GTC CTC CTG AAG AAG TGA CTC CAC
Oct4	Mouse	AGC ACG AGT GGA AAG CAA CT	CTT TCA TGT CCT GGG ACT CCT C
FoxA2	Mouse	CGA GCA CCA TTA CGC C	GGG TAG TGC ATG ACC TG
Sox2	Mouse	CGA GCT GGT CAT GGA GTT GTA C	AAC GGC AGC TAC AGC ATG ATG C
Hes6	Mouse	ACG GAT CAA CGA GAG TCT TCA	TTC TCT AGC TTG GCC TGC AC
Dll1	Mouse	GGG ACA GAG GGG AGA AGA TG	CAC ACC CTG GCA GAC AGA T
Pvalb	Mouse	GGC AAG ATT GGG GTT GAA G	AGC AGT CAG CGC CAC TTA G
Cbfa2t3	Mouse	TGGCTACATGCCTGAAGAGAT	GCGCTTCACCTCATTCACA
GAPDH	Mouse	TTG CAG TGG CAA AGT GGA GA	CGT TGA ATT TGC CGT GAG TG
GAPDH	Mouse	GTG TTC CTA CCC CCA ATG TGT	ATT GTC ATA CCA GGA AAT GAG CTT

Appendix Table S3: Antibodies

Primary Antibody	Host species	Isotype	Dilution	Source
RFP	Rabbit		1:1000	RRID: AB_2209751<br>Rockland (600-401-379)
GFP	Chicken	-	1:1000	RRID:AB_10000240<br>Aves Labs (GFP-1020)
Ascl1	Mouse	IgG1	1:400	RRID:AB_396479<br>BD Pharmingen (556604)
MyoD1	Mouse	IgG1	1:500	RRID:AB_395255<br>BD Pharmingen (554130)
Hnf1a	Rabbit	-	1:400	RRID:AB_2728751<br>Cell Signaling Technology (89670)
Oct4	Rabbit	-	1:250	RRID:AB_2687916<br>Abcam (181557)
Sox2	Mouse	IgG1	1:400	RRID:AB_10710406<br>Abcam (79351)
FoxA2	Rabbit		1:400	RRID:AB_10891055<br>Cell Signaling Technology (8186S)
FLAG	Mouse	IgG1	1:200	RRID:AB_262044<br>Sigma-Aldrich (F1804)
**Secondary antibody**				
DAPI	Goat		1:1000	Sigma-Aldrich (MBD0015)
Anti-chicken 488	Goat		1:1000	RRID:AB_2534096<br>ThermoFisher A11039
Anti-rabbit 546	Goat		1:1000	RRID:AB_2534077<br>ThermoFisher A11010
Anti-mouse IgG1 594	Goat		1:1000	RRID:AB_2535767<br>ThermoFisher A21125

Appendix Table S4: Cloning primers & Oligos

Primer name	Primer sequence (5' – 3')
TetOn Fwd	GGC GCA GTA GTC CAA ACA GGG ACA GCA GAG ATC
TetOn Rev	TAG GCA GCC TGC ACC TGA GGA GCT CGA GAG GTC AGG TCA AAA CAG CGT GGA TG
Colors Fwd	ACG CTG TTT TGA CCT CAC TAG TCG TTA CAT AAC TTA CGG TAA
acGFP Rev	CTG CAC CTG AGG AGC TTA CTT GTA CAG CTC ATC C
EBFP2 Rev	CTG CAC CTG AGG AGC TTA CTT GTA CAG CTC GTC C
CMV Fwd	CTG TTT TGA CCT GAC CTC ACT AGT CGT TAC ATA ACT TAC GGT AAA TG
CMV Rev	ATG GTG GCG AGT CCG GTA GCG CTA GC
DsRed Fwd	CCG GAC TCG CCA CCA TGG CCT CCT CC
DsRed Rev	CTG CAC CTG AGG AGC CTA CAG GAA CAG GTG GTG GCG
SV40 Fwd (Red)	CTG TTT TGA CCT GAC CTC ACT GAA ACA TAA AAT GAA TGC AAT TG
SV40 (Green & Blue)	CGC TGT TTT GAC CTC ACT GAA ACA TAA AAT GAA TGC AAT TG
SV40 Rev	CCG TAA GTT ATG TAA CGA TAA GAT ACA TTG ATG AGT TTG G
Ascl1 Fwd	TGA AAC ATA AAA TGA ATG CCT AAG CCA CCA TGG AGA GCT CTG GCA AGA TG
Ascl1 Rev	TAA ACA AGT TAA CAA CAA CTC AGA ACC AGT TGG TAA AG
MyoD1 Fwd	TGA AAC ATA AAA TGA ATG CGC CAC CAT GGA GCT TCT ATC GCC G
MyoD1 Rev	TAA ACA AGT TAA CAA CAA CAT CTC TCA AAG CAC CTG ATA AAT C
Hnf1a Fwd	TGA AAC ATA AAA TGA ATG CGC CAC CAT GGT TTC TAA GCT GAG CC
Hnf1a Rev	TAA ACA AGT TAA CAA CAA CGG CAC TTA CTG GGA AGA GGA GGC
Oct4 Fwd	TGA AAC ATA AAA TGA ATG CGC CAC CAT GGC TGG ACA CCT GGC TTC
Oct4 Rev	TAA ACA AGT TAA CAA CAA CGT GCC TCA GTT TGA ATG CAT GGG

AU Fwd	ATG TAA GTC GAG TTG TTG TT
AU Rev	TAA GCT GCA ATA AAC AAG TT
MyoD1 FLAG Fwd	CGA TGA CAA GAT GGA GCT TCT ATC GCC G
MyoD1 FLAG Rev	CGA TGA CAA GAT GGA GCT TCT ATC GCC G
FLAG Fwd	CTG AAA CAT AAA ATG AAT GCT GCT GGG CCA CCA TGG ACT
FLAG Rev	GAA GCT CCAT CTT GTC ATC GTC ATC CTT GTA ATC G
