# Supplementary material for: Probing cell identity hierarchies by fate titration and collision during direct reprogramming
Source: Mol Syst Biol. 2022 Sep 15;18(9):e11129. doi: 10.15252/msb.202211129 (PMC9476893; doi:10.15252/msb.202211129)
Supplement: Supplementary file 2 — Expanded View Figures PDF [file MSB-18-e11129-s003.pdf]

Expanded View Figures

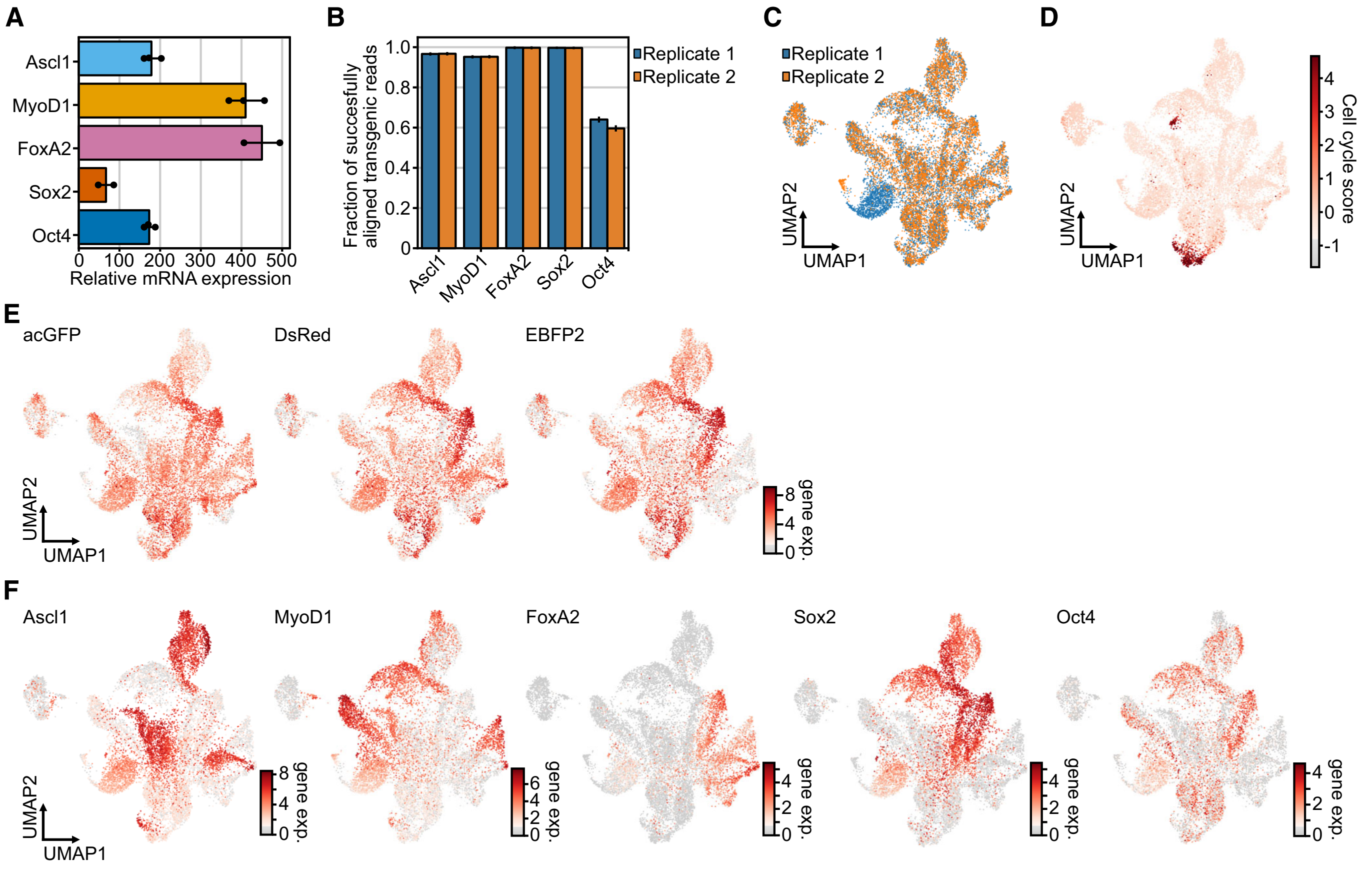

Figure EV1. Analysis of transgene induction and detection.

- A Quantification of transgene induction using qRT–PCR. Shown is the mean fold change of doxycycline-induced samples over noninduced samples for the indicated transcription factors after 48 h (n = 2/3). Error bars represent 95% confidence interval. Primers used for qPCR are listed in Appendix Table S2.
- B Fraction of UMIs mapped to transgenic allele within cells expressing a single reprogramming factor. Reads for transgenic transcripts were distinguished from endogenous transcripts using a custom annotation based on single nucleotide polymorphisms (See Generation of a modified gene annotation section in Materials and Methods for further details). Shown are 15,768 cells per barplot with the 95% confidence interval as error bars.
- C Uniform Manifold Approximation and Projection (UMAP) embedding of scRNA-seq data colored by technical replicate (Replicate 1: blue, Replicate 2: orange) after ambient RNA correction.
- D Cell-cycle score superimposed on UMAP embedding of ambient corrected data (See Unsupervised analysis of single-cell RNA-seq data in Materials and Methods).
- E Log fluorophore expression superimposed on a UMAP embedding after ambient RNA correction. Shown are fluorophore expression levels (gene exp.) on a logarithmic (ln) scale.
- F Log transgene expression superimposed on a UMAP embedding after ambient RNA correction. Shown are transgene expression (gene exp.) levels on a logarithmic (ln) scale.

Source data are available online for this figure.

© 2022 The Authors

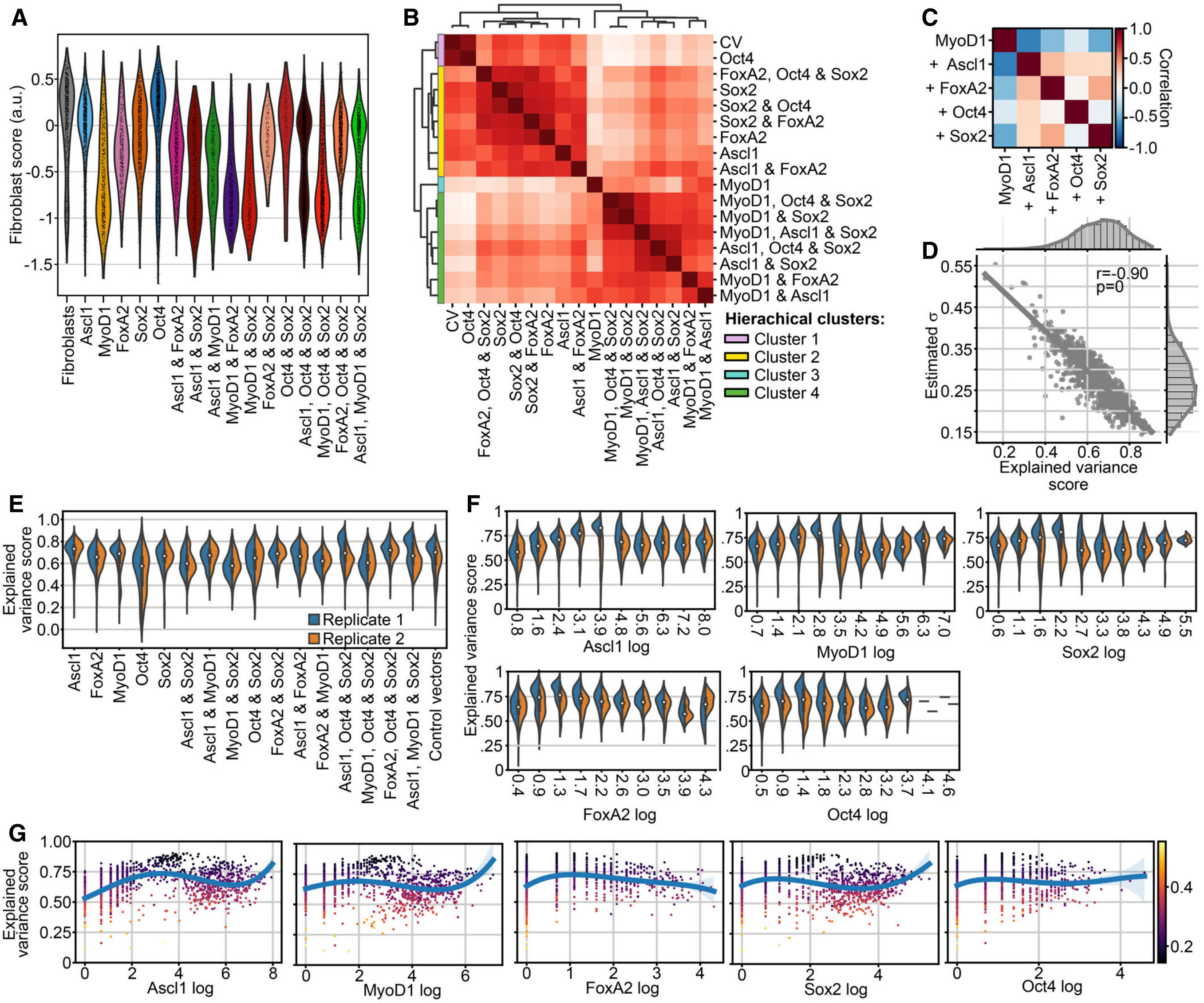

Figure EV2. Transcriptomic effects of factor collision and general determinism in Collide-seq data.

A Fibroblast score (see Unsupervised analysis of single-cell RNA-seq data section in Materials and Methods and Appendix Table S1 for further details) for indicated conditions. The number of data points per violin plot is the number of cells per matched condition shown in Fig 2A.

B Similarity of pseudobulk (mean expression per condition) principal components per condition clustered hierarchically. Colors of clusters correspond to those used in Fig 4B.

C Correlation matrix of linear model effects for correlation between individual factors and collision state (see Differential expression analysis section in Materials and Methods for further details). Color defines direction of correlation (negative = blue, positive = red), and color tone depicts size of correlation (darker = higher).

D Correlation of predictive accuracy, measured as explained variance, on held-out test data with the predicted uncertainty of the model, measured as the mean of the predicted standard deviation by gene.

E Explained variance of model prediction by condition and replicate. For each violin, the center dot represents the median, the centerline defines the range and the solid box marks the interquartile range (IQR).

F, G Explained variance of cells binned by individual transgene expression (F) or individually with trend fit (G). In (G), the color indicates the predicted uncertainty measured as the mean standard deviation over output genes. For each violin in F, the center dot represents the median, the centerline defines the range and the solid box marks the interquartile range (IQR).

© 2022 The Authors

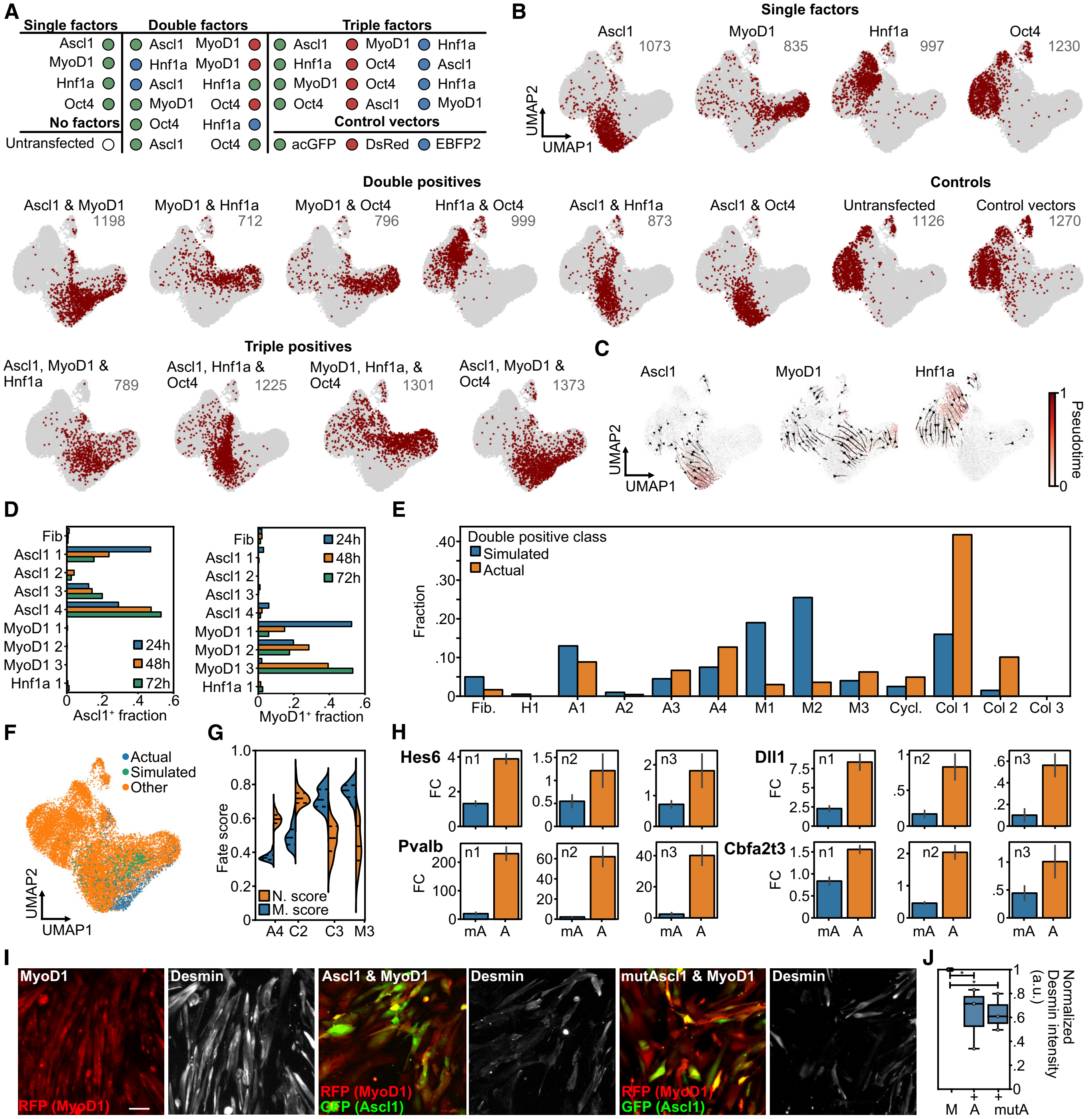

Figure EV3.

© 2022 The Authors

Figure EV3. Investigation of Ascl1 and MyoD1 collision at three defined time points.

A Schematic overview of experimental conditions in second Collide-seq experiment.

B Visualization of assignment outcome (see Computational demultiplexing section in Materials and Methods for further details) for individual cells superimposed on a Uniform Manifold Approximation and Projection (UMAP) embedding of second Collide-seq dataset. Depicted are cells positive for the indicated conditions and their total number.

C Condition-based RNA velocity trajectories. Shown are RNA velocities computed within single positive cells, projected on a UMAP embedding and computed on the transcriptomes of all cells (see RNA velocity and CellRank section in Materials and Methods for further details). Superimposed is the latent pseudotime (lineage progression coordinate) computed with scVelo (Bergen *et al*, 2020) in the context of the velocity computation.

D Relative abundance of cells from Ascl1 (left) and MyoD1 (right) single-positive conditions within Louvain clusters (*x*-axis) colored by the time point of their collection.

E Fraction of cells per Louvain cluster colored according to whether they were actual or *in silico* simulated double-positive cells.

F Simulated double positives and actual double positives colored and superimposed on UMAP.

G Neuronal (N.score) and myogenic (M.score) score for Ascl1 and MyoD1 double-positive cells residing in collision states.

H Comparison of target gene induction by Ascl1 and mutAscl1 using qRT–PCR. Shown is the fold change of Ascl1 and mutAscl1 induced gene expression for the indicated genes as compared to untransfected fibroblasts 48 h after induction. Each panel represents a biological replicate (*n*1-3). Primers used for qRT–PCR are listed in Appendix Table S2. Error bars represent standard deviation for technical replicates within each biological replicate. $n = 3$ biological replicates.

I Representative images of Desmin fluorescence intensity after 72 h of transgene induction for the indicated conditions. Scale bars represent 100 μm. mutAscl1 = mutant Ascl1.

J Normalized Desmin fluorescence intensity for indicated conditions (see Fluorescence intensity quantification section in Materials and Methods for further details). For each box, the centerline defines the median, the height of the box is given by the interquartile range (IQR), the whiskers are given by 1.5 * IQR, and the outliers are given as points beyond the minimum or maximum whisker. Pairwise comparisons were performed with the Mann–Whitney *U* test and correction for multiple testing performed with Benjamini–Hochberg correction. $^*P < 0.05$ (MyoD1 vs. Ascl1 and MyoD1: $P = 0.048$, MyoD1 vs. mutAscl1 and MyoD1: $P = 0.048$, Ascl1 and MyoD1 vs. mutAscl1 and MyoD1 = 0.5). $n = 3$ biological replicates.

Source data are available online for this figure.

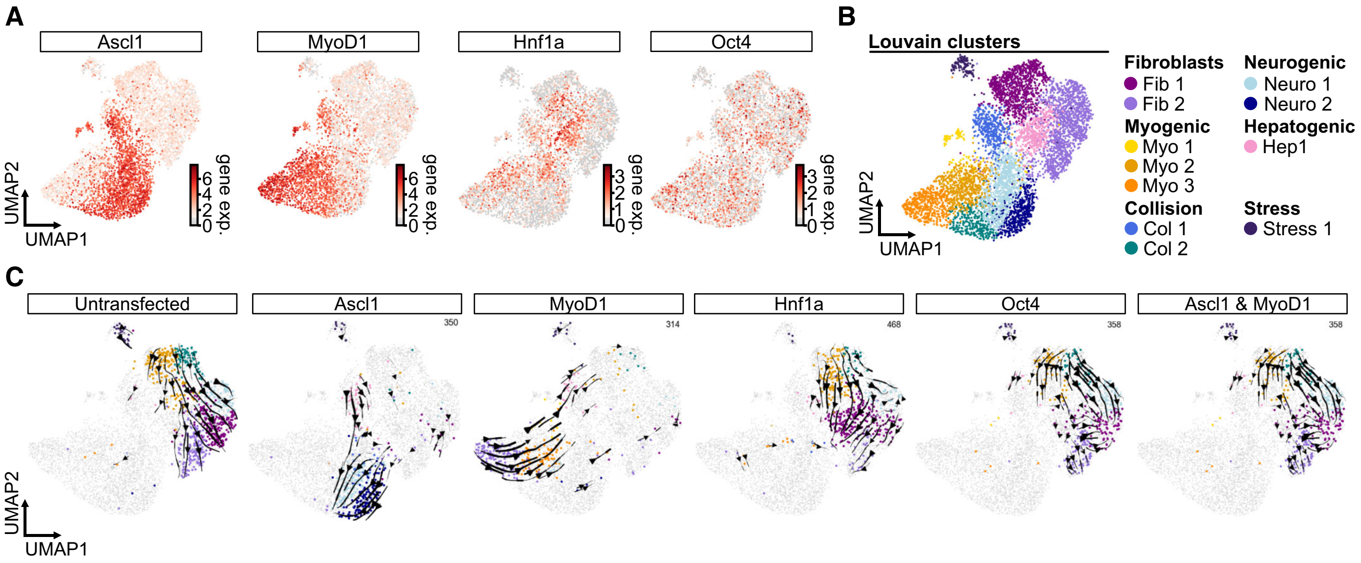

Figure EV4. Validation of collision effects in a biological replicate.

A Log-normalized transgene expression superimposed on Uniform Manifold Approximation and Projection (UMAP) embedding of separate 48 h dataset. Shown are total count-normalized transgene expression levels (gene exp.) on a logarithmic (ln) scale.

B Louvain clustering superimposed on UMAP embedding.

C Condition-based RNA velocity on a UMAP computed on cells from designated conditions. Shown is a UMAP of all cells in the dataset (gray). Superimposed are cells from the indicated condition colored in with the terminal fate assigned by CellRank (Lange *et al*, 2022), see RNA velocity and CellRank section in Materials and Methods for further details).

© 2022 The Authors

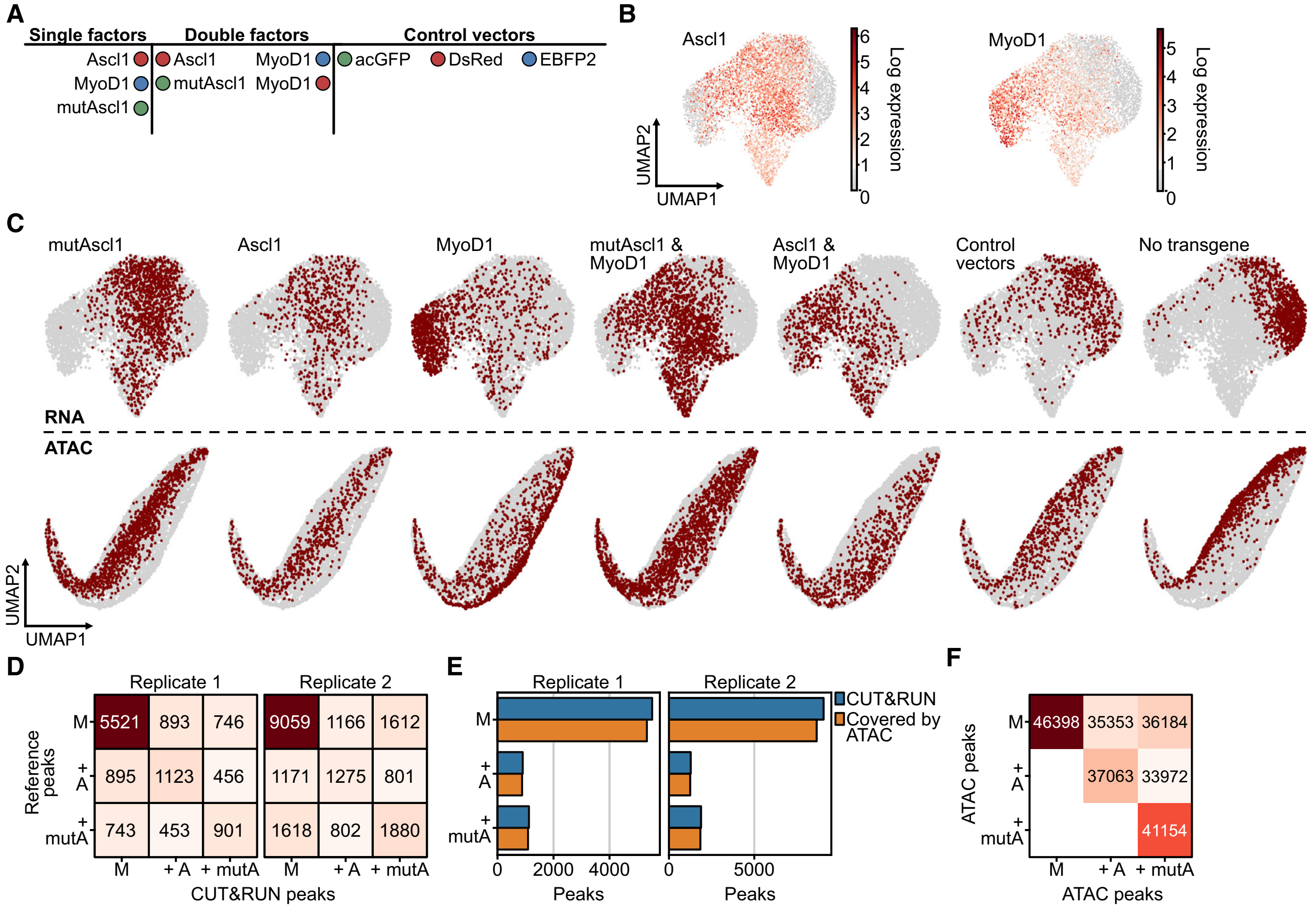

Figure EV5. Multimodal analysis of Ascl1 and MyoD1 collision.

A Schematic overview of experimental conditions for third Collide-seq experiment.

B Log transgene expression superimposed on a Uniform Manifold Approximation and Projection (UMAP) embedding for Ascl1 (left panel) and MyoD1 (right panel). Shown are transgene expression levels on a logarithmic (ln) scale.

C Visualization of assignment outcome (see Computational demultiplexing section in Materials and Methods for further details) for individual cells superimposed on scRNA-seq UMAP embedding (top panels) and scATAC-seq UMAP embedding (bottom panels). Depicted are cells positive for the indicated conditions.

D Number of CUT&RUN peaks from a condition on the *y*-axis overlapping with CUT&RUN peaks from a condition on the *x*-axis. The presented number of overlapping peaks is presented for both CUT&RUN replicates shown in Fig 6G.

E Bar plot showing the number of CUT&RUN peaks covered by scATAC-seq for two biological replicates.

F Confusion matrix showing the number of ATAC peaks in the different experimental conditions.

© 2022 The Authors
