# Supplementary figures and images for: Probing cell identity hierarchies by fate titration and collision during direct reprogramming
Source: Mol Syst Biol. 2022 Sep 15;18(9):e11129. doi: 10.15252/msb.202211129 (PMC9476893; doi:10.15252/msb.202211129)

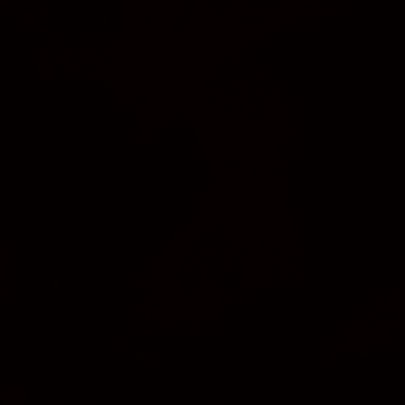

Supplement: Supplementary file 4 — Source Data for Figure 1 [file MSB-18-e11129-s006.zip › SD_Figure_1/1D/Processed/Sox2 - dox/TF.jpg]

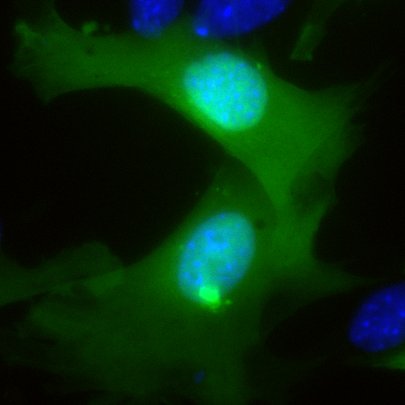

Supplement: Supplementary file 4 — Source Data for Figure 1 [file MSB-18-e11129-s006.zip › SD_Figure_1/1D/Processed/Sox2 - dox/Composite (RGB).jpg]

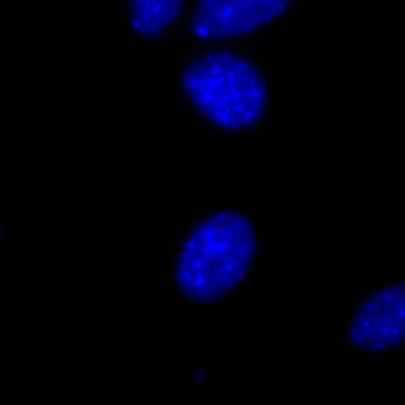

Supplement: Supplementary file 4 — Source Data for Figure 1 [file MSB-18-e11129-s006.zip › SD_Figure_1/1D/Processed/Sox2 - dox/DAPI.jpg]

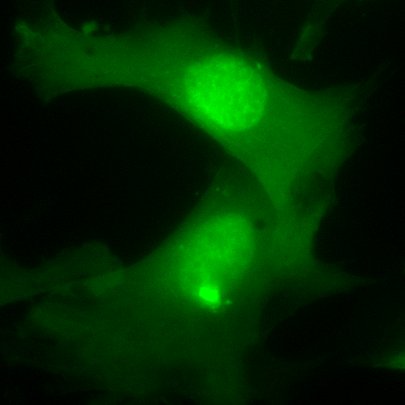

Supplement: Supplementary file 4 — Source Data for Figure 1 [file MSB-18-e11129-s006.zip › SD_Figure_1/1D/Processed/Sox2 - dox/GFP.jpg]

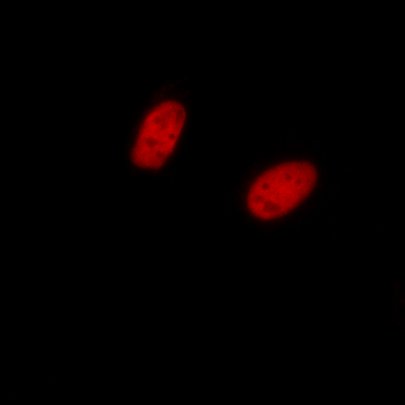

Supplement: Supplementary file 4 — Source Data for Figure 1 [file MSB-18-e11129-s006.zip › SD_Figure_1/1D/Processed/Sox2 + dox/TF.jpg]

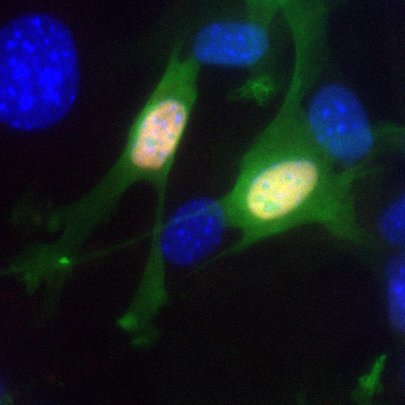

Supplement: Supplementary file 4 — Source Data for Figure 1 [file MSB-18-e11129-s006.zip › SD_Figure_1/1D/Processed/Sox2 + dox/Composite (RGB).jpg]

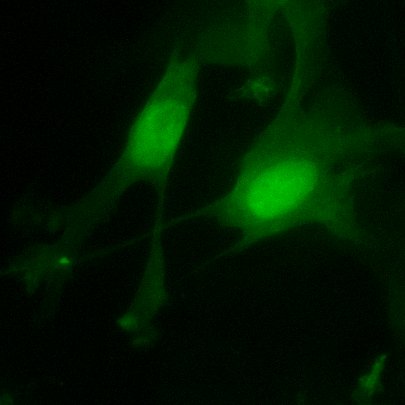

Supplement: Supplementary file 4 — Source Data for Figure 1 [file MSB-18-e11129-s006.zip › SD_Figure_1/1D/Processed/Sox2 + dox/C1-Sox2 dox paper.jpg]

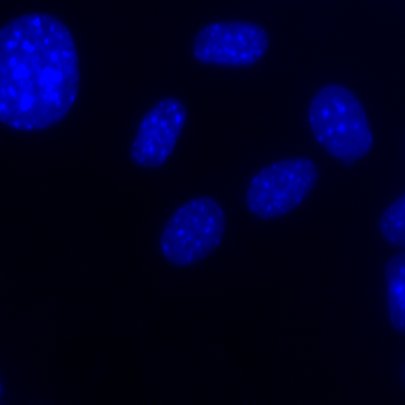

Supplement: Supplementary file 4 — Source Data for Figure 1 [file MSB-18-e11129-s006.zip › SD_Figure_1/1D/Processed/Sox2 + dox/DAPI.jpg]

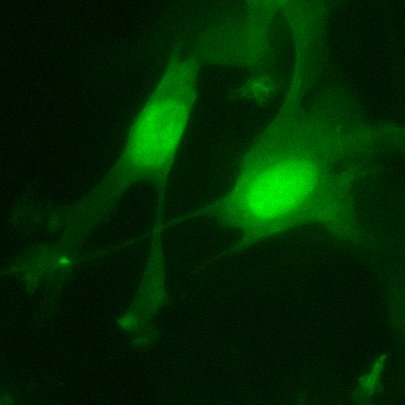

Supplement: Supplementary file 4 — Source Data for Figure 1 [file MSB-18-e11129-s006.zip › SD_Figure_1/1D/Processed/Sox2 + dox/GFP.jpg]

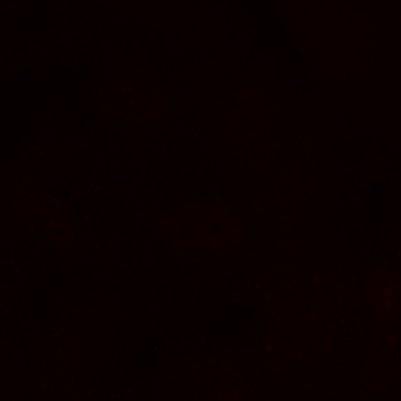

Supplement: Supplementary file 4 — Source Data for Figure 1 [file MSB-18-e11129-s006.zip › SD_Figure_1/1D/Processed/Ascl1 - dox/TF.jpg]

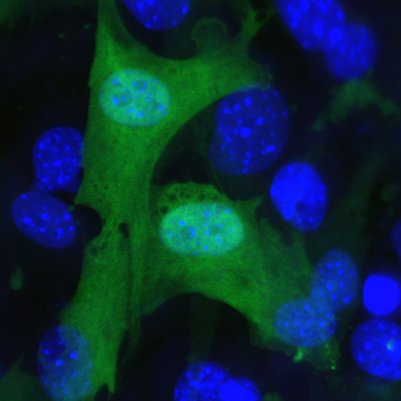

Supplement: Supplementary file 4 — Source Data for Figure 1 [file MSB-18-e11129-s006.zip › SD_Figure_1/1D/Processed/Ascl1 - dox/Composite (RGB).jpg]

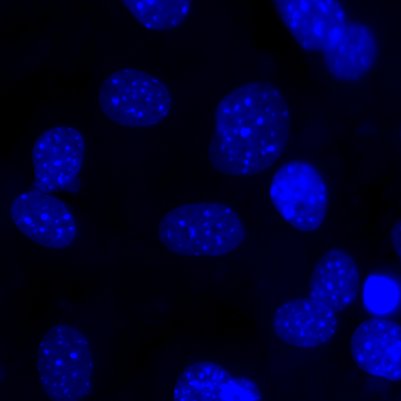

Supplement: Supplementary file 4 — Source Data for Figure 1 [file MSB-18-e11129-s006.zip › SD_Figure_1/1D/Processed/Ascl1 - dox/DAPI.jpg]

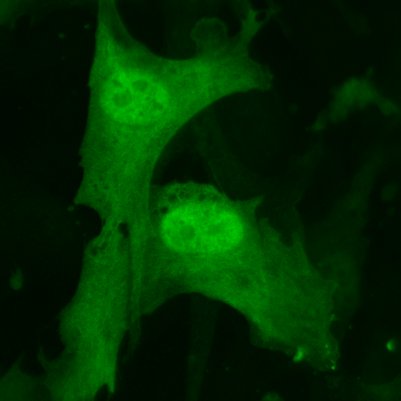

Supplement: Supplementary file 4 — Source Data for Figure 1 [file MSB-18-e11129-s006.zip › SD_Figure_1/1D/Processed/Ascl1 - dox/GFP.jpg]

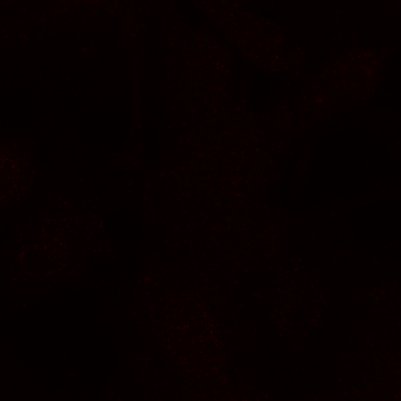

Supplement: Supplementary file 4 — Source Data for Figure 1 [file MSB-18-e11129-s006.zip › SD_Figure_1/1D/Processed/Oct4 - dox/TF.jpg]

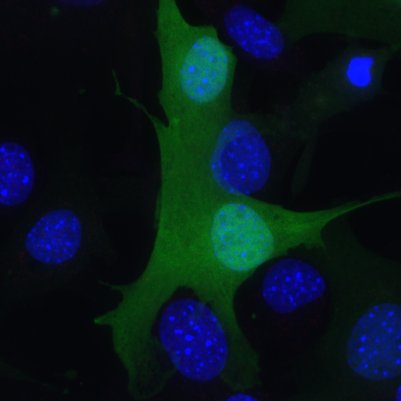

Supplement: Supplementary file 4 — Source Data for Figure 1 [file MSB-18-e11129-s006.zip › SD_Figure_1/1D/Processed/Oct4 - dox/Composite (RGB).jpg]

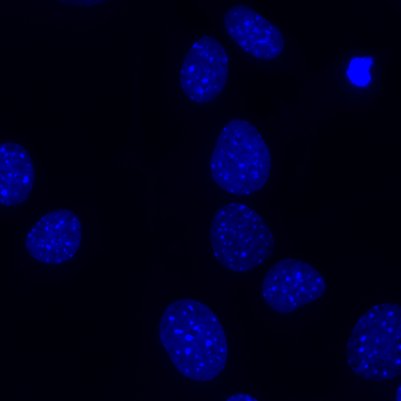

Supplement: Supplementary file 4 — Source Data for Figure 1 [file MSB-18-e11129-s006.zip › SD_Figure_1/1D/Processed/Oct4 - dox/DAPI.jpg]

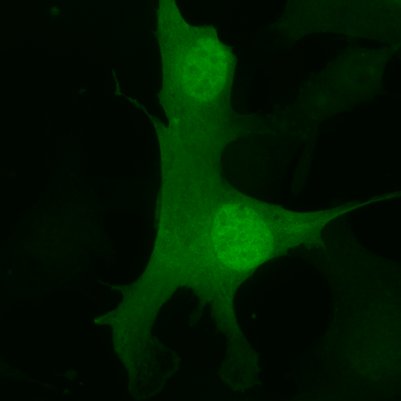

Supplement: Supplementary file 4 — Source Data for Figure 1 [file MSB-18-e11129-s006.zip › SD_Figure_1/1D/Processed/Oct4 - dox/GFP.jpg]

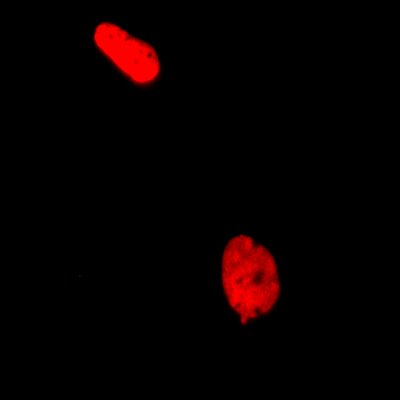

Supplement: Supplementary file 4 — Source Data for Figure 1 [file MSB-18-e11129-s006.zip › SD_Figure_1/1D/Processed/Ascl1 + dox/TF.jpg]

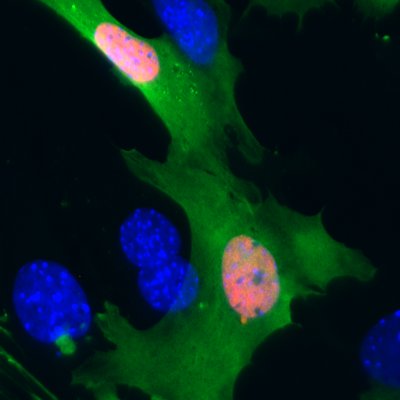

Supplement: Supplementary file 4 — Source Data for Figure 1 [file MSB-18-e11129-s006.zip › SD_Figure_1/1D/Processed/Ascl1 + dox/Composite (RGB).jpg]

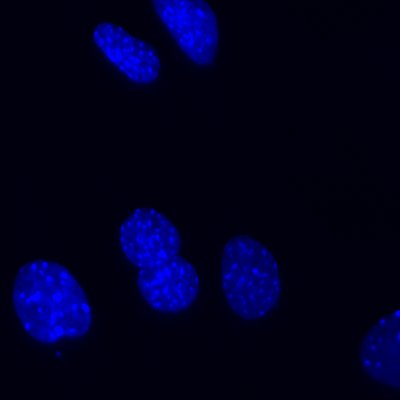

Supplement: Supplementary file 4 — Source Data for Figure 1 [file MSB-18-e11129-s006.zip › SD_Figure_1/1D/Processed/Ascl1 + dox/DAPI.jpg]

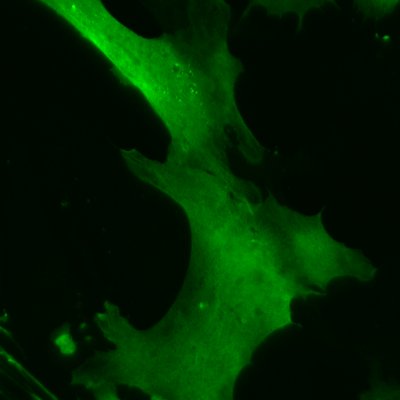

Supplement: Supplementary file 4 — Source Data for Figure 1 [file MSB-18-e11129-s006.zip › SD_Figure_1/1D/Processed/Ascl1 + dox/GFP.jpg]

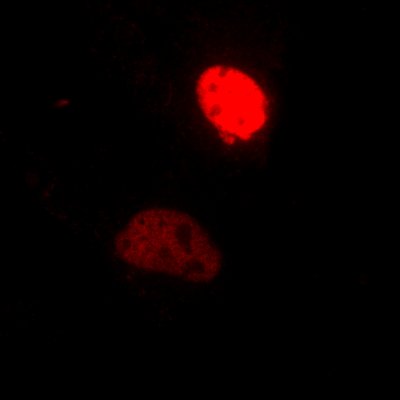

Supplement: Supplementary file 4 — Source Data for Figure 1 [file MSB-18-e11129-s006.zip › SD_Figure_1/1D/Processed/Oct4 + dox/TF.jpg]

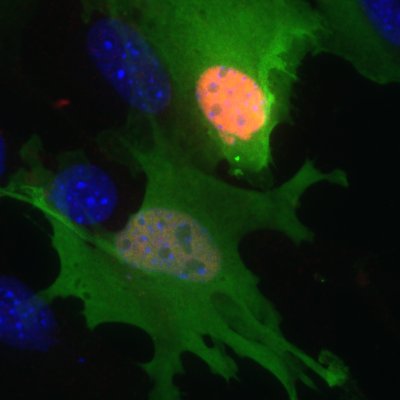

Supplement: Supplementary file 4 — Source Data for Figure 1 [file MSB-18-e11129-s006.zip › SD_Figure_1/1D/Processed/Oct4 + dox/Composite (RGB).jpg]

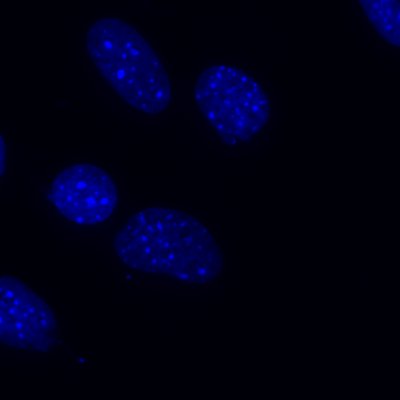

Supplement: Supplementary file 4 — Source Data for Figure 1 [file MSB-18-e11129-s006.zip › SD_Figure_1/1D/Processed/Oct4 + dox/DAPI.jpg]

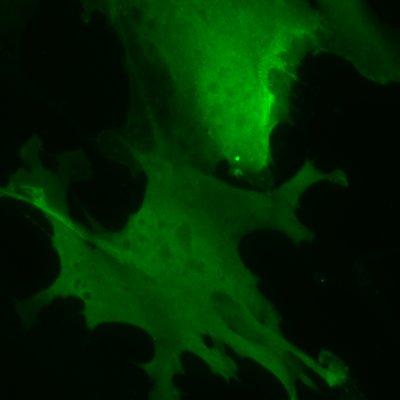

Supplement: Supplementary file 4 — Source Data for Figure 1 [file MSB-18-e11129-s006.zip › SD_Figure_1/1D/Processed/Oct4 + dox/GFP.jpg]

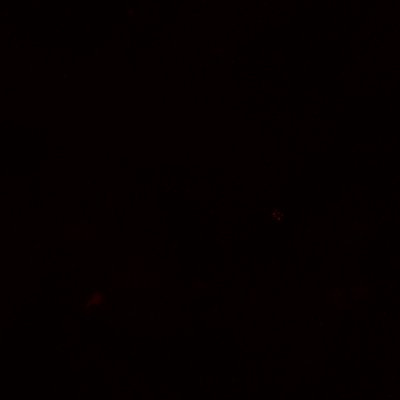

Supplement: Supplementary file 4 — Source Data for Figure 1 [file MSB-18-e11129-s006.zip › SD_Figure_1/1D/Processed/MyoD1 - dox/TF.jpg]

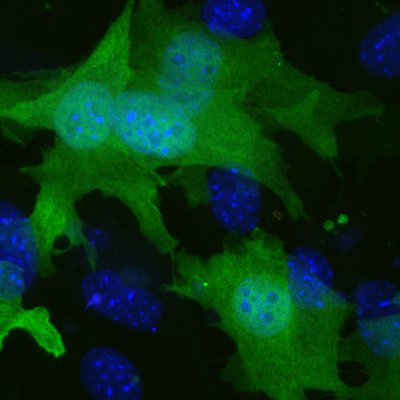

Supplement: Supplementary file 4 — Source Data for Figure 1 [file MSB-18-e11129-s006.zip › SD_Figure_1/1D/Processed/MyoD1 - dox/Composite (RGB).jpg]

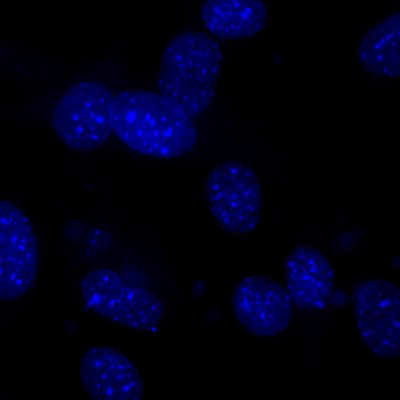

Supplement: Supplementary file 4 — Source Data for Figure 1 [file MSB-18-e11129-s006.zip › SD_Figure_1/1D/Processed/MyoD1 - dox/DAPI.jpg]

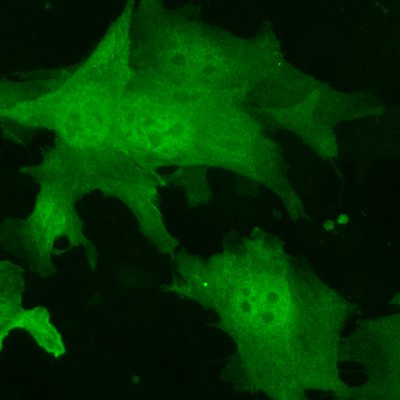

Supplement: Supplementary file 4 — Source Data for Figure 1 [file MSB-18-e11129-s006.zip › SD_Figure_1/1D/Processed/MyoD1 - dox/GFP.jpg]

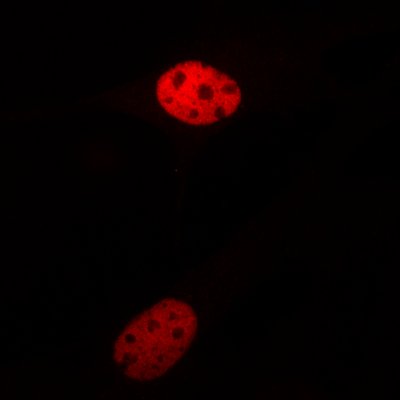

Supplement: Supplementary file 4 — Source Data for Figure 1 [file MSB-18-e11129-s006.zip › SD_Figure_1/1D/Processed/MyoD1 + dox/TF.jpg]

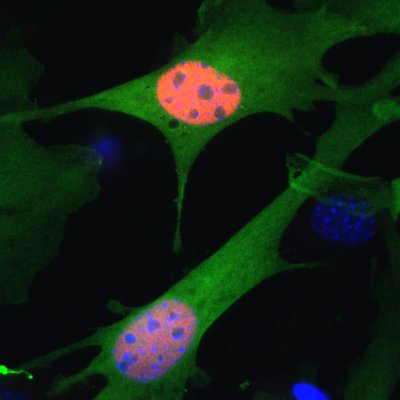

Supplement: Supplementary file 4 — Source Data for Figure 1 [file MSB-18-e11129-s006.zip › SD_Figure_1/1D/Processed/MyoD1 + dox/Composite (RGB).jpg]

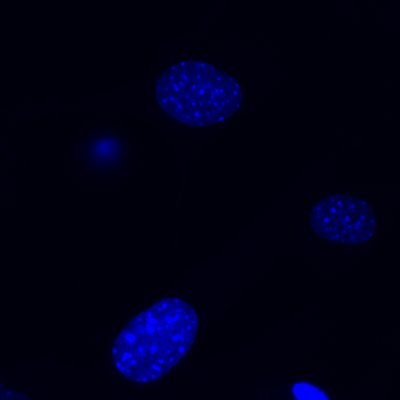

Supplement: Supplementary file 4 — Source Data for Figure 1 [file MSB-18-e11129-s006.zip › SD_Figure_1/1D/Processed/MyoD1 + dox/DAPI.jpg]

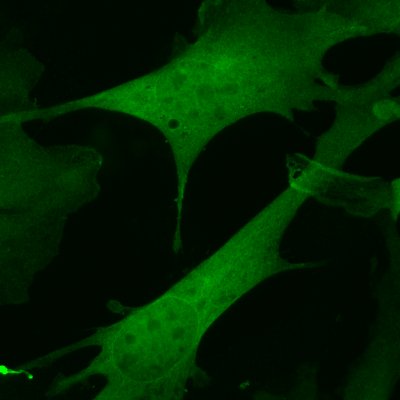

Supplement: Supplementary file 4 — Source Data for Figure 1 [file MSB-18-e11129-s006.zip › SD_Figure_1/1D/Processed/MyoD1 + dox/GFP.jpg]

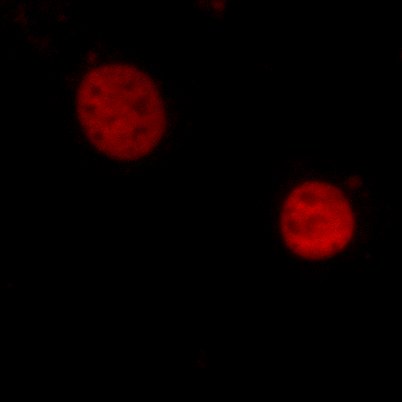

Supplement: Supplementary file 4 — Source Data for Figure 1 [file MSB-18-e11129-s006.zip › SD_Figure_1/1D/Processed/FoxA2 + dox/TF.jpg]

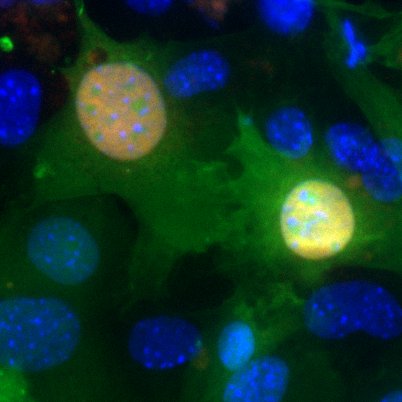

Supplement: Supplementary file 4 — Source Data for Figure 1 [file MSB-18-e11129-s006.zip › SD_Figure_1/1D/Processed/FoxA2 + dox/Composite (RGB).jpg]

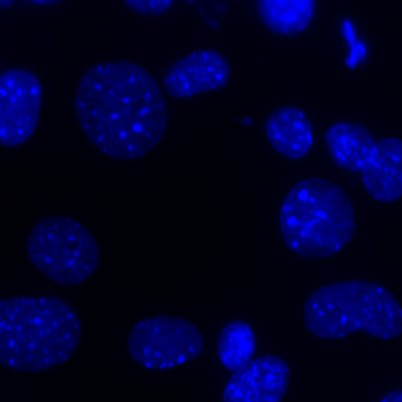

Supplement: Supplementary file 4 — Source Data for Figure 1 [file MSB-18-e11129-s006.zip › SD_Figure_1/1D/Processed/FoxA2 + dox/DAPI.jpg]

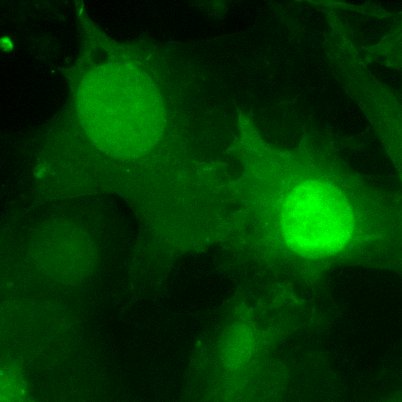

Supplement: Supplementary file 4 — Source Data for Figure 1 [file MSB-18-e11129-s006.zip › SD_Figure_1/1D/Processed/FoxA2 + dox/GFP.jpg]

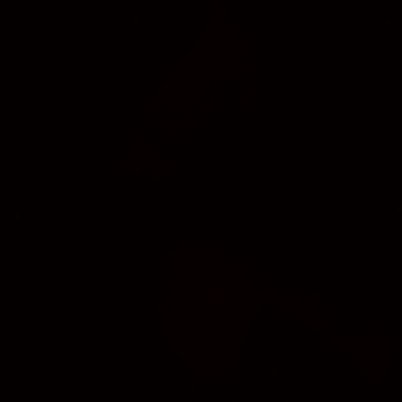

Supplement: Supplementary file 4 — Source Data for Figure 1 [file MSB-18-e11129-s006.zip › SD_Figure_1/1D/Processed/FoxA2 - dox/TF.jpg]

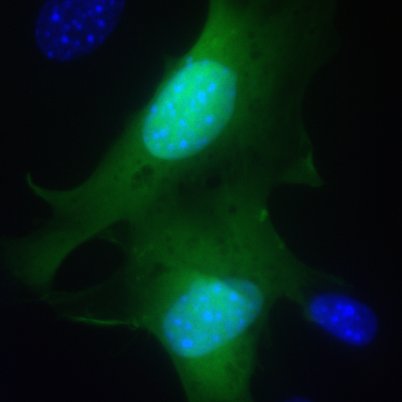

Supplement: Supplementary file 4 — Source Data for Figure 1 [file MSB-18-e11129-s006.zip › SD_Figure_1/1D/Processed/FoxA2 - dox/Composite (RGB).jpg]

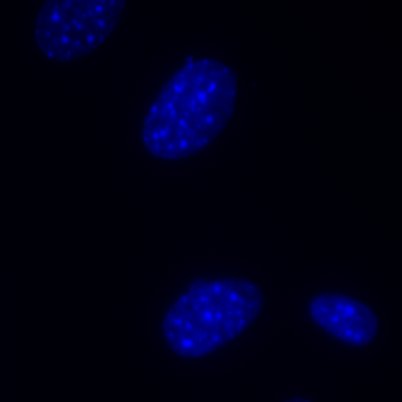

Supplement: Supplementary file 4 — Source Data for Figure 1 [file MSB-18-e11129-s006.zip › SD_Figure_1/1D/Processed/FoxA2 - dox/DAPI.jpg]

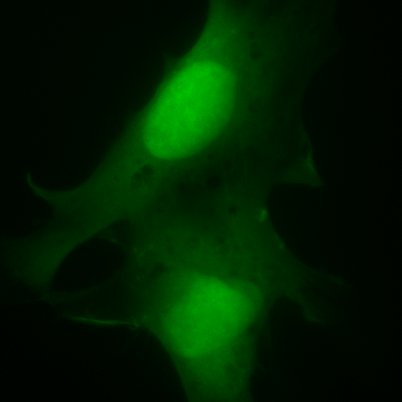

Supplement: Supplementary file 4 — Source Data for Figure 1 [file MSB-18-e11129-s006.zip › SD_Figure_1/1D/Processed/FoxA2 - dox/GFP.jpg]

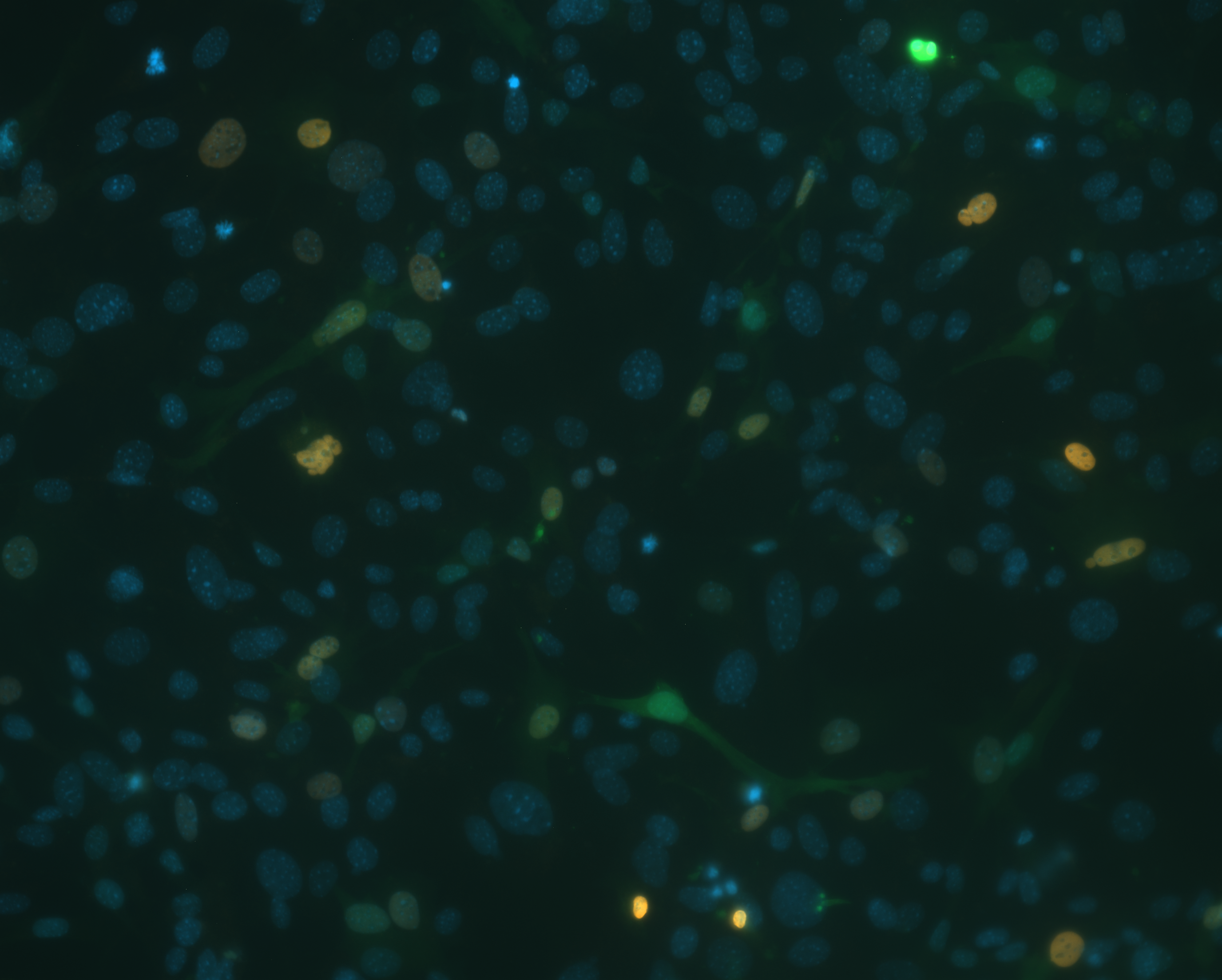

Supplement: Supplementary file 4 — Source Data for Figure 1 [file MSB-18-e11129-s006.zip › SD_Figure_1/1D/Raw/Sox2 + dox.tif]

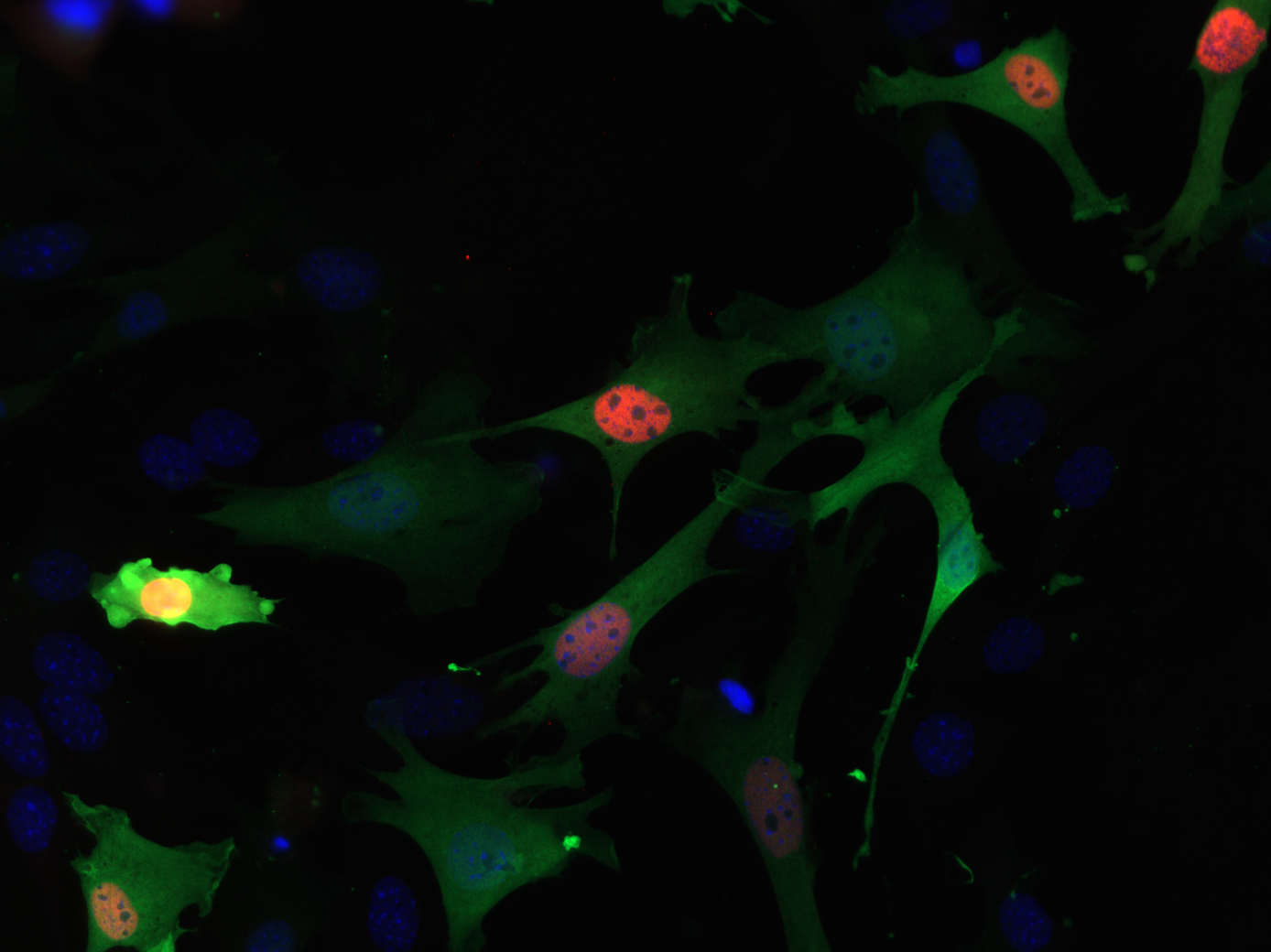

Supplement: Supplementary file 4 — Source Data for Figure 1 [file MSB-18-e11129-s006.zip › SD_Figure_1/1D/Raw/MyoD1 + dox.tif]

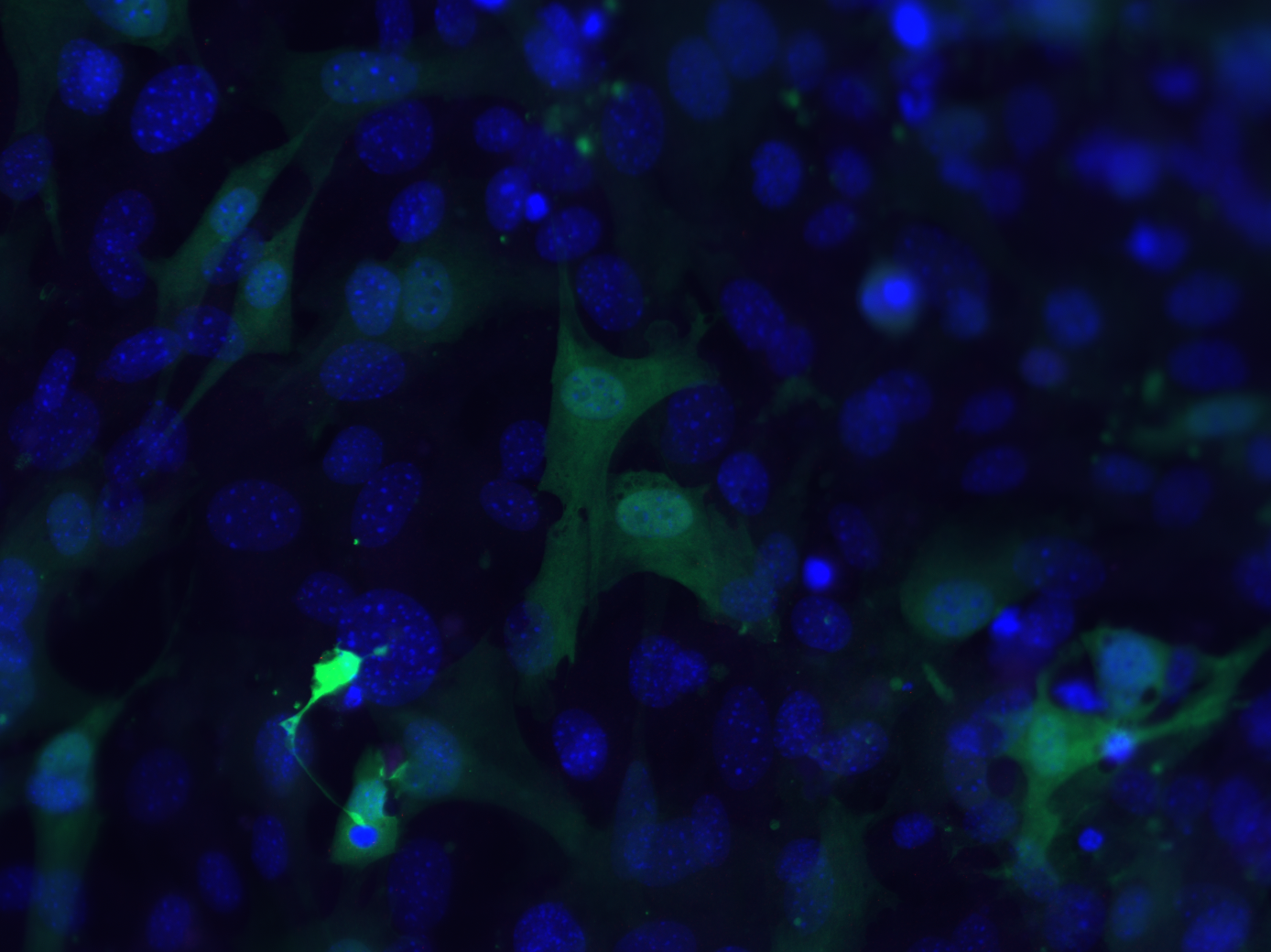

Supplement: Supplementary file 4 — Source Data for Figure 1 [file MSB-18-e11129-s006.zip › SD_Figure_1/1D/Raw/Ascl1 - dox.tif]

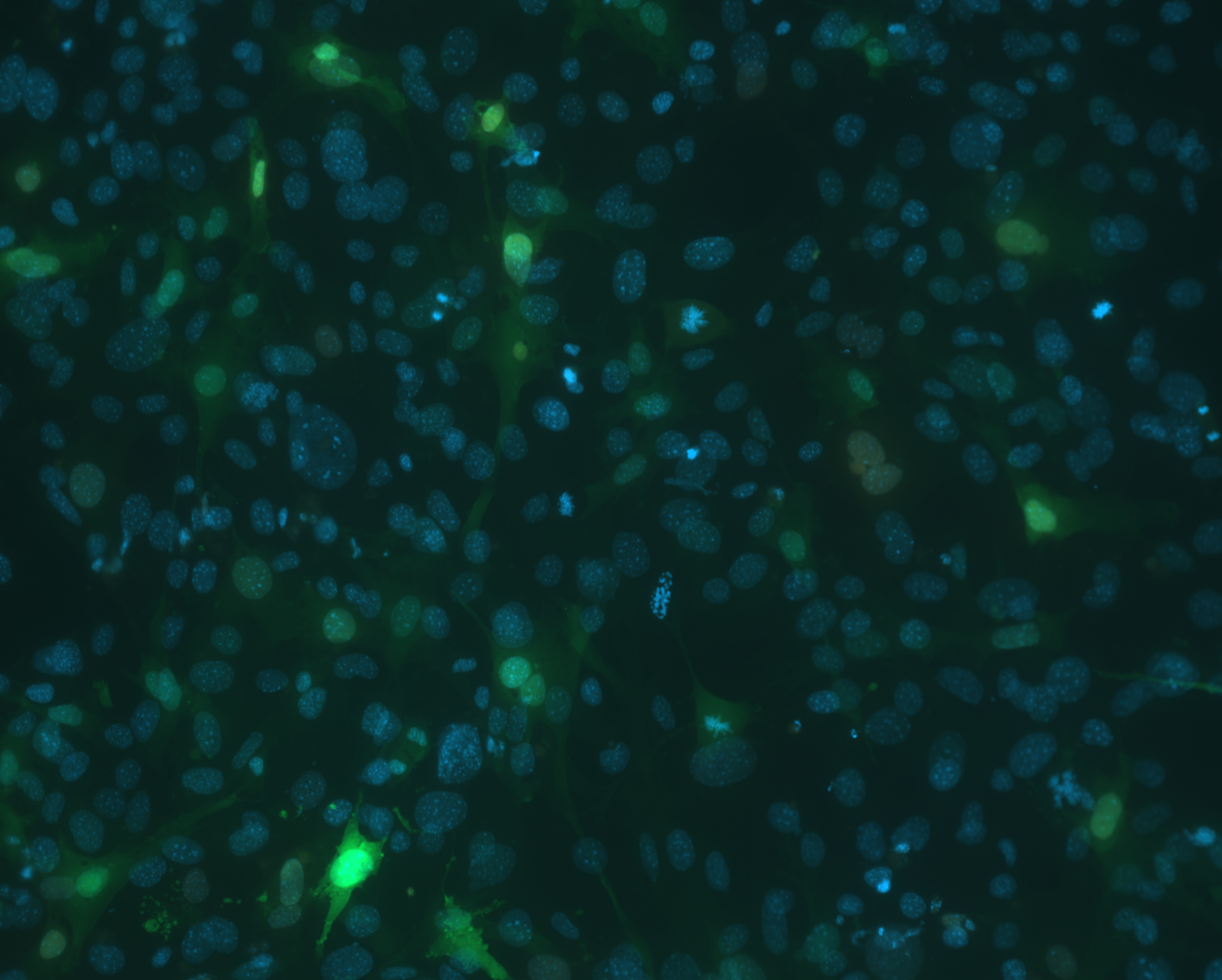

Supplement: Supplementary file 4 — Source Data for Figure 1 [file MSB-18-e11129-s006.zip › SD_Figure_1/1D/Raw/FoxA2 + dox.tif]

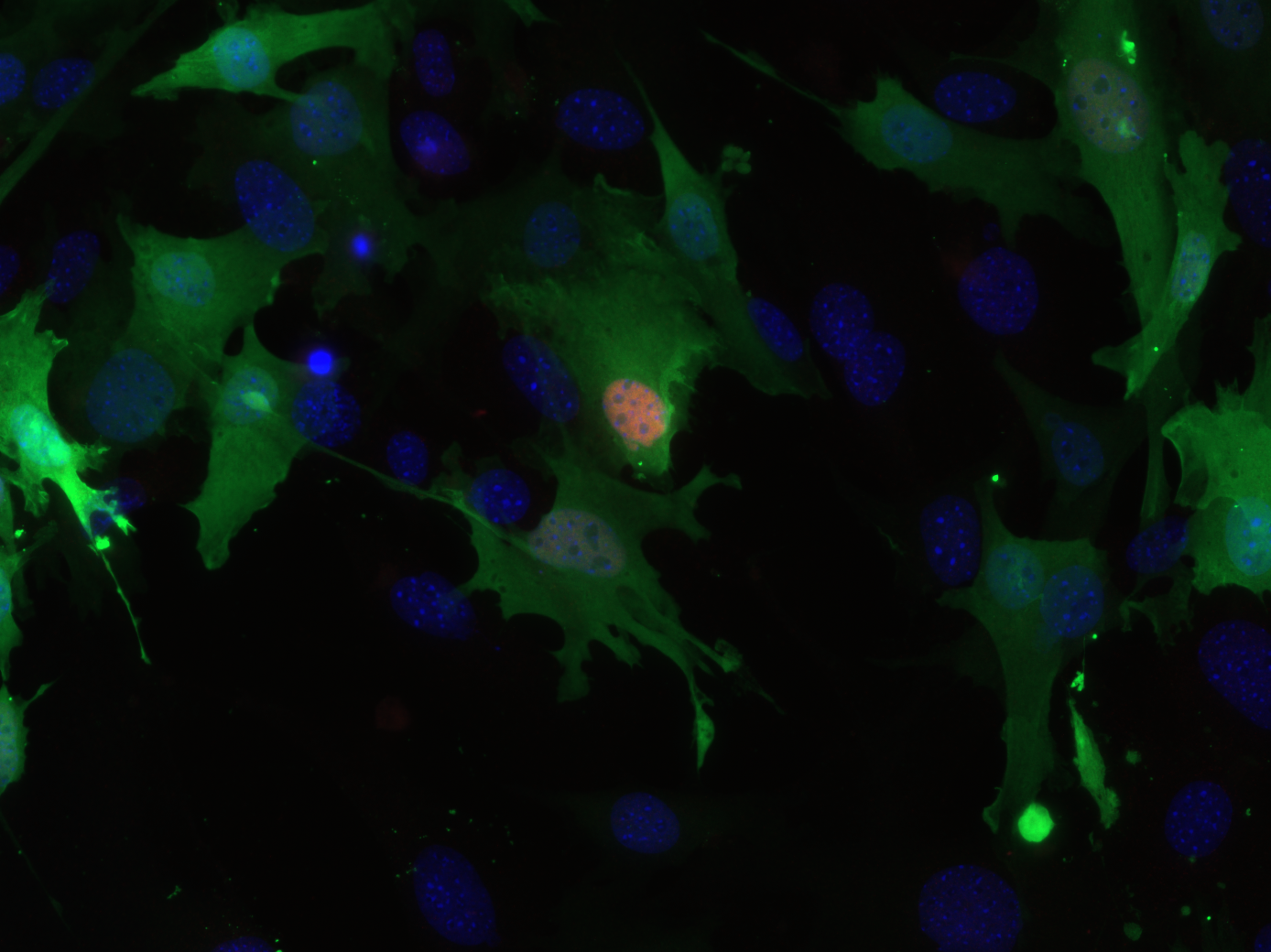

Supplement: Supplementary file 4 — Source Data for Figure 1 [file MSB-18-e11129-s006.zip › SD_Figure_1/1D/Raw/Oct4 + dox.tif]

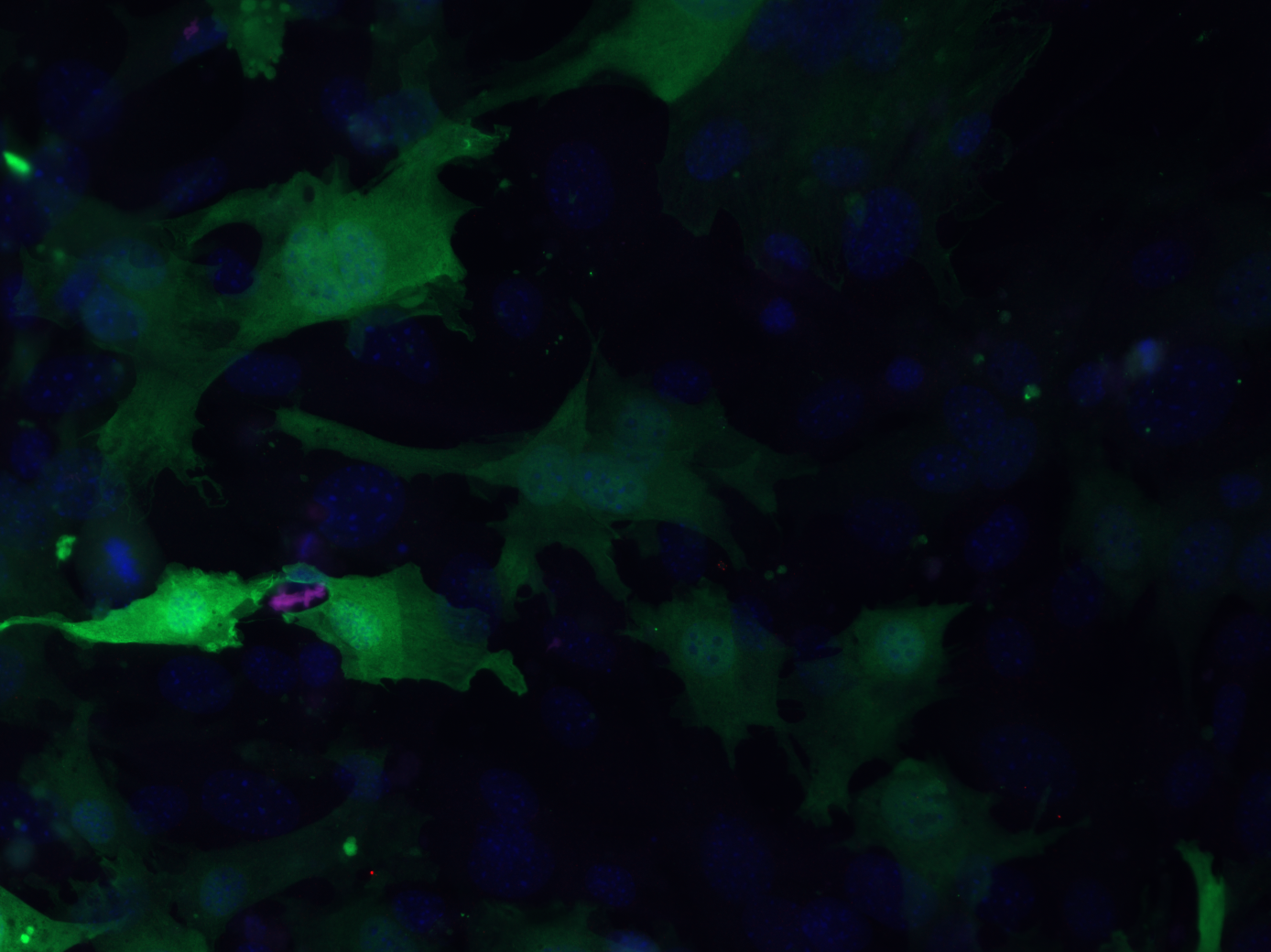

Supplement: Supplementary file 4 — Source Data for Figure 1 [file MSB-18-e11129-s006.zip › SD_Figure_1/1D/Raw/MyoD1 - dox.tif]

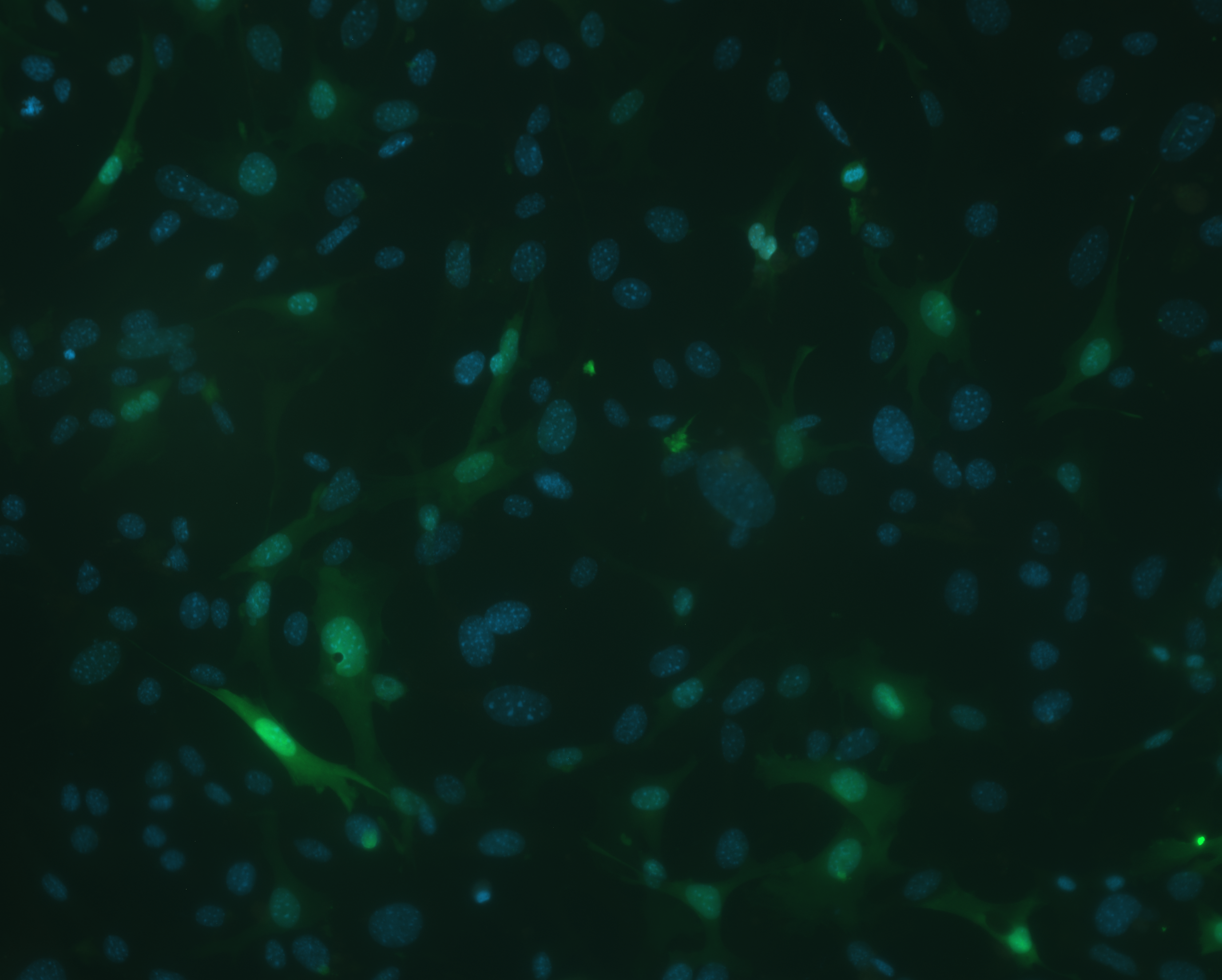

Supplement: Supplementary file 4 — Source Data for Figure 1 [file MSB-18-e11129-s006.zip › SD_Figure_1/1D/Raw/Sox2 - dox.tif]

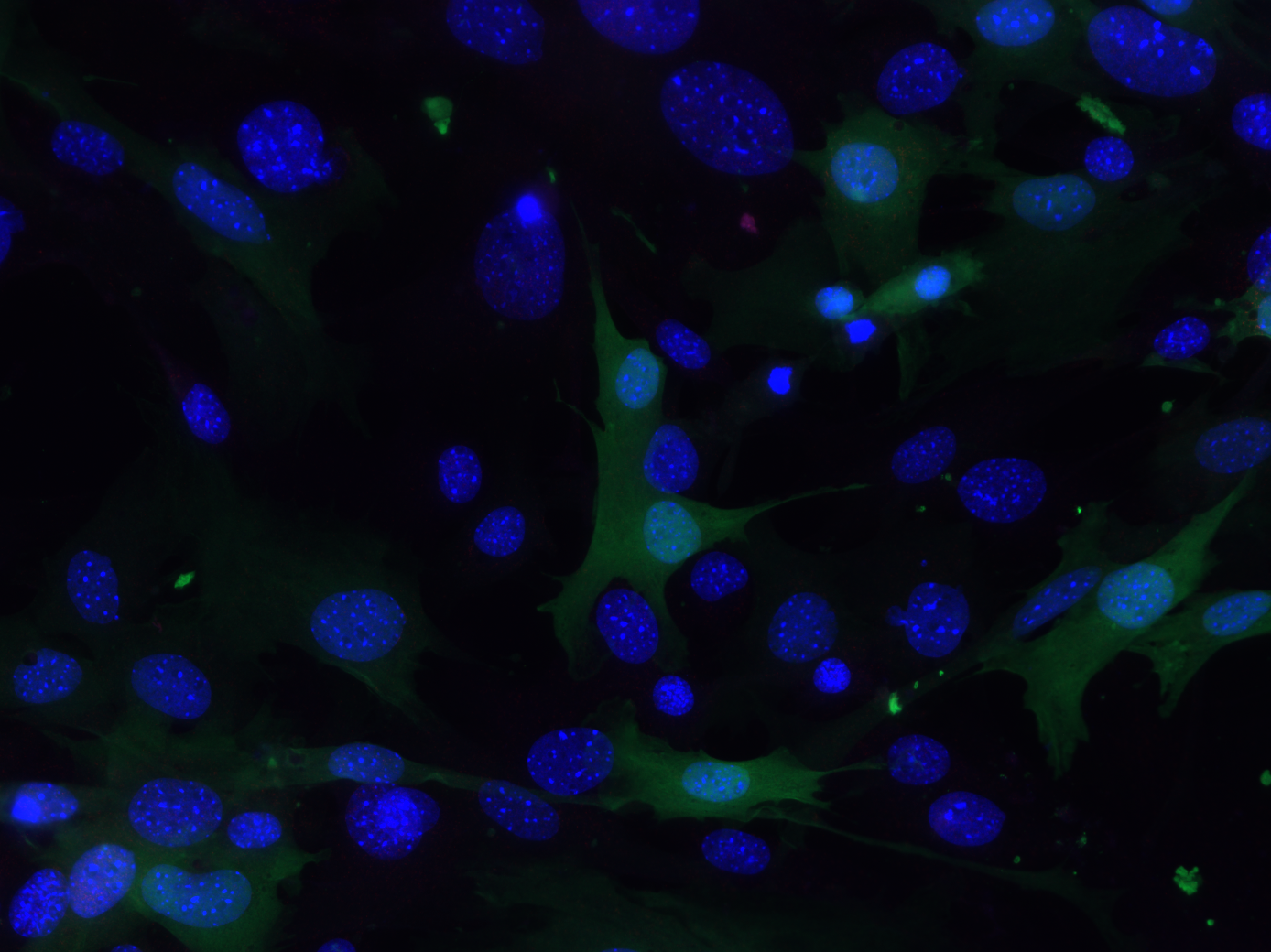

Supplement: Supplementary file 4 — Source Data for Figure 1 [file MSB-18-e11129-s006.zip › SD_Figure_1/1D/Raw/Oct4 - dox.tif]

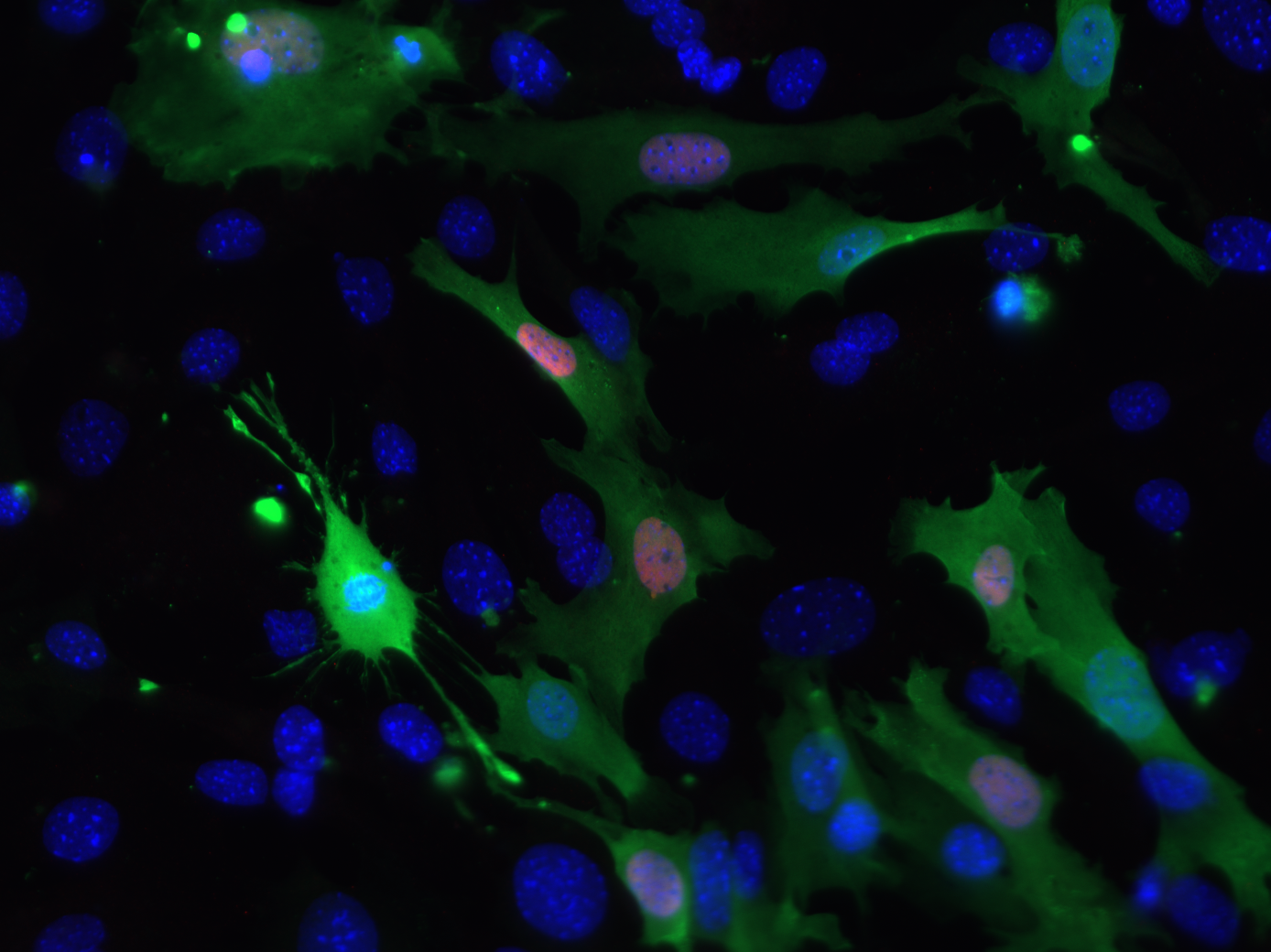

Supplement: Supplementary file 4 — Source Data for Figure 1 [file MSB-18-e11129-s006.zip › SD_Figure_1/1D/Raw/Ascl1 + dox.tif]

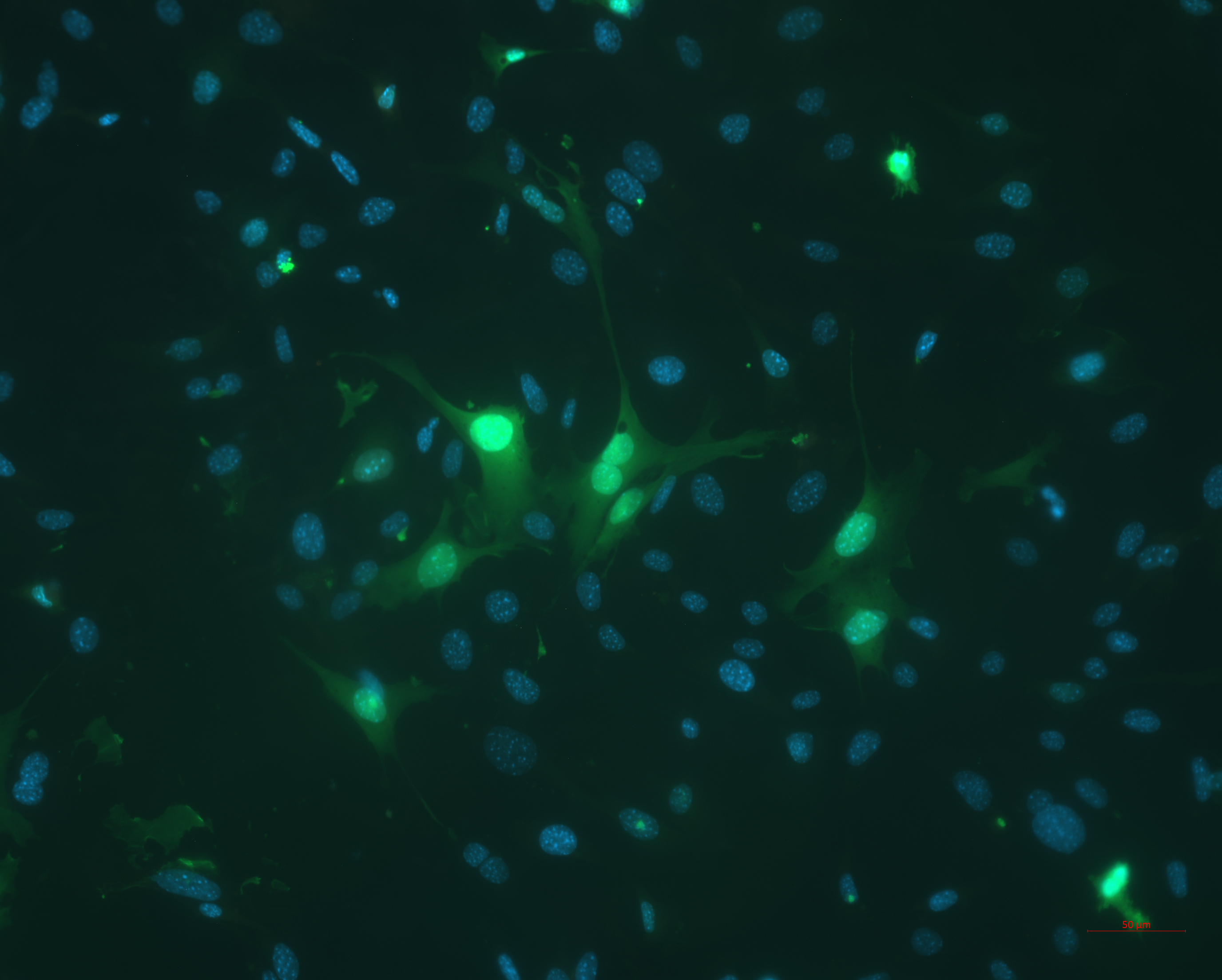

Supplement: Supplementary file 4 — Source Data for Figure 1 [file MSB-18-e11129-s006.zip › SD_Figure_1/1D/Raw/FoxA2 - dox.tif]

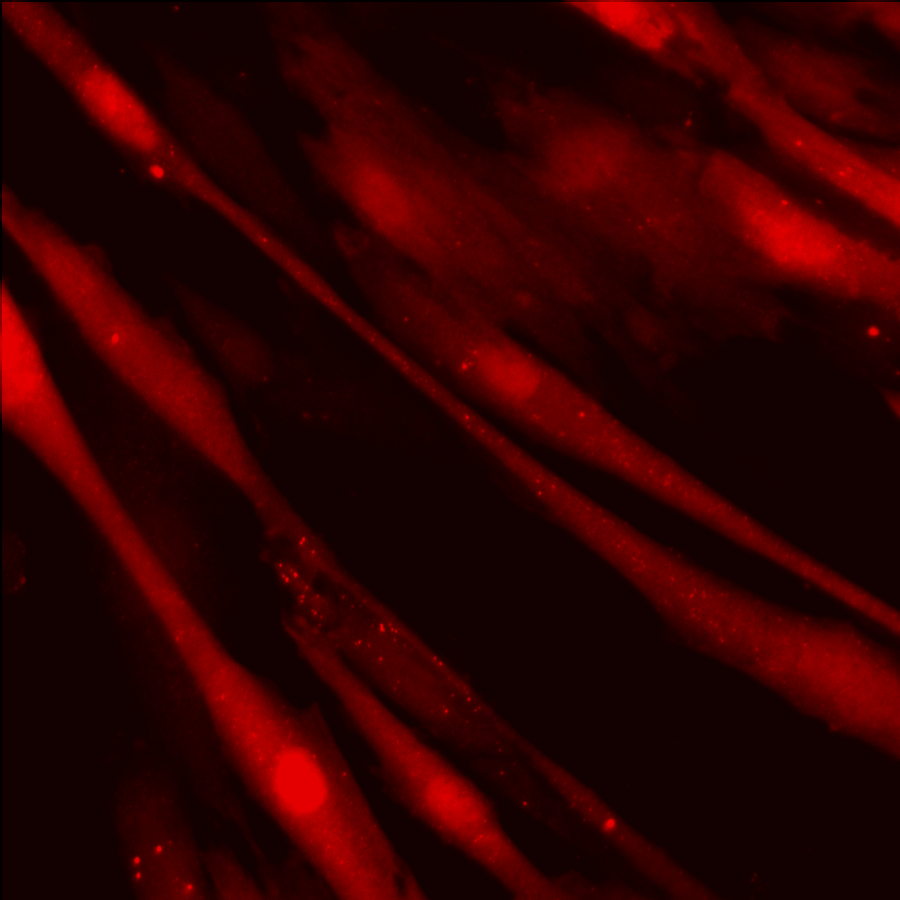

Supplement: Supplementary file 5 — Source Data for Figure 5 [file MSB-18-e11129-s004.zip › SD_Figure_5/5F/Processed/MyoD1/RFP.tif]

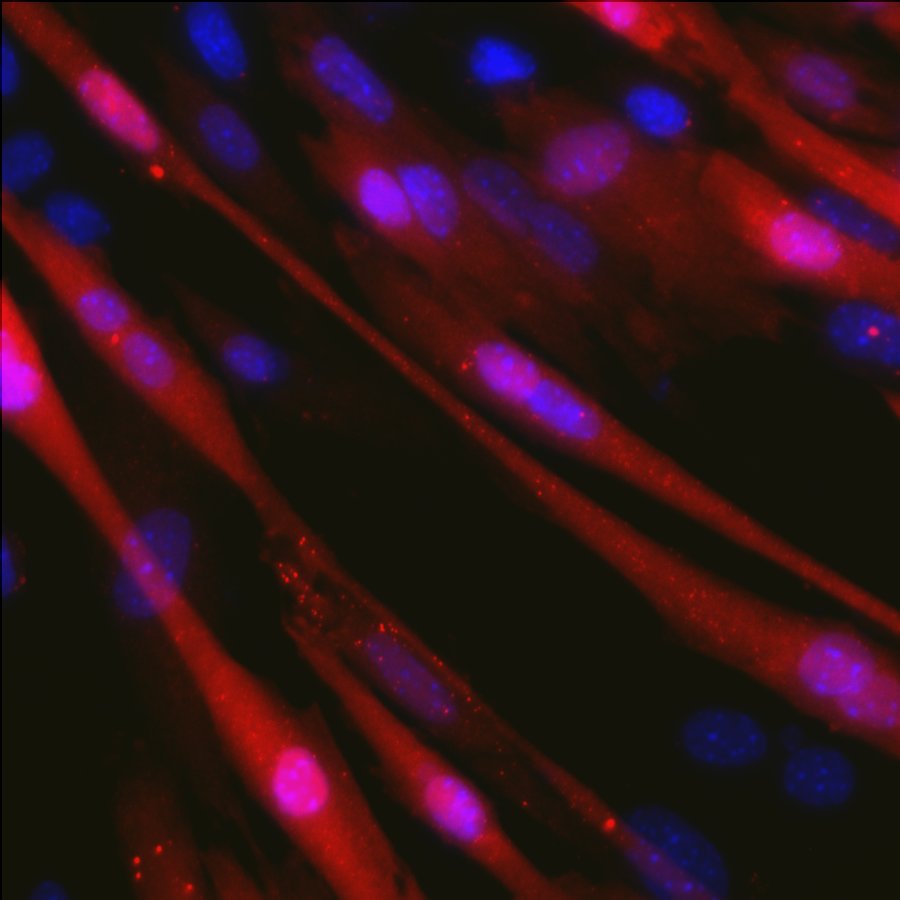

Supplement: Supplementary file 5 — Source Data for Figure 5 [file MSB-18-e11129-s004.zip › SD_Figure_5/5F/Processed/MyoD1/Merge.jpg]

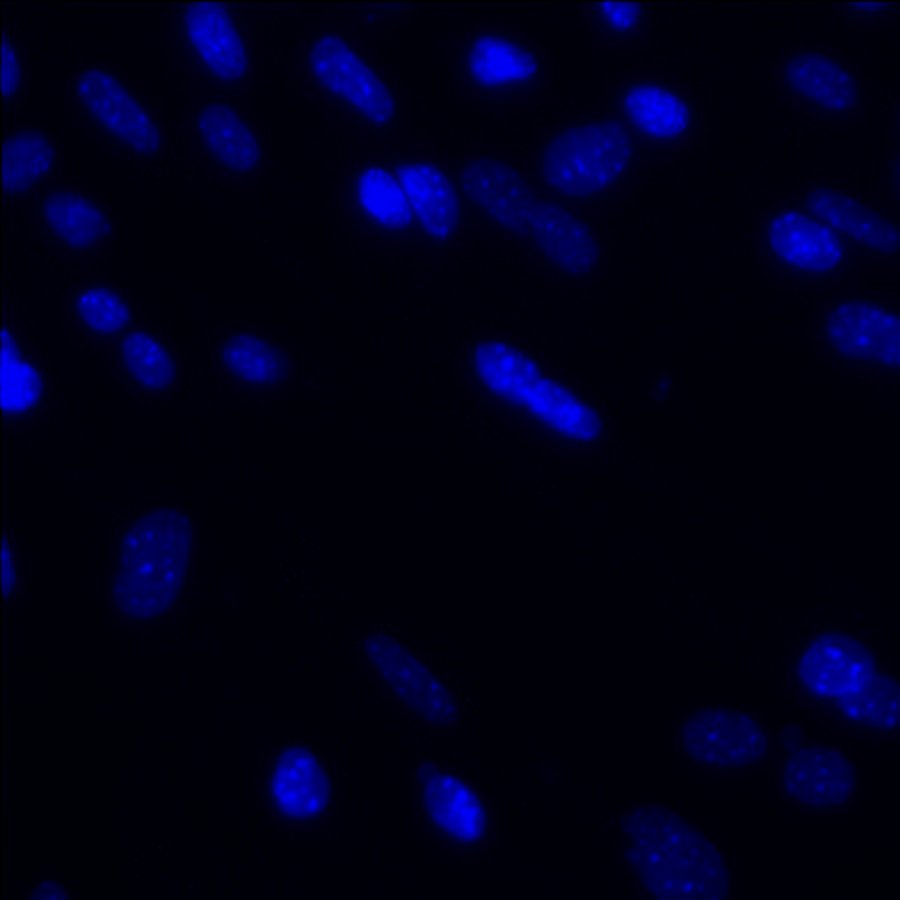

Supplement: Supplementary file 5 — Source Data for Figure 5 [file MSB-18-e11129-s004.zip › SD_Figure_5/5F/Processed/MyoD1/DAPI.jpg]

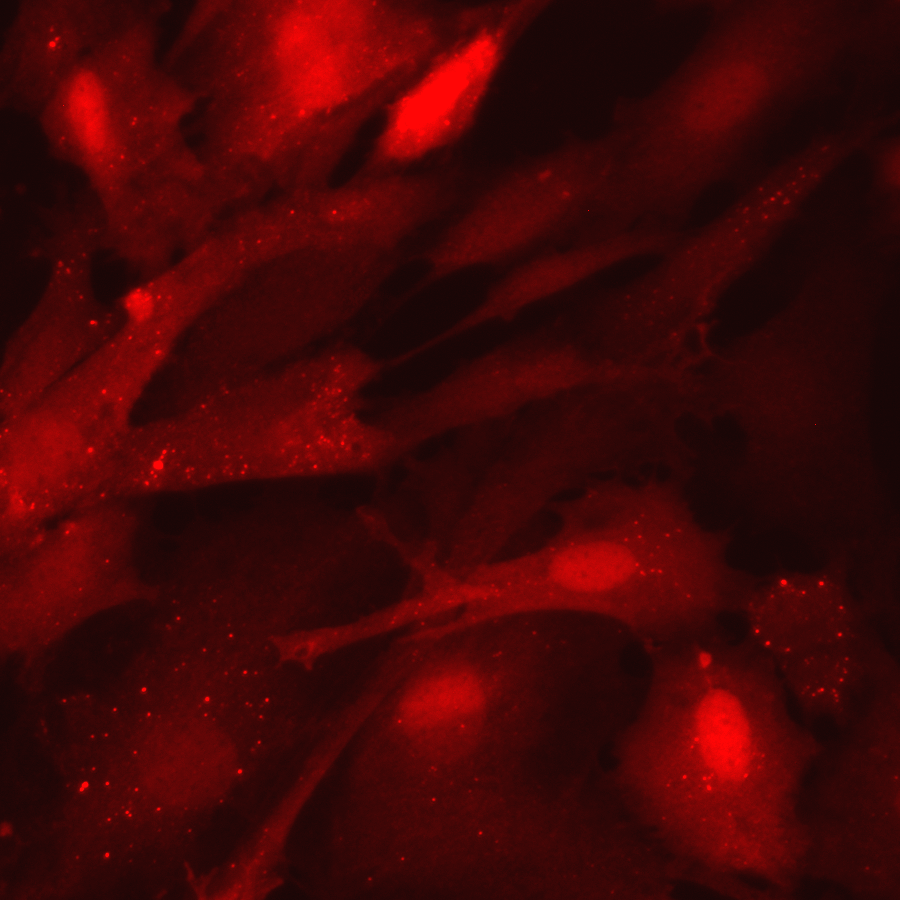

Supplement: Supplementary file 5 — Source Data for Figure 5 [file MSB-18-e11129-s004.zip › SD_Figure_5/5F/Processed/Ascl1 & MyoD1/RFP.tif]

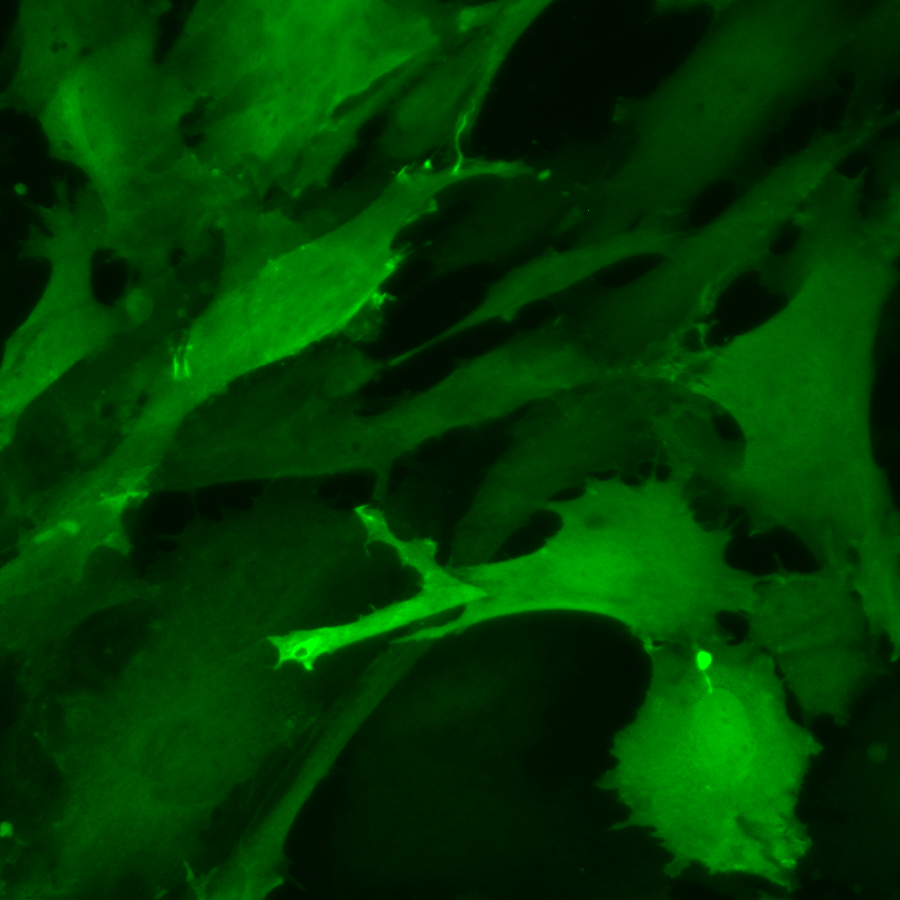

Supplement: Supplementary file 5 — Source Data for Figure 5 [file MSB-18-e11129-s004.zip › SD_Figure_5/5F/Processed/Ascl1 & MyoD1/GFP.tif]

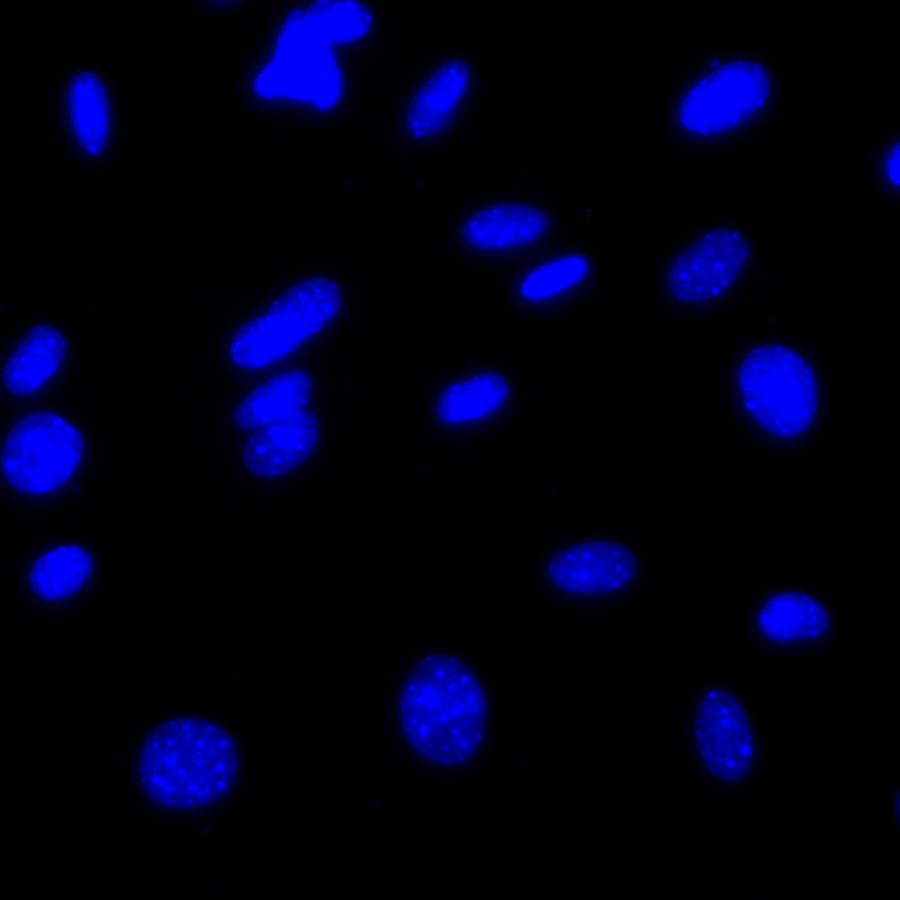

Supplement: Supplementary file 5 — Source Data for Figure 5 [file MSB-18-e11129-s004.zip › SD_Figure_5/5F/Processed/Ascl1 & MyoD1/DAPI.tif]

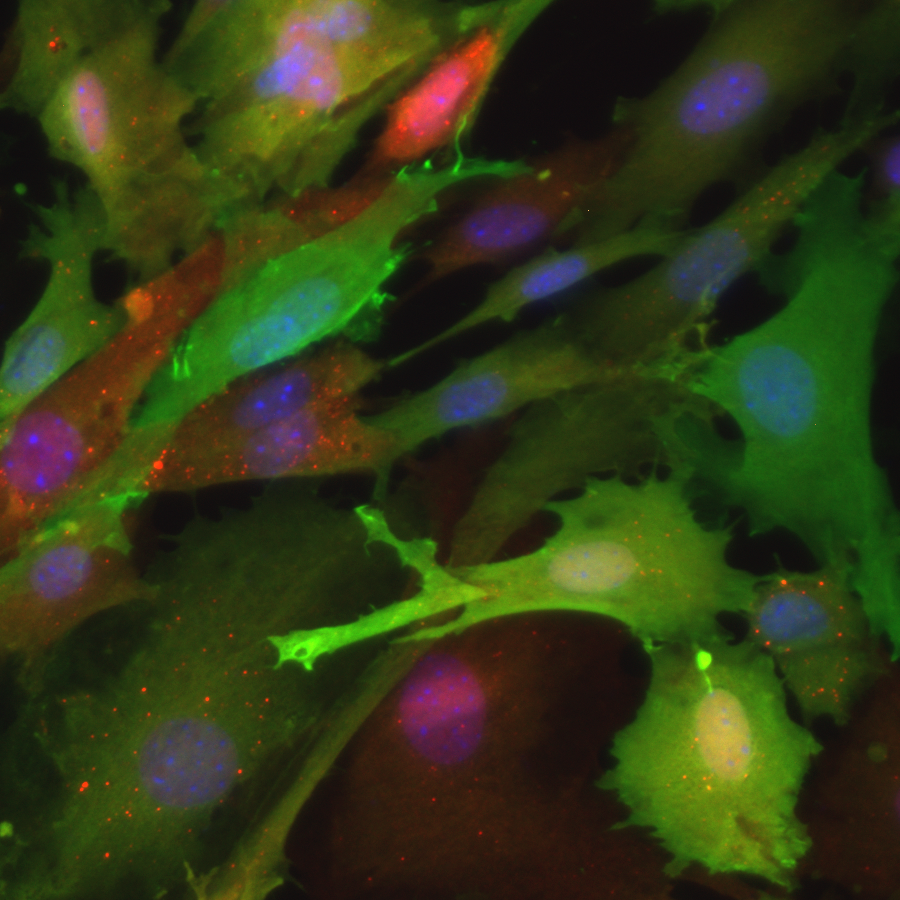

Supplement: Supplementary file 5 — Source Data for Figure 5 [file MSB-18-e11129-s004.zip › SD_Figure_5/5F/Processed/Ascl1 & MyoD1/Merge.tif]

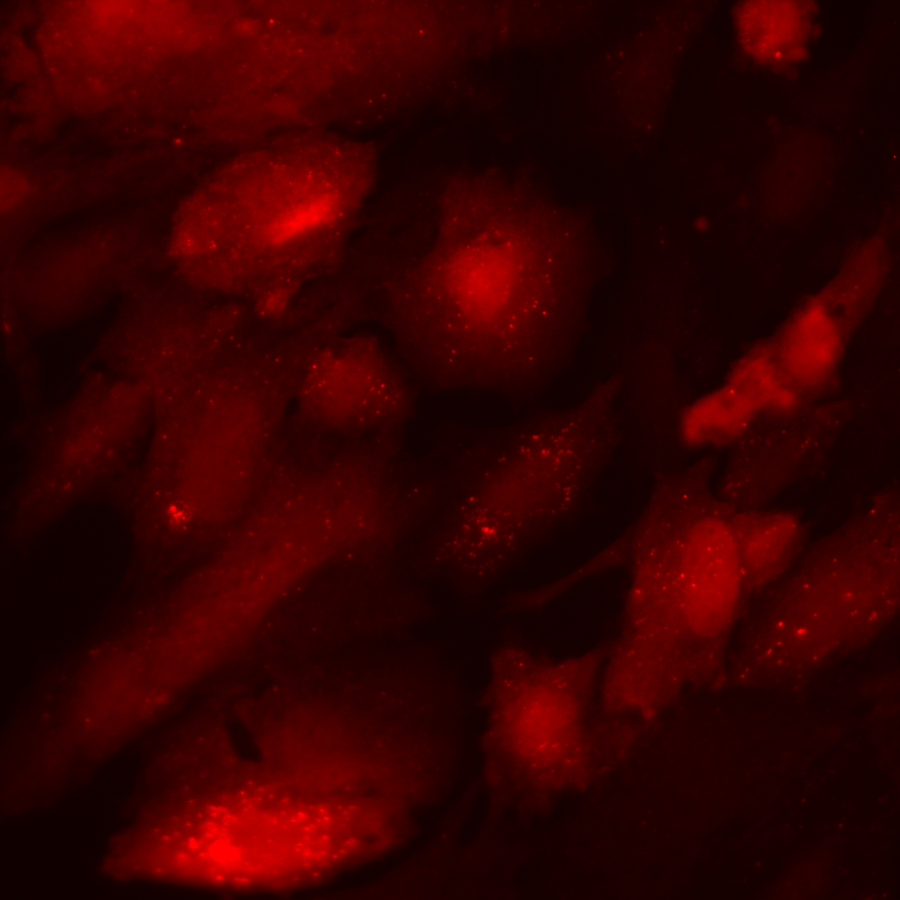

Supplement: Supplementary file 5 — Source Data for Figure 5 [file MSB-18-e11129-s004.zip › SD_Figure_5/5F/Processed/mutAscl1 & MyoD1/RFP.tif]

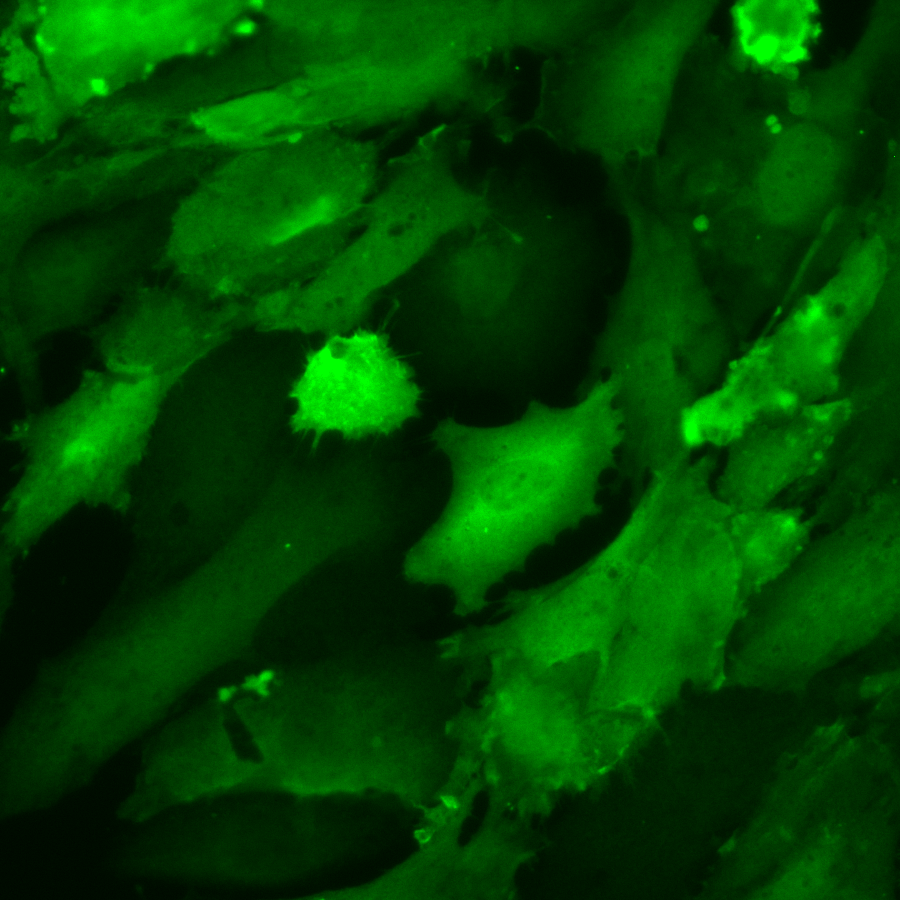

Supplement: Supplementary file 5 — Source Data for Figure 5 [file MSB-18-e11129-s004.zip › SD_Figure_5/5F/Processed/mutAscl1 & MyoD1/GFP.tif]

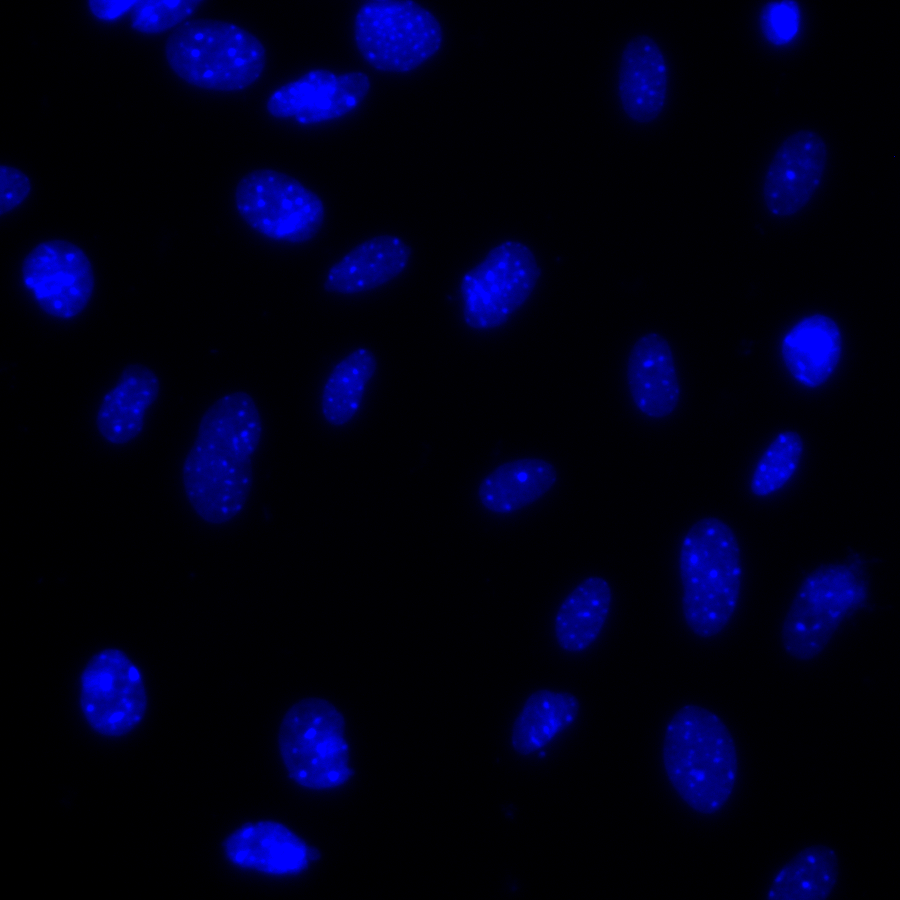

Supplement: Supplementary file 5 — Source Data for Figure 5 [file MSB-18-e11129-s004.zip › SD_Figure_5/5F/Processed/mutAscl1 & MyoD1/DAPI.tif]

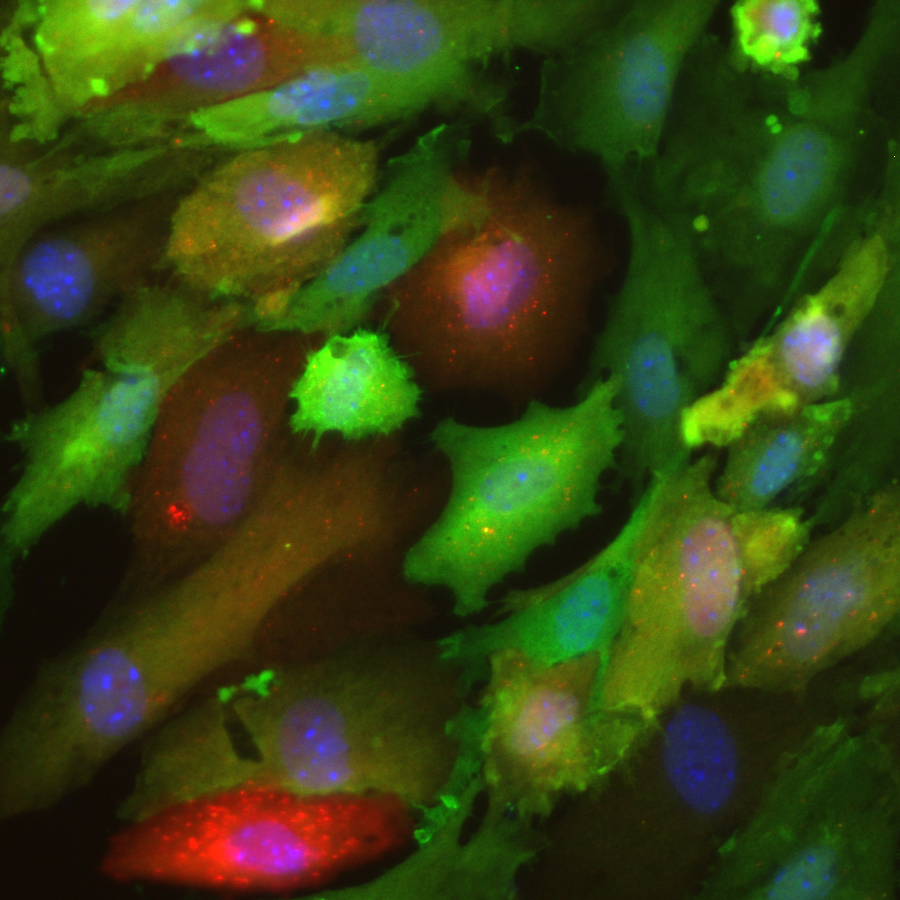

Supplement: Supplementary file 5 — Source Data for Figure 5 [file MSB-18-e11129-s004.zip › SD_Figure_5/5F/Processed/mutAscl1 & MyoD1/Merge.tif]

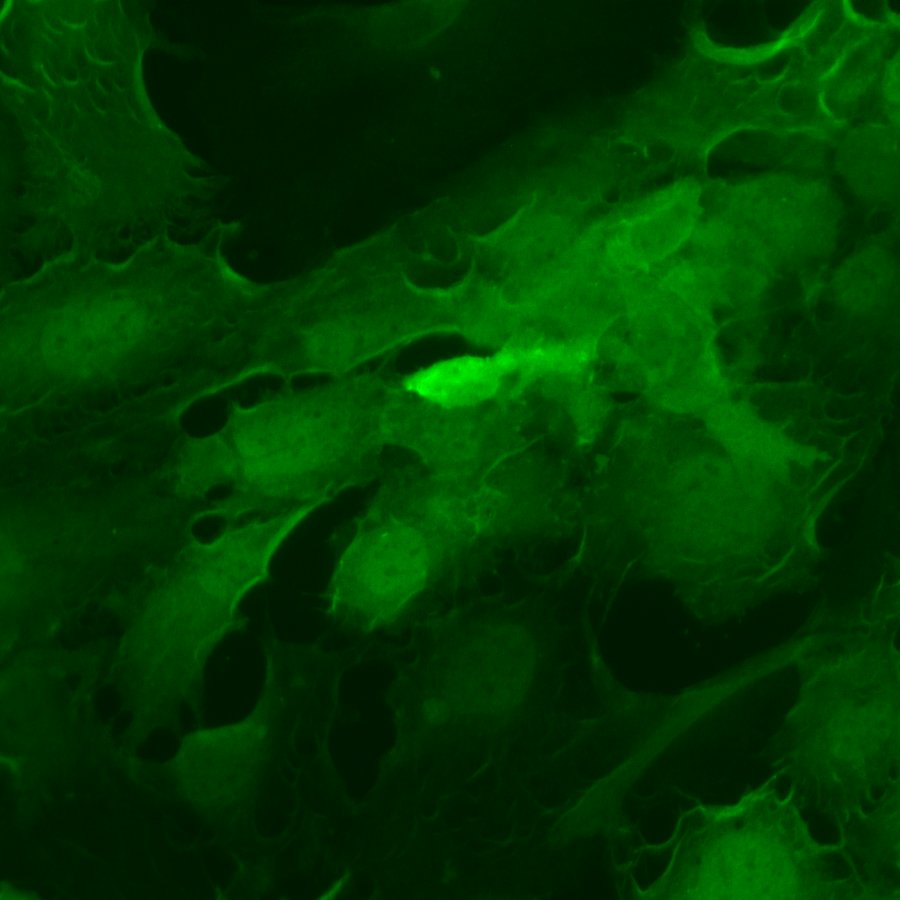

Supplement: Supplementary file 5 — Source Data for Figure 5 [file MSB-18-e11129-s004.zip › SD_Figure_5/5F/Processed/Ascl1/GFP.tif]

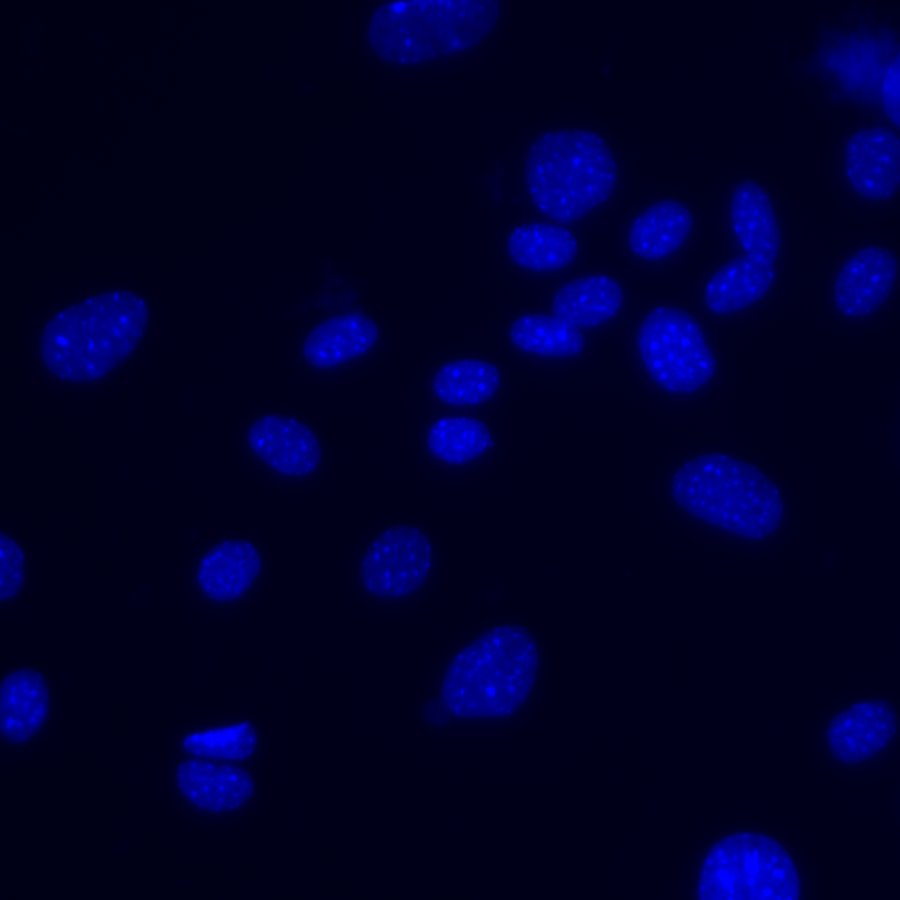

Supplement: Supplementary file 5 — Source Data for Figure 5 [file MSB-18-e11129-s004.zip › SD_Figure_5/5F/Processed/Ascl1/DAPI.tif]

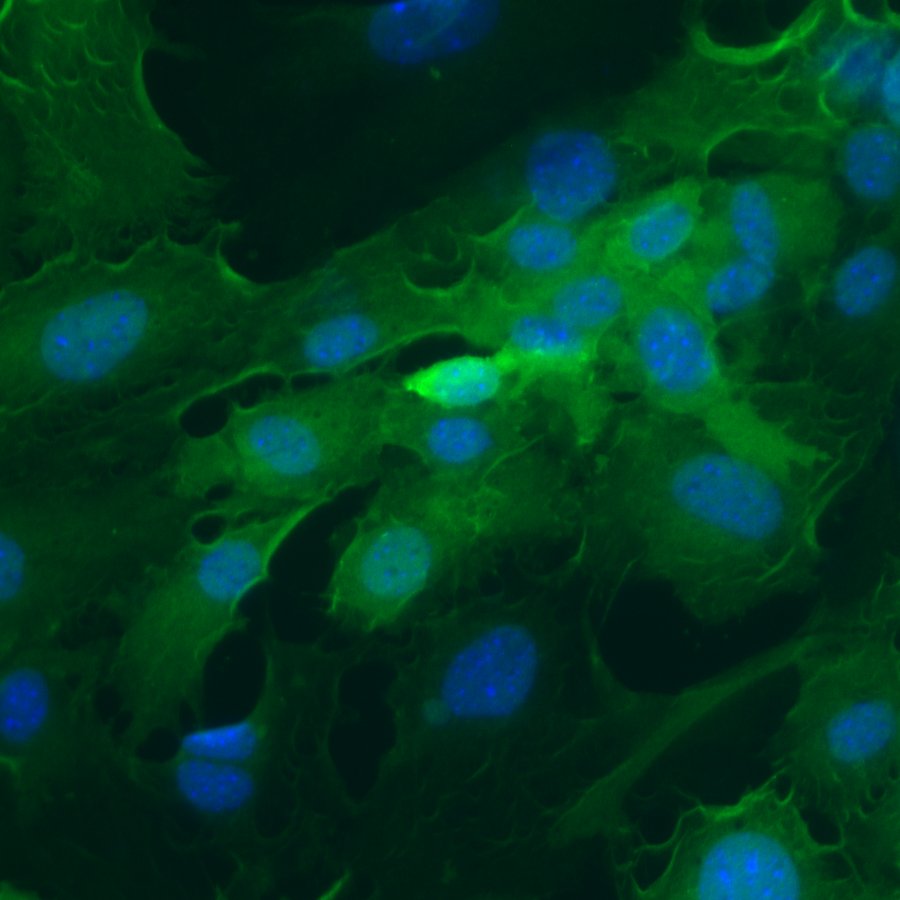

Supplement: Supplementary file 5 — Source Data for Figure 5 [file MSB-18-e11129-s004.zip › SD_Figure_5/5F/Processed/Ascl1/Merge.tif]

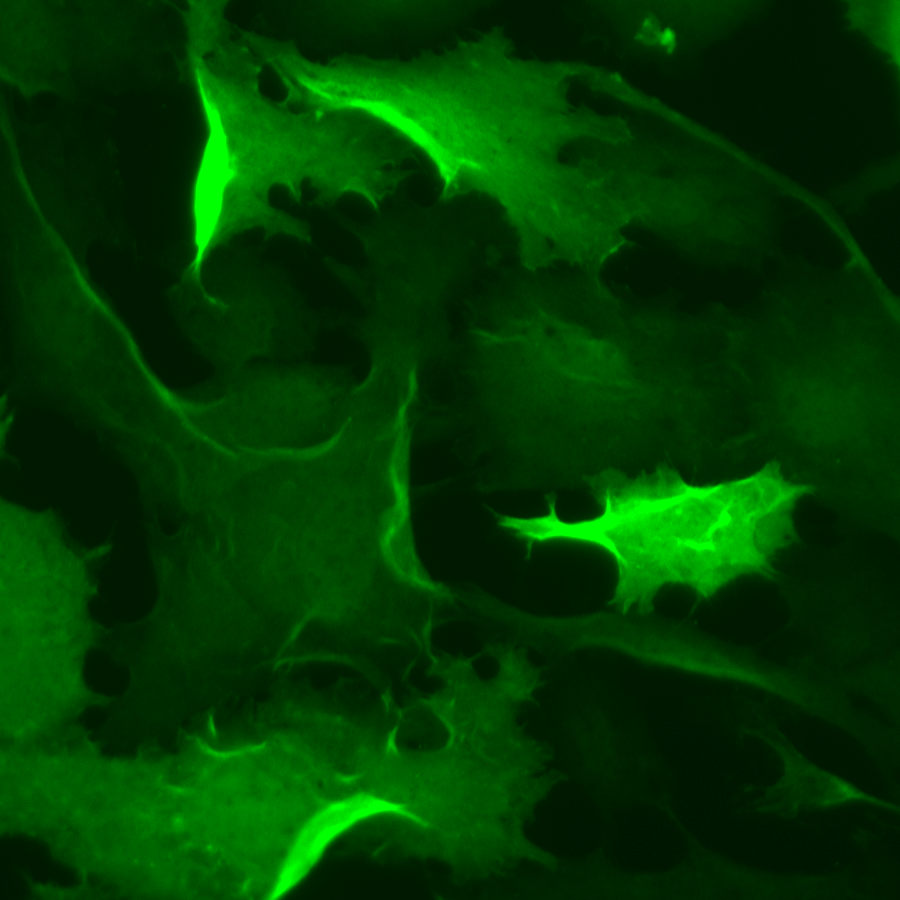

Supplement: Supplementary file 5 — Source Data for Figure 5 [file MSB-18-e11129-s004.zip › SD_Figure_5/5F/Processed/mutAscl1/GFP.tif]

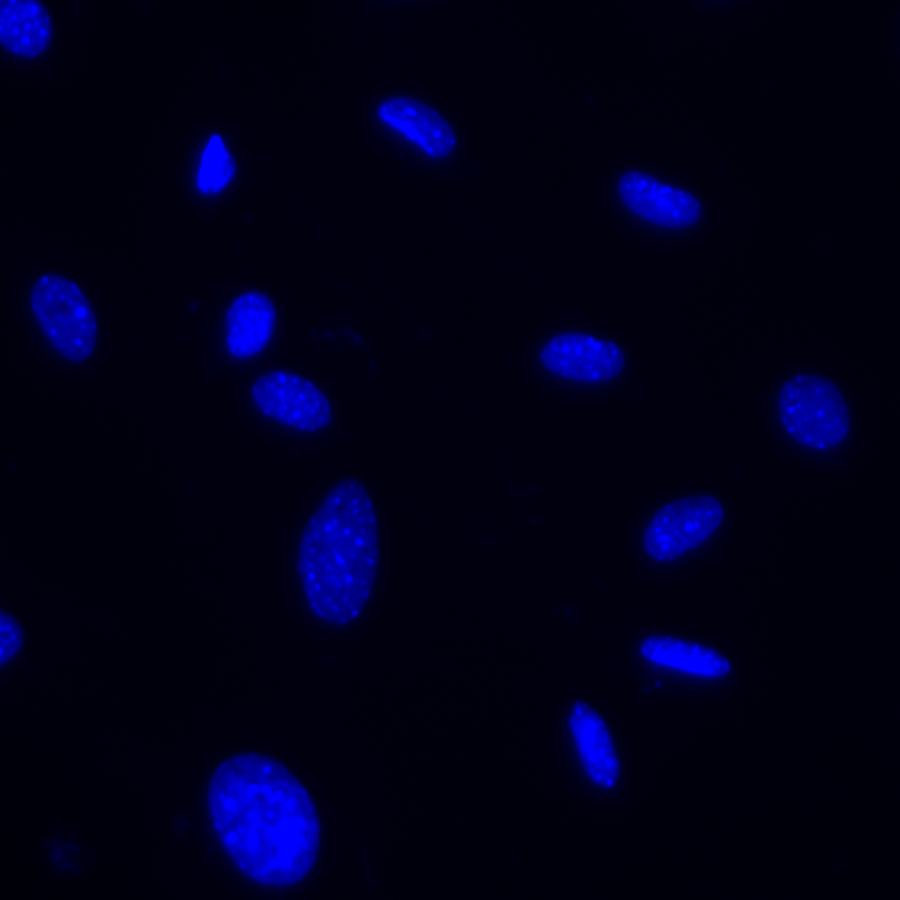

Supplement: Supplementary file 5 — Source Data for Figure 5 [file MSB-18-e11129-s004.zip › SD_Figure_5/5F/Processed/mutAscl1/DAPI.tif]

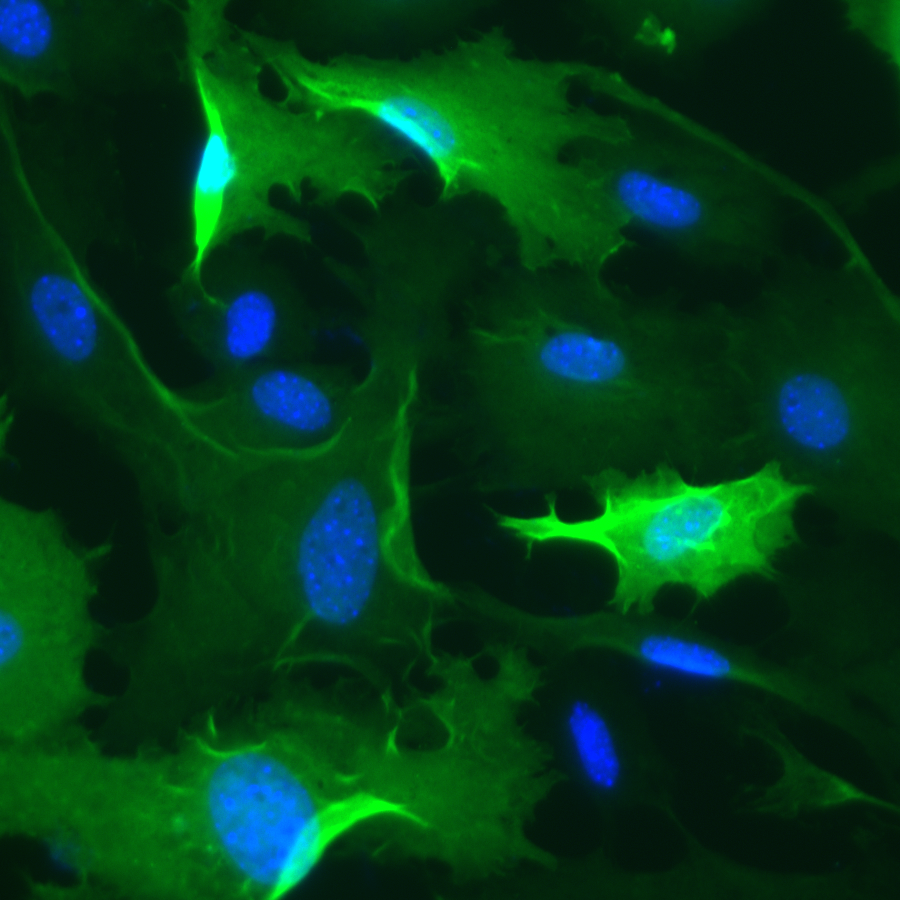

Supplement: Supplementary file 5 — Source Data for Figure 5 [file MSB-18-e11129-s004.zip › SD_Figure_5/5F/Processed/mutAscl1/Merge.tif]

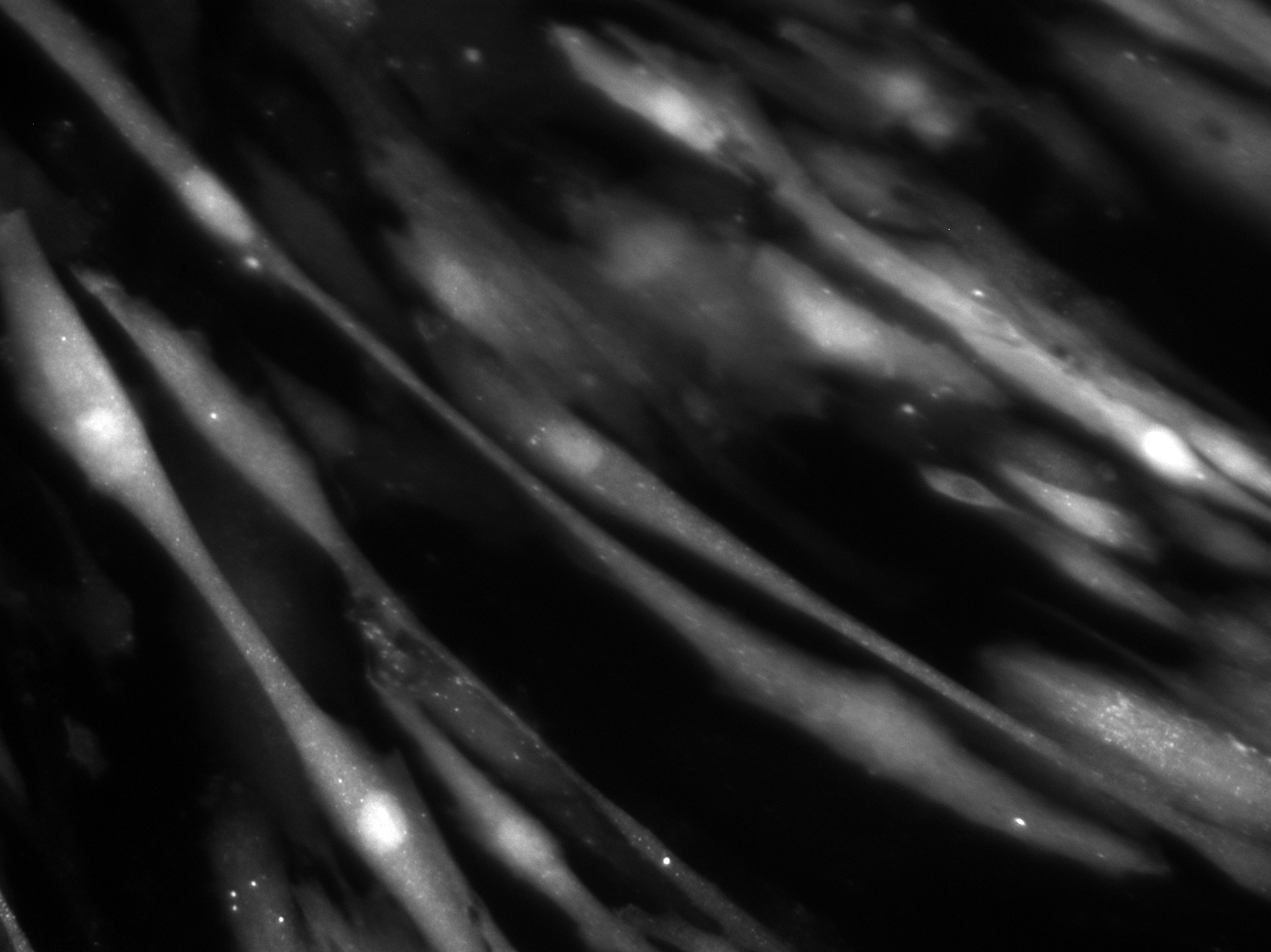

Supplement: Supplementary file 5 — Source Data for Figure 5 [file MSB-18-e11129-s004.zip › SD_Figure_5/5F/Raw/MyoD1.tif]

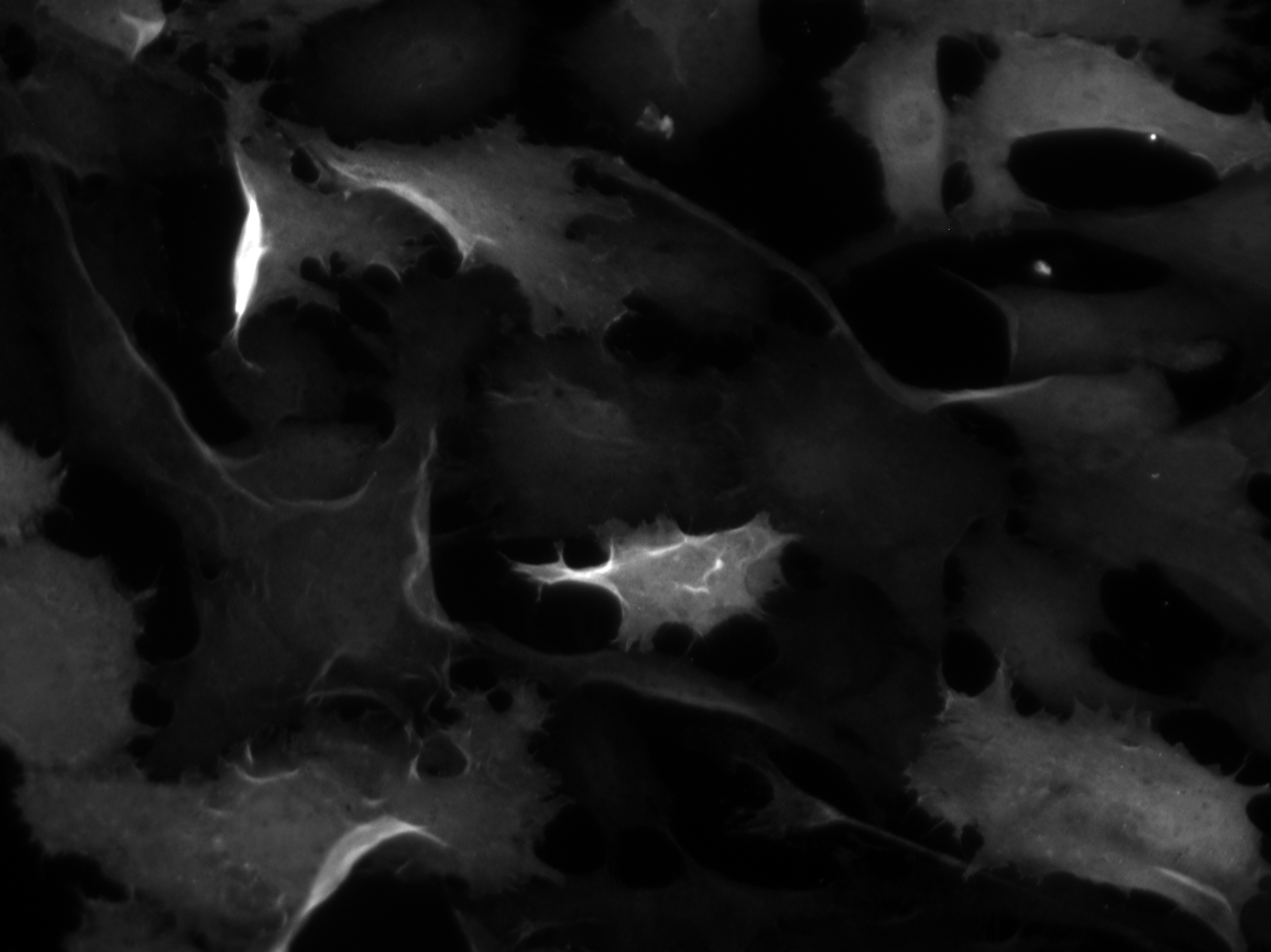

Supplement: Supplementary file 5 — Source Data for Figure 5 [file MSB-18-e11129-s004.zip › SD_Figure_5/5F/Raw/mutAscl1.tif]

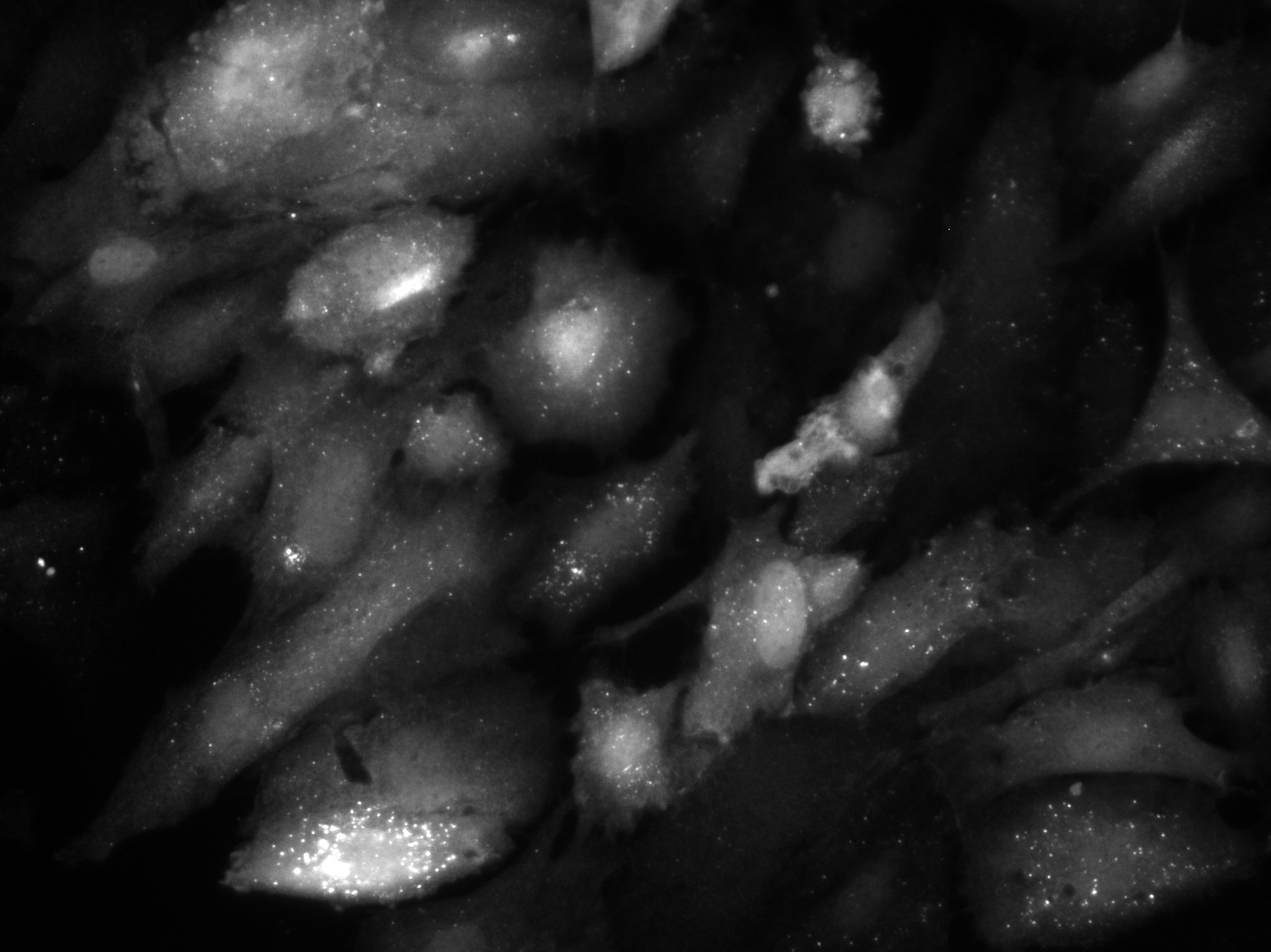

Supplement: Supplementary file 5 — Source Data for Figure 5 [file MSB-18-e11129-s004.zip › SD_Figure_5/5F/Raw/mutAscl1 & MyoD1.tif]

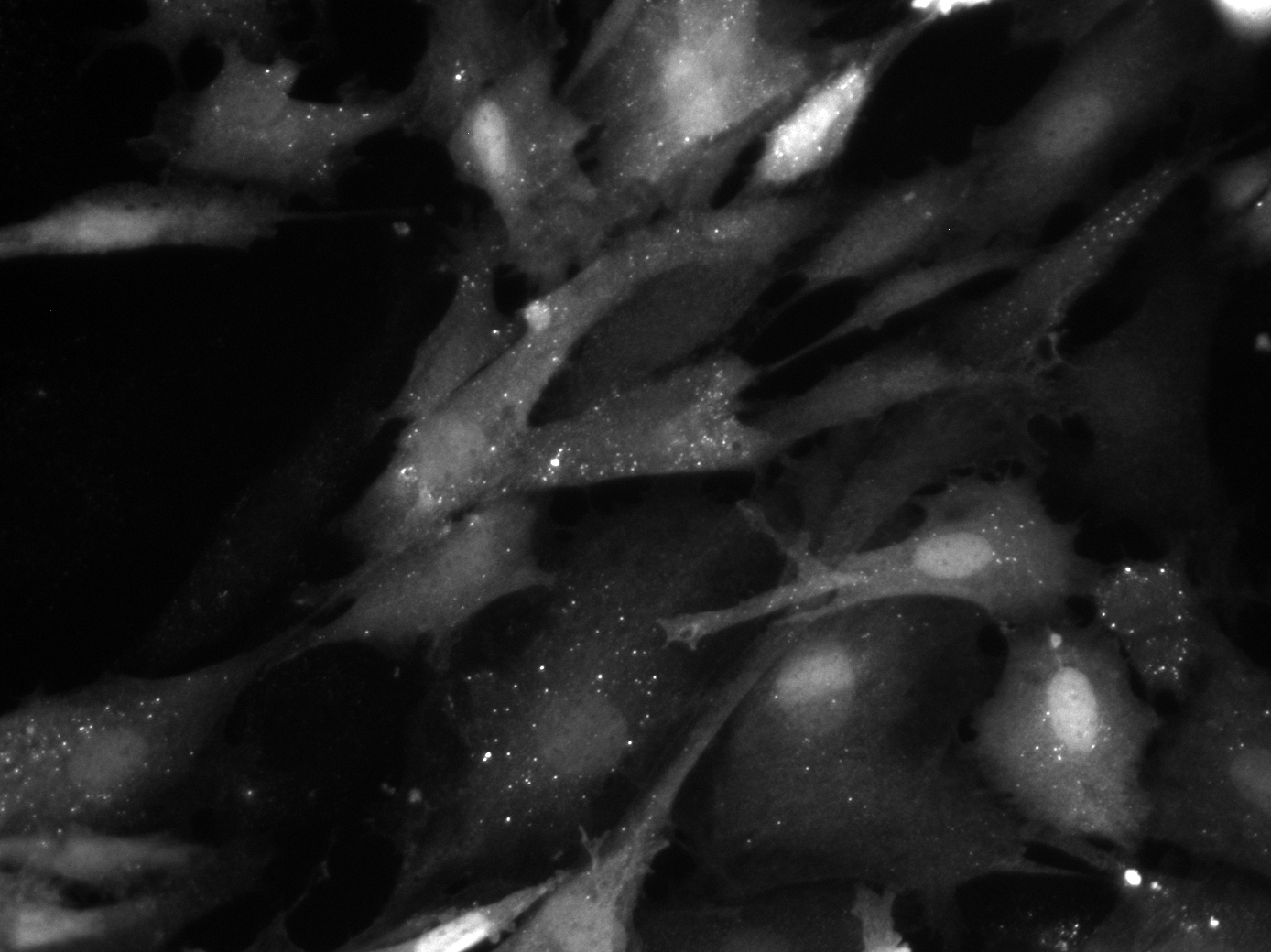

Supplement: Supplementary file 5 — Source Data for Figure 5 [file MSB-18-e11129-s004.zip › SD_Figure_5/5F/Raw/Ascl1 & MyoD1.tif]

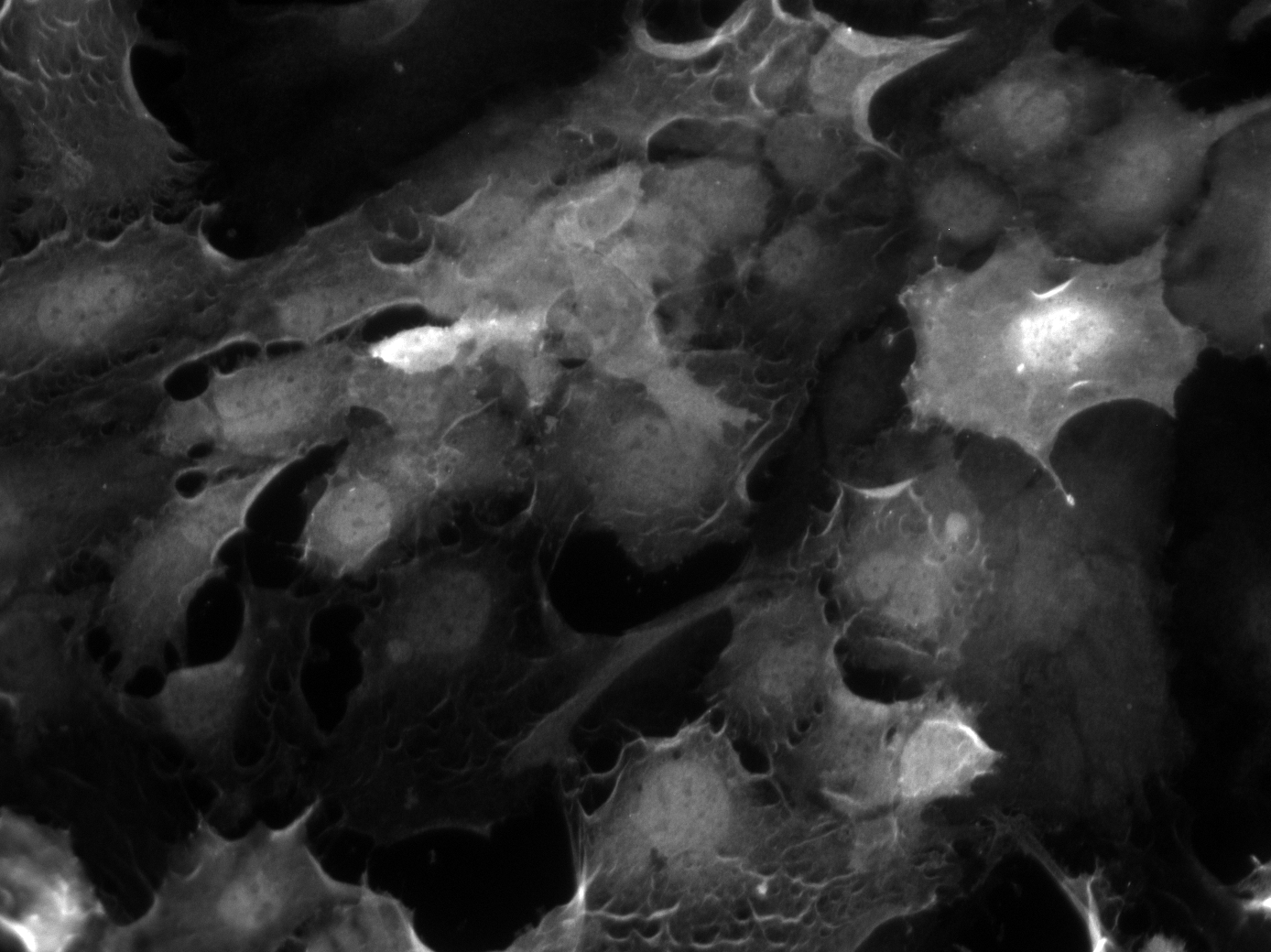

Supplement: Supplementary file 5 — Source Data for Figure 5 [file MSB-18-e11129-s004.zip › SD_Figure_5/5F/Raw/Ascl1.tif]

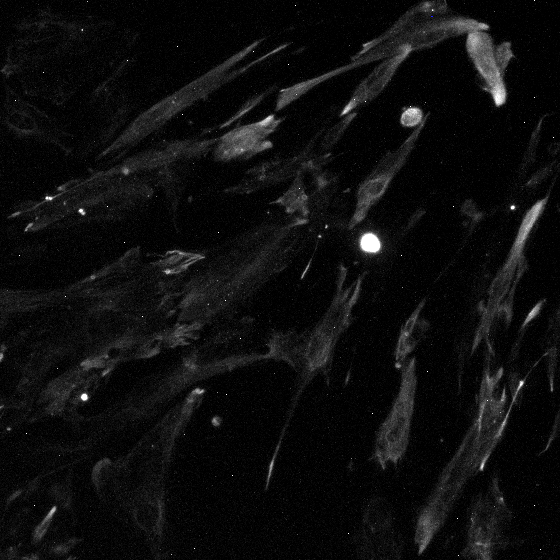

Supplement: Supplementary file 6 — Source Data for Expanded View [file MSB-18-e11129-s007.zip › Figure EV3/EV3I/Processed/Ascl1 & MyoD1 - Desmin.tif]

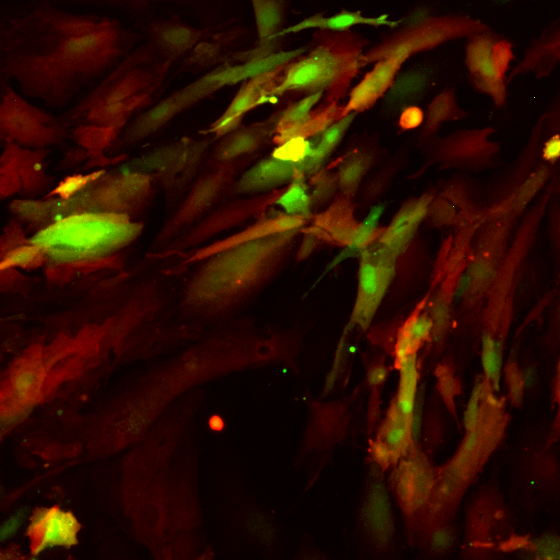

Supplement: Supplementary file 6 — Source Data for Expanded View [file MSB-18-e11129-s007.zip › Figure EV3/EV3I/Processed/Ascl1 & MyoD1 - GFP & RFP.tif]

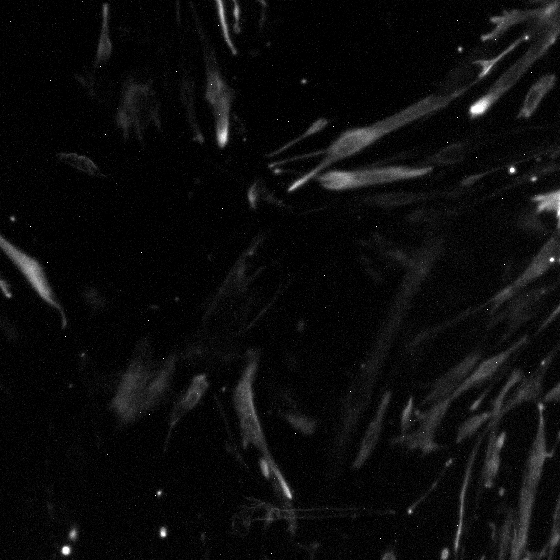

Supplement: Supplementary file 6 — Source Data for Expanded View [file MSB-18-e11129-s007.zip › Figure EV3/EV3I/Processed/mutantAscl1 & MyoD1 - Desmin.tif]

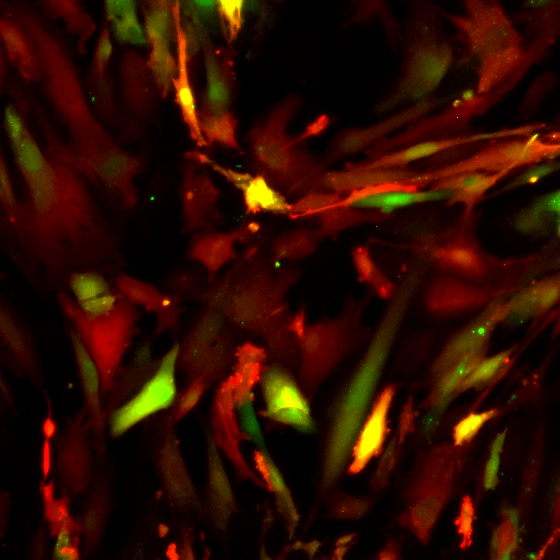

Supplement: Supplementary file 6 — Source Data for Expanded View [file MSB-18-e11129-s007.zip › Figure EV3/EV3I/Processed/mutantAscl1 & MyoD1 - GFP & RFP.tif]

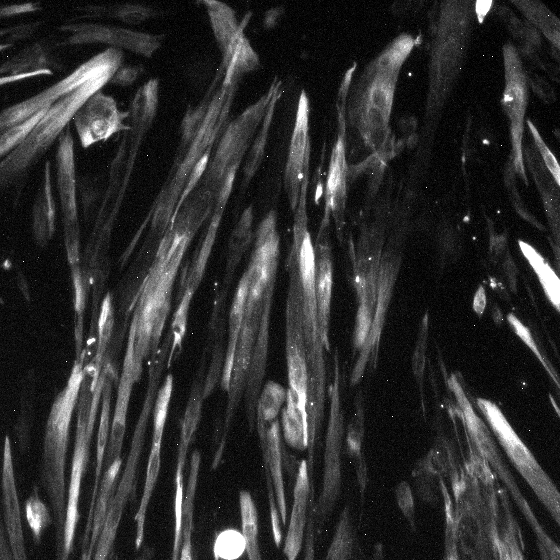

Supplement: Supplementary file 6 — Source Data for Expanded View [file MSB-18-e11129-s007.zip › Figure EV3/EV3I/Processed/MyoD1 - Desmin.tif]

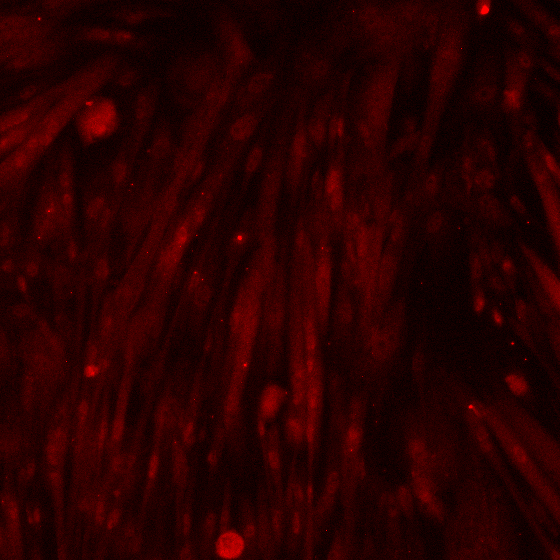

Supplement: Supplementary file 6 — Source Data for Expanded View [file MSB-18-e11129-s007.zip › Figure EV3/EV3I/Processed/MyoD1 - RFP.tif]

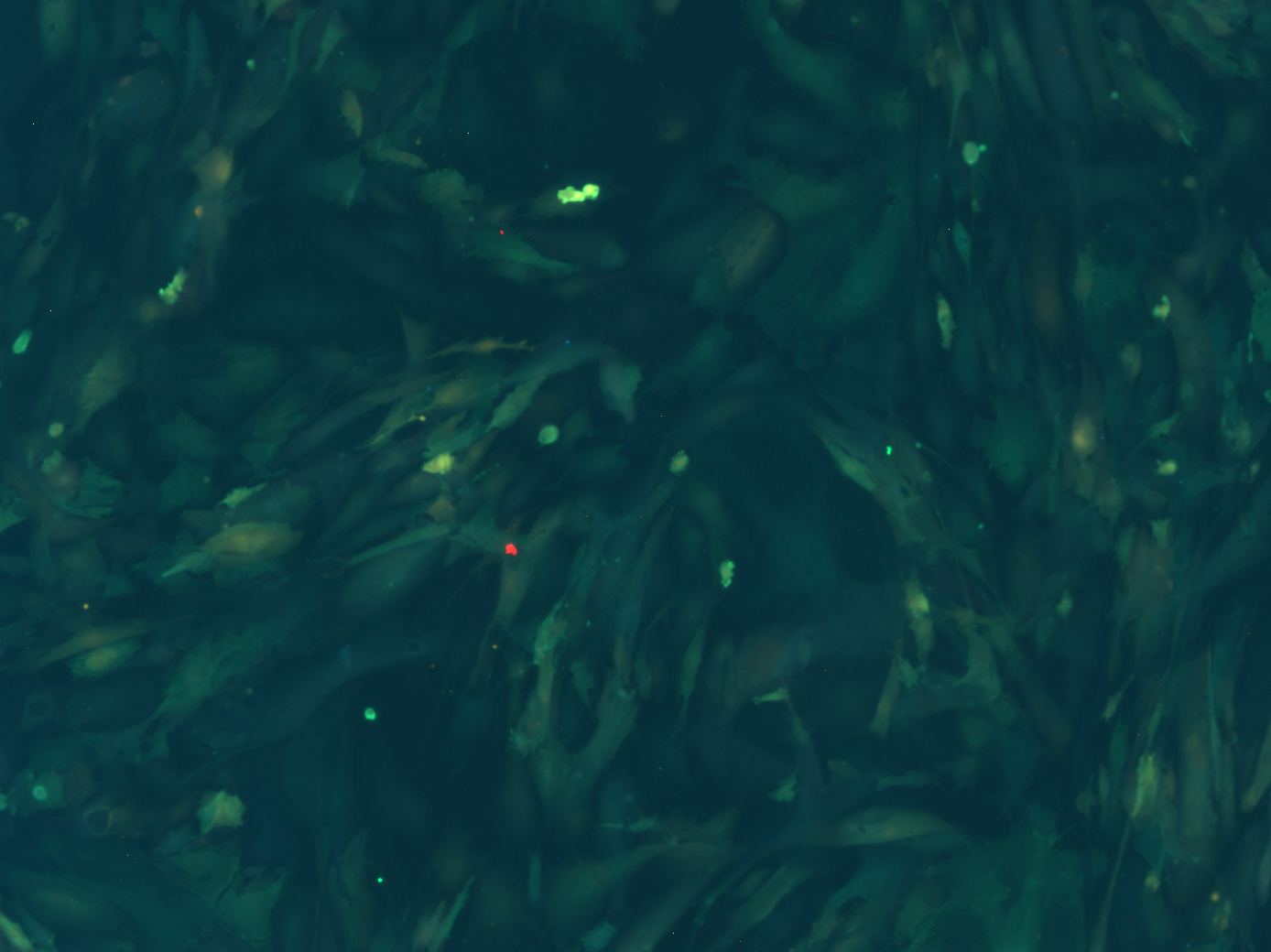

Supplement: Supplementary file 6 — Source Data for Expanded View [file MSB-18-e11129-s007.zip › Figure EV3/EV3I/Raw/Ascl1 & MyoD1.tif]

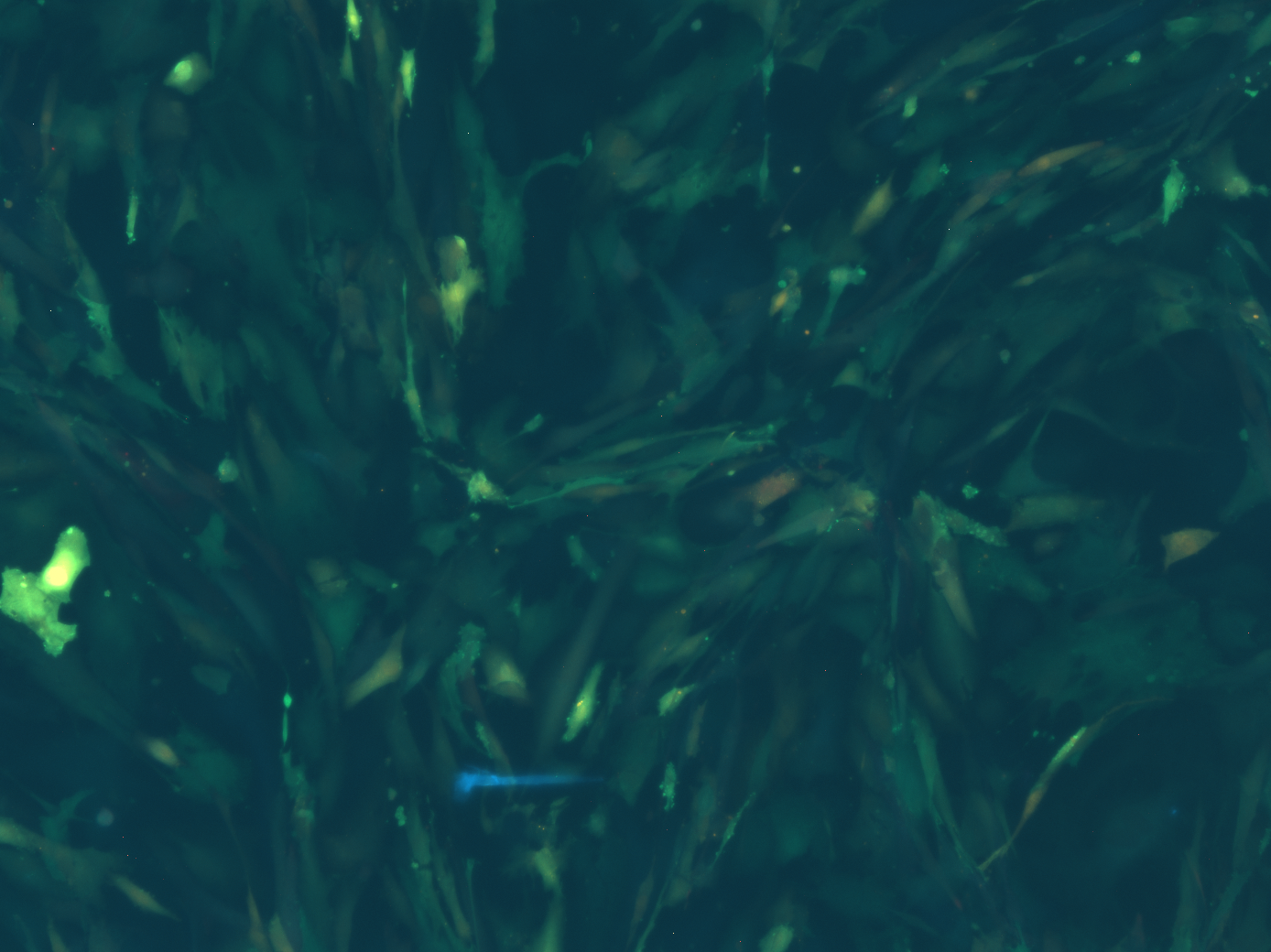

Supplement: Supplementary file 6 — Source Data for Expanded View [file MSB-18-e11129-s007.zip › Figure EV3/EV3I/Raw/mutantAscl1 & MyoD1.tif]

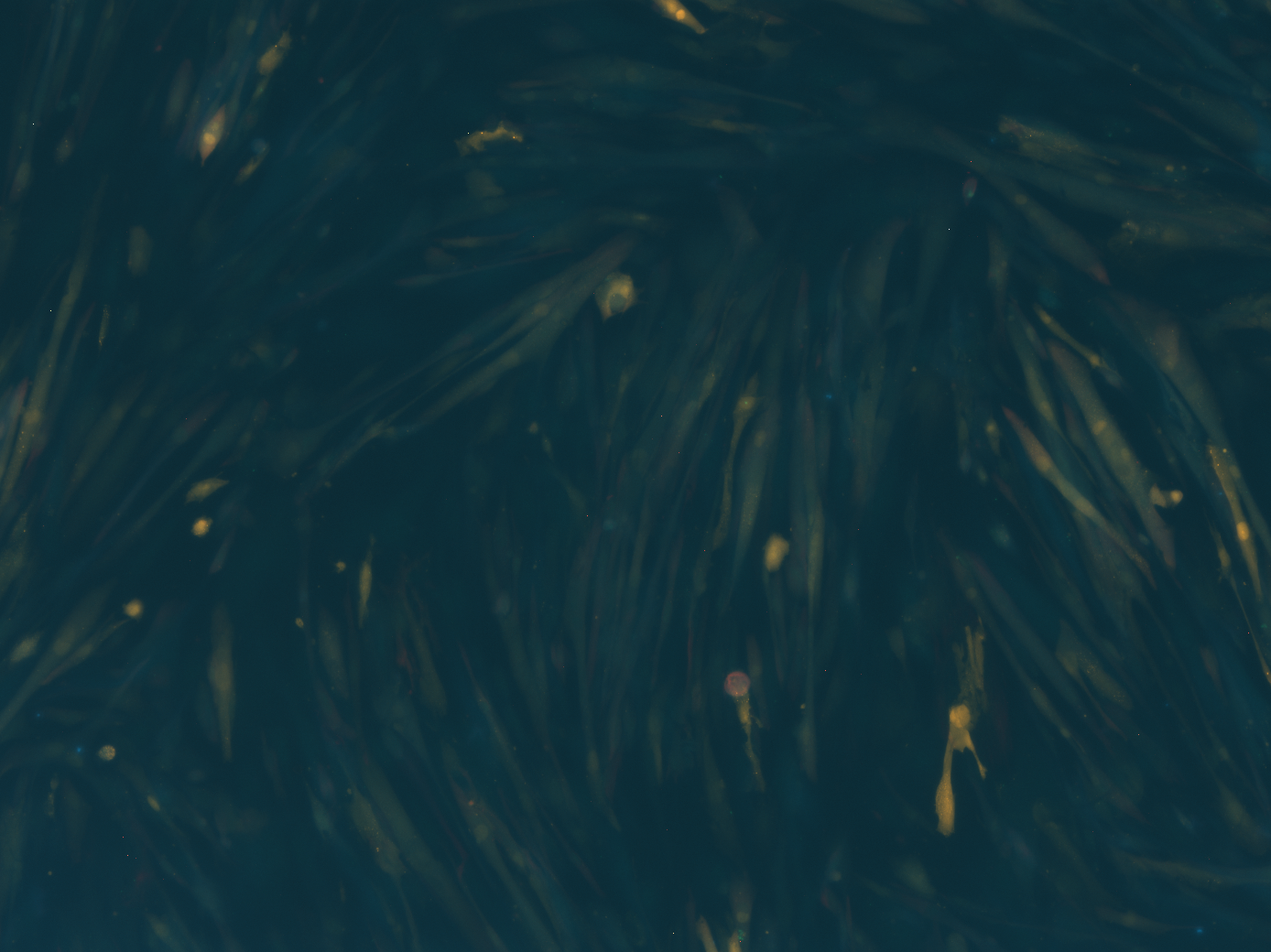

Supplement: Supplementary file 6 — Source Data for Expanded View [file MSB-18-e11129-s007.zip › Figure EV3/EV3I/Raw/MyoD1.tif]
