# Supplementary material for: Probing cell identity hierarchies by fate titration and collision during direct reprogramming
Source: Mol Syst Biol. 2022 Sep 15;18(9):e11129. doi: 10.15252/msb.202211129 (PMC9476893; doi:10.15252/msb.202211129)
Supplement: Supplementary file 5 — Source Data for Figure 5 [file MSB-18-e11129-s004.zip › SD_Figure_5/5G/Analysis notebook.html]

Analysis notebook


In [12]:

```
import pandas as pd
import seaborn as sb
import numpy as np
import matplotlib.pyplot as plt
import scipy
```

In [13]:

```
data = pd.read_csv('Data.csv',
                  sep= '\t')
```

In [14]:

```
data[data['Counter'] == 'Deeksha']
```

Out[14]:

|  | Experiment | Condition | Medium | Day | DsRed/GFP | 1 nucleus | > 1 nucleus | Percentage | Counter |
| --- | --- | --- | --- | --- | --- | --- | --- | --- | --- |
| 5 | R13 | MyoD1 | MEF | 3 | 282 | 154 | 128 | 45.390071 | Deeksha |
| 6 | R13 | Ascl1 | MEF | 3 | 229 | 221 | 8 | 3.493450 | Deeksha |
| 7 | R13 | Ascl1 + MyoD1 | MEF | 3 | 210 | 185 | 25 | 11.904762 | Deeksha |
| 8 | R13 | mutantAscl1 | MEF | 3 | 182 | 174 | 8 | 4.395604 | Deeksha |
| 9 | R13 | mutantAscl1 + MyoD1 | MEF | 3 | 129 | 123 | 6 | 4.651163 | Deeksha |
| 15 | R14 | Ascl1 | MEF | 3 | 215 | 207 | 8 | 3.720930 | Deeksha |
| 16 | R14 | MyoD1 | MEF | 3 | 204 | 178 | 26 | 12.745098 | Deeksha |
| 17 | R14 | mutantAscl1 | MEF | 3 | 199 | 183 | 16 | 8.040201 | Deeksha |
| 18 | R14 | Ascl1 + MyoD1 | MEF | 3 | 269 | 252 | 17 | 6.319703 | Deeksha |
| 19 | R14 | mutantAscl1 + MyoD1 | MEF | 3 | 205 | 196 | 9 | 4.390244 | Deeksha |
| 25 | R15 | Ascl1 | MEF | 3 | 212 | 206 | 6 | 2.830189 | Deeksha |
| 26 | R15 | MyoD1 | MEF | 3 | 196 | 145 | 51 | 26.020408 | Deeksha |
| 27 | R15 | mutantAscl1 | MEF | 3 | 190 | 179 | 11 | 5.789474 | Deeksha |
| 28 | R15 | Ascl1 + MyoD1 | MEF | 3 | 213 | 189 | 24 | 11.267606 | Deeksha |
| 29 | R15 | mutantAscl1 + MyoD1 | MEF | 3 | 201 | 176 | 25 | 12.437811 | Deeksha |
| 30 | R19 | MyoD1 | MEF | 3 | 238 | 162 | 76 | 31.932773 | Deeksha |
| 31 | R19 | mutantAscl1 | MEF | 3 | 176 | 173 | 3 | 1.704545 | Deeksha |
| 32 | R19 | Ascl1 + MyoD1 | MEF | 3 | 229 | 198 | 31 | 13.537118 | Deeksha |
| 33 | R19 | mutantAscl1 + MyoD1 | MEF | 3 | 259 | 216 | 43 | 16.602317 | Deeksha |
| 34 | R20 | Ascl1 | MEF | 3 | 271 | 271 | 0 | 0.000000 | Deeksha |
| 35 | R20 | MyoD1 | MEF | 3 | 580 | 349 | 231 | 39.800000 | Deeksha |
| 36 | R20 | mutantAscl1 | MEF | 3 | 201 | 201 | 0 | 0.000000 | Deeksha |
| 37 | R20 | Ascl1 + MyoD1 | MEF | 3 | 348 | 326 | 22 | 6.300000 | Deeksha |
| 38 | R20 | mutantAscl1 + MyoD1 | MEF | 3 | 354 | 324 | 30 | 8.500000 | Deeksha |

In [15]:

```
dataM3 = data[(data['Medium'] == "MEF") & (data['Day'] == 3) & (data['Counter'] == 'Deeksha') & (data['Experiment'] != 'R14')]
```

In [16]:

```
g = sb.catplot(
    x = 'Percentage', 
    y = 'Condition', 
    data = dataM3, 
    hue = 'Day', 
    kind = 'violin', 
    order = ['Ascl1', 'mutantAscl1','MyoD1', 'Ascl1 + MyoD1', 'mutantAscl1 + MyoD1'], 
    palette = ['lightblue'],
    legend = False,
    orient = 'h',
    height = 4
    )
g.set_xticklabels(rotation = 0, ha = 'right')
g.set_axis_labels("Percentage of cells with >1 nucleus", '')
#plt.savefig('MEF3.pdf', bbox_inches = 'tight')
```

Out[16]:

```
<seaborn.axisgrid.FacetGrid at 0x23e47b68580>
```

In [17]:

```
ax = sb.boxplot(x="Percentage", y="Condition", data=dataM3, orient = 'h', order = ['Ascl1', 'mutantAscl1','MyoD1', 'Ascl1 + MyoD1', 'mutantAscl1 + MyoD1'], palette = ['lightblue'])
ax = sb.swarmplot(x="Percentage", y="Condition", data=dataM3,color="darkslategray", order = ['Ascl1', 'mutantAscl1','MyoD1', 'Ascl1 + MyoD1', 'mutantAscl1 + MyoD1'])
ax.set(xlabel = "Percentage of cells with >1 nucleus", ylabel = '', xlim = (0,50))
#plt.savefig('MEF3_boxplot.pdf', bbox_inches = 'tight')
```

Out[17]:

```
[Text(0.5, 0, 'Percentage of cells with >1 nucleus'),
 Text(0, 0.5, ''),
 (0.0, 50.0)]
```

In [18]:

```
import scipy
MyoD1D3 = dataM3[(dataM3['Condition'] == "MyoD1")]['Percentage'].tolist()
Ascl1D3 = dataM3[(dataM3['Condition'] == "Ascl1")]['Percentage'].tolist()
mAscl1D3 = dataM3[(dataM3['Condition'] == "mutantAscl1")]['Percentage'].tolist()
Ascl1MyoD1D3 = dataM3[(dataM3['Condition'] == "Ascl1 + MyoD1")]['Percentage'].tolist()
mAscl1MyoD1D3 = dataM3[(dataM3['Condition'] == "mutantAscl1 + MyoD1")]['Percentage'].tolist()
```

In [19]:

```
a = scipy.stats.mannwhitneyu(MyoD1D3, Ascl1D3)
b =scipy.stats.mannwhitneyu(MyoD1D3, mAscl1D3)
c =scipy.stats.mannwhitneyu(MyoD1D3, Ascl1MyoD1D3)
d =scipy.stats.mannwhitneyu(MyoD1D3, mAscl1MyoD1D3)
e =scipy.stats.mannwhitneyu(Ascl1D3, Ascl1MyoD1D3)
f =scipy.stats.mannwhitneyu(Ascl1D3, mAscl1MyoD1D3)
g =scipy.stats.mannwhitneyu(mAscl1D3, Ascl1MyoD1D3)
h = scipy.stats.mannwhitneyu(mAscl1D3, mAscl1MyoD1D3)
```

In [20]:

```
pvals = [a[1], b[1], c[1], d[1], e[1], f[1], g[1], h[1]]
```

In [21]:

```
import statsmodels.stats.multitest as smt
smt.multipletests(pvals, method='fdr_bh')
```

Out[21]:

```
(array([ True,  True,  True,  True,  True,  True,  True,  True]),
 array([0.0296171 , 0.0296171 , 0.0296171 , 0.0296171 , 0.0296171 ,
        0.0296171 , 0.0296171 , 0.03030098]),
 0.006391150954545011,
 0.00625)
```
