# Supplementary material for: Probing cell identity hierarchies by fate titration and collision during direct reprogramming
Source: Mol Syst Biol. 2022 Sep 15;18(9):e11129. doi: 10.15252/msb.202211129 (PMC9476893; doi:10.15252/msb.202211129)
Supplement: Supplementary file 6 — Source Data for Expanded View [file MSB-18-e11129-s007.zip › Figure EV3/EV3H/Analysis notebook.html]

Analysis notebook


In [81]:

```
import pandas as pd
import seaborn as sns
import matplotlib.pyplot as plt
import scipy
```

In [86]:

```
ratio = pd.read_csv('Data_ratio.csv')
```

In [135]:

```
g = sns.catplot(x="Factor", 
                y="Ratio", 
                col="Target",
                data=ratio, 
                kind="bar", 
                sharey = False, 
                height=4, 
                aspect=.5, 
                ci = 95)
g.savefig('test.svg')
```

In [133]:

```
Targets = ['Hes6', 'Dll1', 'Pvalb', 'Cbfa2t3']
pvals = []
for x in test:
   pvals.append(scipy.stats.ttest_1samp(ratio[ratio['Target'] == x]['Ratio'].tolist(), 0))
```

In [134]:

```
pvals
```

Out[134]:

```
[Ttest_1sampResult(statistic=11.944086934735683, pvalue=0.006936762342491448),
 Ttest_1sampResult(statistic=8.368031541838244, pvalue=0.013982018497817621),
 Ttest_1sampResult(statistic=1.8821389846900407, pvalue=0.20053232282229647),
 Ttest_1sampResult(statistic=6.52023250951713, pvalue=0.022723303448003825)]
```
