# Supplementary material for: Probing cell identity hierarchies by fate titration and collision during direct reprogramming
Source: Mol Syst Biol. 2022 Sep 15;18(9):e11129. doi: 10.15252/msb.202211129 (PMC9476893; doi:10.15252/msb.202211129)
Supplement: Supplementary file 6 — Source Data for Expanded View [file MSB-18-e11129-s007.zip › Figure EV3/EV3J/Analysis notebook.html]

Analysis notebook


In [1]:

```
import pandas as pd
import seaborn as sb
import numpy as np
import matplotlib.pyplot as plt
```

In [2]:

```
data = pd.read_csv('Data.csv')
```

In [3]:

```
data
```

Out[3]:

|  | Label | Day | Medium | Experiment | Area | Mean | Background | Corrected mean | Normalized mean | Mode | ... | Unnamed: 14 | Unnamed: 15 | Unnamed: 16 | Unnamed: 17 | Unnamed: 18 | Unnamed: 19 | Unnamed: 20 | Unnamed: 21 | Unnamed: 22 | Unnamed: 23 |
| --- | --- | --- | --- | --- | --- | --- | --- | --- | --- | --- | --- | --- | --- | --- | --- | --- | --- | --- | --- | --- | --- |
| 0 | mutantAscl1 + MyoD1 | 3 | MEF | R13 | 118960.719 | 173.317 | 104.0908 | 69.2262 | 0.608883 | 149.0 | ... | NaN | NaN | NaN | NaN | NaN | NaN | NaN | NaN | NaN | NaN |
| 1 | Ascl1 + MyoD1 | 3 | MEF | R13 | 32816.605 | 242.505 | 161.2538 | 81.2512 | 0.714649 | 222.0 | ... | NaN | NaN | NaN | NaN | NaN | NaN | NaN | NaN | NaN | NaN |
| 2 | Ascl1 | 3 | MEF | R13 | 17450.200 | 135.146 | 80.4692 | 54.6768 | 0.480913 | 115.0 | ... | NaN | NaN | NaN | NaN | NaN | NaN | NaN | NaN | NaN | NaN |
| 3 | miRNA9\_124 + MyoD1 | 3 | MEF | R13 | 132360.728 | 249.252 | 140.7198 | 108.5322 | 0.954601 | 207.0 | ... | NaN | NaN | NaN | NaN | NaN | NaN | NaN | NaN | NaN | NaN |
| 4 | mutant Ascl1 | 3 | MEF | R13 | 9873.912 | 198.127 | 96.4378 | 101.6892 | 0.894413 | 179.0 | ... | NaN | NaN | NaN | NaN | NaN | NaN | NaN | NaN | NaN | NaN |
| 5 | MyoD1 | 3 | MEF | R13 | 209897.138 | 214.323 | 100.6292 | 113.6938 | 1.000000 | 172.0 | ... | NaN | NaN | NaN | NaN | NaN | NaN | NaN | NaN | NaN | NaN |
| 6 | MyoD1 | 3 | MEF | R15 | 194458.408 | 158.999 | 75.4880 | 83.5110 | 1.000000 | NaN | ... | NaN | NaN | NaN | NaN | NaN | NaN | NaN | NaN | NaN | NaN |
| 7 | Ascl1 + MyoD1 | 3 | MEF | R15 | 124191.167 | 104.577 | 76.2714 | 28.3056 | 0.338945 | NaN | ... | NaN | NaN | NaN | NaN | NaN | NaN | NaN | NaN | NaN |  |
| 8 | mutantAscl1 + MyoD1 | 3 | MEF | R15 | 126948.944 | 117.420 | 76.0050 | 41.4150 | 0.495923 | NaN | ... | NaN | NaN | NaN | NaN | NaN | NaN | NaN | NaN | NaN | NaN |
| 9 | MyoD1 | 3 | MEF | R14 | 184803.567 | 123.710 | 76.6906 | 47.0194 | 1.000000 | NaN | ... | NaN | NaN | NaN | NaN | NaN | NaN | NaN | NaN | NaN | NaN |
| 10 | Ascl1 + MyoD1 | 3 | MEF | R14 | 104036.647 | 114.799 | 75.7364 | 39.0626 | 0.830776 | NaN | ... | NaN | NaN | NaN | NaN | NaN | NaN | NaN | NaN | NaN | NaN |
| 11 | mutantAscl1 + MyoD1 | 3 | MEF | R14 | 87648.260 | 112.732 | 75.1800 | 37.5520 | 0.798649 | NaN | ... | NaN | NaN | NaN | NaN | NaN | NaN | NaN | NaN | NaN | NaN |

12 rows × 24 columns

In [4]:

```
data['Day'] = data['Day'].astype('object')
data.info()
```

```
<class 'pandas.core.frame.DataFrame'>
RangeIndex: 12 entries, 0 to 11
Data columns (total 24 columns):
 #   Column           Non-Null Count  Dtype  
---  ------           --------------  -----  
 0   Label            12 non-null     object 
 1   Day              12 non-null     object 
 2   Medium           12 non-null     object 
 3   Experiment       12 non-null     object 
 4   Area             12 non-null     float64
 5   Mean             12 non-null     float64
 6   Background       12 non-null     float64
 7   Corrected mean   12 non-null     float64
 8   Normalized mean  12 non-null     float64
 9   Mode             6 non-null      float64
 10  Min              12 non-null     int64  
 11  Max              12 non-null     int64  
 12  Median           6 non-null      float64
 13  Unnamed: 13      0 non-null      float64
 14  Unnamed: 14      0 non-null      float64
 15  Unnamed: 15      0 non-null      float64
 16  Unnamed: 16      0 non-null      float64
 17  Unnamed: 17      0 non-null      float64
 18  Unnamed: 18      0 non-null      float64
 19  Unnamed: 19      0 non-null      float64
 20  Unnamed: 20      0 non-null      float64
 21  Unnamed: 21      0 non-null      float64
 22  Unnamed: 22      0 non-null      float64
 23  Unnamed: 23      1 non-null      object 
dtypes: float64(17), int64(2), object(5)
memory usage: 2.4+ KB
```

In [5]:

```
dataM3 = data[(data['Medium'] == "MEF") & (data['Day'] == 3)]
```

In [6]:

```
dataM3
```

Out[6]:

|  | Label | Day | Medium | Experiment | Area | Mean | Background | Corrected mean | Normalized mean | Mode | ... | Unnamed: 14 | Unnamed: 15 | Unnamed: 16 | Unnamed: 17 | Unnamed: 18 | Unnamed: 19 | Unnamed: 20 | Unnamed: 21 | Unnamed: 22 | Unnamed: 23 |
| --- | --- | --- | --- | --- | --- | --- | --- | --- | --- | --- | --- | --- | --- | --- | --- | --- | --- | --- | --- | --- | --- |
| 0 | mutantAscl1 + MyoD1 | 3 | MEF | R13 | 118960.719 | 173.317 | 104.0908 | 69.2262 | 0.608883 | 149.0 | ... | NaN | NaN | NaN | NaN | NaN | NaN | NaN | NaN | NaN | NaN |
| 1 | Ascl1 + MyoD1 | 3 | MEF | R13 | 32816.605 | 242.505 | 161.2538 | 81.2512 | 0.714649 | 222.0 | ... | NaN | NaN | NaN | NaN | NaN | NaN | NaN | NaN | NaN | NaN |
| 2 | Ascl1 | 3 | MEF | R13 | 17450.200 | 135.146 | 80.4692 | 54.6768 | 0.480913 | 115.0 | ... | NaN | NaN | NaN | NaN | NaN | NaN | NaN | NaN | NaN | NaN |
| 3 | miRNA9\_124 + MyoD1 | 3 | MEF | R13 | 132360.728 | 249.252 | 140.7198 | 108.5322 | 0.954601 | 207.0 | ... | NaN | NaN | NaN | NaN | NaN | NaN | NaN | NaN | NaN | NaN |
| 4 | mutant Ascl1 | 3 | MEF | R13 | 9873.912 | 198.127 | 96.4378 | 101.6892 | 0.894413 | 179.0 | ... | NaN | NaN | NaN | NaN | NaN | NaN | NaN | NaN | NaN | NaN |
| 5 | MyoD1 | 3 | MEF | R13 | 209897.138 | 214.323 | 100.6292 | 113.6938 | 1.000000 | 172.0 | ... | NaN | NaN | NaN | NaN | NaN | NaN | NaN | NaN | NaN | NaN |
| 6 | MyoD1 | 3 | MEF | R15 | 194458.408 | 158.999 | 75.4880 | 83.5110 | 1.000000 | NaN | ... | NaN | NaN | NaN | NaN | NaN | NaN | NaN | NaN | NaN | NaN |
| 7 | Ascl1 + MyoD1 | 3 | MEF | R15 | 124191.167 | 104.577 | 76.2714 | 28.3056 | 0.338945 | NaN | ... | NaN | NaN | NaN | NaN | NaN | NaN | NaN | NaN | NaN |  |
| 8 | mutantAscl1 + MyoD1 | 3 | MEF | R15 | 126948.944 | 117.420 | 76.0050 | 41.4150 | 0.495923 | NaN | ... | NaN | NaN | NaN | NaN | NaN | NaN | NaN | NaN | NaN | NaN |
| 9 | MyoD1 | 3 | MEF | R14 | 184803.567 | 123.710 | 76.6906 | 47.0194 | 1.000000 | NaN | ... | NaN | NaN | NaN | NaN | NaN | NaN | NaN | NaN | NaN | NaN |
| 10 | Ascl1 + MyoD1 | 3 | MEF | R14 | 104036.647 | 114.799 | 75.7364 | 39.0626 | 0.830776 | NaN | ... | NaN | NaN | NaN | NaN | NaN | NaN | NaN | NaN | NaN | NaN |
| 11 | mutantAscl1 + MyoD1 | 3 | MEF | R14 | 87648.260 | 112.732 | 75.1800 | 37.5520 | 0.798649 | NaN | ... | NaN | NaN | NaN | NaN | NaN | NaN | NaN | NaN | NaN | NaN |

12 rows × 24 columns

In [7]:

```
g = sb.catplot(
    x = 'Normalized mean', 
    y = 'Label', 
    data = dataM3, 
    hue = 'Experiment', 
    order = ['MyoD1', 'Ascl1 + MyoD1', 'mutantAscl1 + MyoD1'], 
    palette = 'tab10',
    legend = False,
    orient = 'h',
    height = 4
    )
g.set_xticklabels(rotation = 0)
g.set_axis_labels("Mean Desmin intensity (a.u.)", '')
plt.savefig('MEF3.svg', bbox_inches = 'tight')
```

In [8]:

```
ax = sb.boxplot(x="Normalized mean", y="Label", data=dataM3, orient = 'h', order = ['MyoD1', 'Ascl1 + MyoD1', 'mutantAscl1 + MyoD1'], palette = ['steelblue'])
ax = sb.swarmplot(x="Normalized mean", y="Label", data=dataM3, hue = 'Experiment',color=".25", order = ['MyoD1', 'Ascl1 + MyoD1', 'mutantAscl1 + MyoD1'], palette = 'tab10')
ax.set(xlabel = "Mean Desmin intensity (a.u.)", ylabel = '', xlim = (0,1))
plt.legend(loc='upper left')
plt.savefig('MEF3_boxplot.svg', bbox_inches = 'tight')
```

In [9]:

```
MyoD1D3 = dataM3[(dataM3['Label'] == "MyoD1")]['Normalized mean'].tolist()
Ascl1MyoD1D3 = dataM3[(dataM3['Label'] == "Ascl1 + MyoD1")]['Normalized mean'].tolist()
mAscl1MyoD1D3 = dataM3[(dataM3['Label'] == "mutantAscl1 + MyoD1")]['Normalized mean'].tolist()
```

In [10]:

```
import scipy
a =scipy.stats.mannwhitneyu(MyoD1D3, Ascl1MyoD1D3)
b =scipy.stats.mannwhitneyu(MyoD1D3, mAscl1MyoD1D3)
c =scipy.stats.mannwhitneyu(Ascl1MyoD1D3, mAscl1MyoD1D3)
pvals = [a[1], b[1], c[1]]
```

In [11]:

```
import statsmodels.stats.multitest as smt
smt.multipletests(pvals, method='fdr_bh')
```

Out[11]:

```
(array([ True,  True, False]),
 array([0.04770193, 0.04770193, 0.5       ]),
 0.016952427508441503,
 0.016666666666666666)
```
