# Supplementary material for: Probing cell identity hierarchies by fate titration and collision during direct reprogramming
Source: Mol Syst Biol. 2022 Sep 15;18(9):e11129. doi: 10.15252/msb.202211129 (PMC9476893; doi:10.15252/msb.202211129)
Supplement: Supplementary file 6 — Source Data for Expanded View [file MSB-18-e11129-s007.zip › Figure EV1/EV1A/Analysis notebook.html]

Analysis Notebook


In [4]:

```
import pandas as pd
import matplotlib.pyplot as plt
import seaborn as sb
```

In [5]:

```
data = pd.read_csv('Data.csv', sep = ',')
```

In [6]:

```
data
```

Out[6]:

|  | Cell batch | Target | Fold change | SD | Comment |
| --- | --- | --- | --- | --- | --- |
| 0 | 1 | Ascl1 | 202.842812 | 14.862166 | NaN |
| 1 | 1 | MyoD1 | 404.809964 | 1.468405 | NaN |
| 2 | 1 | Hnf1a | 103.257457 | 7.438253 | NaN |
| 3 | 1 | Oct4 | 187.961391 | 11.692310 | NaN |
| 4 | 2 | Ascl1 | 160.554408 | 38.864367 | NaN |
| 5 | 2 | MyoD1 | 369.000000 | 18.073850 | NaN |
| 6 | 2 | Hnf1a | 105.000000 | 10.008265 | NaN |
| 7 | 2 | Oct4 | 170.752060 | 38.117146 | NaN |
| 8 | 3 | Ascl1 | 172.345831 | 32.226090 | Round 5 |
| 9 | 3 | MyoD1 | 456.489642 | 79.750632 | Round 5 |
| 10 | 3 | Hnf1a | 127.361180 | 18.985387 | Round 5 |
| 11 | 3 | Oct4 | 160.732454 | 60.967315 | Round 5 |
| 12 | 4 | FoxA2 | 494.304935 | 128.722726 | ARE |
| 13 | 4 | Sox2 | 47.874481 | 22.036753 | ARE |
| 14 | 5 | FoxA2 | 406.170042 | 104.638915 | ARE |
| 15 | 5 | Sox2 | 85.874959 | 11.334791 | ARE |

In [7]:

```
ax = sb.barplot(x="Target", y="Fold change", data=data, order = ['Ascl1', 'MyoD1', 'FoxA2', 'Sox2', 'Oct4', 'Hnf1a'])
ax = sb.stripplot(x="Target", y="Fold change", data=data, order = ['Ascl1', 'MyoD1', 'FoxA2', 'Sox2', 'Oct4', 'Hnf1a'], color=".3")
```

In [ ]:

```

```
