# Supplementary material for: Probing cell identity hierarchies by fate titration and collision during direct reprogramming
Source: Mol Syst Biol. 2022 Sep 15;18(9):e11129. doi: 10.15252/msb.202211129 (PMC9476893; doi:10.15252/msb.202211129)
Supplement: Supplementary file 7 [file MSB-18-e11129-s005.pdf]

molecular systems biology

# Probing cell identity hierarchies by fate titration and collision during direct reprogramming

Bob Hersbach, David Fischer, Giacomo Masserdotti, Deeksha -, Karolina Mojžišová, Thomas Walzthoeni, Diego Rodriguez-Terrones, Matthias Heinig, Fabian Theis, Magdalena Götz, and Stefan Stricker
**DOI: 10.15252/msb.202211129**

*Corresponding author(s): Stefan Stricker (stefan.stricker@helmholtz-muenchen.de) , Fabian Theis (Fabian.theis@helmholtz-muenchen.de), Magdalena Götz (magdalena.goetz@helmholtz-muenchen.de)*



## Review Timeline:

| | |
|---|---|
| Submission Date: | 19th May 22 |
| Editorial Decision: | 27th Jun 22 |
| Revision Received: | 1st Aug 22 |
| Accepted: | 17th Aug 22 |

*Editor: Maria Polychronidou*


## Transaction Report:

(Note: With the exception of the correction of typographical or spelling errors that could be a source of ambiguity, letters and reports are not edited. Depending on transfer agreements, referee reports obtained elsewhere may or may not be included in this compilation. Referee reports are anonymous unless the Referee chooses to sign their reports.)

***The reviewers' comments and authors' responses are not available with this article, as the initial review process took place with another journal.***

## 1st Editorial Decision 27th Jun 2022

RE: MSB-2022-11129, Probing cell identity hierarchies by fate titration and collision during direct reprogramming

Thank you again for submitting your work to Molecular Systems Biology. We have now heard back from the two reviewers who agreed to evaluate your study. Reviewer #1 is the same reviewer #1 who evaluated the initial version of the study at the other journal. Reviewer #2 is a new reviewer, who was asked to focus on the methodological and computational aspects of the study as we felt that the three reviewers at the other journal did not comment much on these. Both reviewers were asked to evaluate whether the previously raised concerns have been adequately addressed, and to assess the suitability of the revised study for publication keeping in mind the editorial criteria of Molecular Systems Biology.

As you will see below, reviewer #2 is overall supportive, but lists a series of concerns, which we think are straightforward to address. On the other hand, reviewer #1 mentions that some of the previously raised concerns remain not fully addressed. They state that they would have preferred the inclusion of additional experiments to address these concerns, besides the provided explanations and clarifications. These comments seem to mostly refer to the recommendations of the previous reviewers to provide more in-depth mechanistic insights. As we had discussed previously, our editorial view is that further mechanistic analyses do not seem required for publication in Molecular Systems Biology. On balance, given that the reviewers find the study of potentially high interest for the field and they do not raise technical concerns that would challenge the quality of the work, we would in principle be in favor of publishing the study, pending addressing the issues raised by reviewer #2. As such, we would invite you to submit a revision. I would like to mention that if in the meantime you have experimental data addressing (some of) the previously raised issues related to the mechanisms of cell fate conversion we would encourage you to include them.

In your revision, we would also ask you to address the following editorial issues:

Reviewer #1:

I have already reviewed this manuscript and expressed a number of concerns and suggestions. The authors have decided to address these concerns verbally, without performing all the experiments recommended (not only by this reviewer, but also by the other reviewers). As such, while the manuscript remains of potential high interest, I reiterate the same concerns previously expressed as issues that currently limit the interest of this work. An effort to clarify the points raised, even with representative experiments (rather than genome-wide analyses) are, again, highly recommended.

Reviewer #2:

Summary:
The study describes an approach, Collide-seq, to assess the transcriptomic consequences of the simultaneous induction of multiple reprogramming factors. The authors address a number of fundamental questions using this system including: (1) Do different factors suppress starting cell type identity in different manners? (2) Do additional factors have a synergistic or antagonistic transcriptomic effect on loss of starting cell type identity and subsequent fate? (3) Elucidate how the over-expression of multiple factors follows a multi-stable state model in which distinct collision states can be reached. Additionally, the authors explore in depth the MyoD1 and Ascl1 "collision" demonstrating that the resulting antagonistic effects are not binding-dependent.

Comments:

1) In the introduction it would be helpful to more clearly define "cell identity conflict". Is this specifically describing a transcriptional phenomenon where more than one program is activated (e.g. like a hybrid state?)?

2) Figure 2B and corresponding text: A minor point, the coloring is a little confusing here. For example, the text says "the fibroblast population showed four clusters, three of which were close to each other," but it seems like there may be 5 clusters here? (three together at the bottom, one at the very left side of the UMAP, and a small cluster just underneath the red cluster

3) Figure 3A and corresponding text describing differential expression analysis: It would be helpful if 'overall differential expression magnitude' could be defined-I couldn't find an explanation for this value in the 'Differential Expression' section of the methods and also didn't see it in the figure legend (which refers back to the methods), but am assuming it is the sum of the absolute log-fold change of each gene in the expression data.

4) Figure 4A and corresponding text (i.e. the claim "the fact that significant synergistic effects were only seen for Sox2 and Ascl1 indicates that the transcriptomic interactions follow physiological principles, as both factors are expressed in neuronal progenitor cells."): This is not well supported by the data presented. The combination of Sox2+Oct4 does not significantly depress fibroblast score (supp. Figure 6A). Actually, based on Figure 3F, Sox2 and Ascl1 have the smallest overlap of downregulated genes - the synergistic effect could just be from the two downregulating two separate sets of fibroblast-related genes and not because they are both expressed in NPCs.

5) Figure 4B and corresponding text: I couldn't find how pseudobulk samples were created (there is reference to the Methods, but I couldn't find the information there). The text suggests that all cells within a condition were collapsed into a single sample, but this seems as if it would cause some significant masking considering the multi-stable state system that exists (and that is further described in the rest of the paper).

6) Figure 5B and corresponding text: The text/clustering suggests the Ascl1+Myod1 combination results in 2 distinct collision states. In the previous collide-seq (Figure 4), the analysis (e.g. 'FTA') doesn't seem to suggest there are two distinct states, why is this the case? Additionally some textual clarification could be helpful in the section, especially referring to the distinct states as they are labeled in the figure ('Ascl1-like collision state', 'Intermediate MyoD1 fate', etc.).

7) In the discussion, in reference to "MyoD1 was most potent in imposing its fate.... This came as a surprise...": It could be useful to add discussion that of the reprogramming factors used here, MyoD1 is the only one (as far as I'm aware) that can successfully reprogram fibroblasts alone. The other factors have to be used in combination with other factors not included in these collide-seq runs for successful reprogramming. For that reason, it's not all that surprising that MyoD1 is the most 'potent'.

8) In the discussion, in reference to "Interestingly, the resulting transcriptomic states were found to be a non-linear function.... ("binary switch").": This observation resembles the multi-state systems that exist in development and other fate decisions. It could be worth describing similar "switch" systems that have been described before (e.g. systems where over-expression of

each TF leads to specific cell fates, but a 'balance' of them results in a distinct state). For example, the bipotent decision in erythroid vs. megakaryocyte fate (PMID: 17412320) or the "seesaw model" of pluripotency and lineage specification (PMID: 23706735).

**Point-By-Point response**

**Reviewer comments**

*1) In the introduction it would be helpful to more clearly define "cell identity conflict". Is this specifically describing a transcriptional phenomenon where more than one program is activated (e.g. like a hybrid state?)?*

**We would like to thank the reviewer for pointing out this ambiguity. We now more clearly explain cell identity conflicts in the introduction and emphasize how little is known about their molecular basis. We also more clearly specified the questions we address within this context and write:**

"Finally, another widely overlooked aspect of cell fate conversion is that it always entails a cell identity conflict, i.e., the existing cell identity gets challenged by a reprogramming transcription factor attempting to induce a conflicting program. We know, however, very little about the molecular basis of cell identity conflicts, particularly on which molecular level they collide and how cells resolve such conflicts. For example, can mismatched transcriptional programs be present simultaneously for a significant amount of time or does competition for co-factors and/or binding interfere with the full implementation of programs?

*2) Figure 2B and corresponding text: A minor point, the colouring is a little confusing here. For example, the text says "the fibroblast population showed four clusters, three of which were close to each other," but it seems like there may be 5 clusters here? (three together at the bottom, one at the very left side of the UMAP, and a small cluster just underneath the red cluster.*

**The k-nearest neighbour graph clustering did indeed yield four clusters derived from control fibroblast transcriptomes. However, one of them breaks up in into two groups in the UMAP (2D embedding). This is an artefact of projecting the complex data manifold into two dimensions, a known limitation of UMAP and other projection methods (Chari *et al*, 2021). Transcriptomically the fibroblasts are very similar, which is captured by them being attributed to the same cluster.**

*3) Figure 3A and corresponding text describing differential expression analysis: It would be helpful if 'overall differential expression magnitude' could be defined-I couldn't find an explanation for this value in the 'Differential Expression' section of the methods and also didn't see it in the figure legend (which refers back to the methods), but am assuming it is the sum of the absolute log-fold change of each gene in the expression data.*

**We would like to thank the reviewer for pointing out this shortcoming. We indeed failed to clearly define this term in the methods so far. We have now updated the methods and write:**

**"**We defined differential expression magnitude as the L2 norm over all gene-wise parameter estimators for an individual coefficient defined in the model, only using genes for which this coefficient was significant (FDR-corrected p-values < 0.01) and which were likely not overfitted (defined as a L1 norm of significant effects within a gene < 100). **"**

*4) Figure 4A and corresponding text (i.e. the claim "the fact that significant synergistic effects were only seen for Sox2 and Ascl1 indicates that the transcriptomic interactions follow physiological principles, as both factors are expressed in neuronal progenitor cells."): This is not well supported by the data presented. The combination of Sox2+Oct4 does not significantly depress fibroblast score (supp. Figure 6A). Actually, based on Figure 3F, Sox2 and Ascl1 have the smallest overlap of downregulated genes - the synergistic effect could just be from the two downregulating two separate sets of fibroblast-related genes and not because they are both expressed in NPCs.*

**We would like to thank the reviewer for the constructive criticism. We agree that relating the Ascl1 & Sox2 data to "physiological principles" might be an overstatement, since other physiological pairs such as Oct4 & Sox2 did not show such behaviour. We have now removed this statement from the text and write:**

"For instance, the combination of Ascl1 and Sox2 repressed fibroblast identity more efficiently than Ascl1 alone (**Figure 4A**). In contrast, cells expressing Ascl1 and MyoD1 maintained a much higher fibroblast score than those expressing MyoD1 alone, while neither Sox2 nor FoxA2 had such a negative impact on MyoD1 (**Figure 4A**). Furthermore, the addition of Sox2 and Oct4 did not improve identity loss for any of the other factors, suggesting that these pluripotency genes do not act as general enablers by facilitating starter cell identity removal, as has been suggested before (Deleidi et al, 2011; Kim et al, 2011; Peskova et al, 2019; Sharma et al, 2019). This reinforces the concept that different molecular mechanisms for cell identity removal exist and demonstrates that these mechanisms can act synergistically in some instances, yet antagonistic or neutral outcomes are more common (**Figure 4A, Figure EV2A).**

*5) Figure 4B and corresponding text: I couldn't find how pseudobulk samples were created (there is reference to the Methods, but I couldn't find the information there). The text suggests that all cells within a condition were collapsed into a single sample, but this seems as if it would cause some significant masking considering the multi-stable state system that exists (and that is further described in the rest of the paper).*

**We would like to thank the reviewer for pointing out this shortcoming. We have now clarified that we take the mean over cells of each condition in the main text. The mean can indeed potentially mask multiple modes in the distribution. However, the correspondence of inferred genetic conditions to transcriptomic clusters shows that variation between condition is well captured by transcriptomic summaries. Thus, for the purpose of comparing global variation, we believe that the mean is a suitable statistic here. Note that this analysis specifically serves the purpose of demonstrating that the transcriptomic variation over conditions follows reasonable patterns, thus, complementing the UMAP, which may distort relations between clusters. In the subsequent analysis and figures, we dig deeper into the variation within conditions. In the main text, we now write the following:**

**"**For this, we first collapsed the cells within each condition to their mean, forming pseudobulk samples, and performed hierarchical clustering (**Figure EV2B**).

*6) Figure 5B and corresponding text: The text/clustering suggests the Ascl1+Myod1 combination results in 2 distinct collision states. In the previous collide-seq (Figure 4), the analysis (e.g. 'FTA') doesn't seem to suggest there are two distinct states, why is this the case? Additionally some textual clarification could be helpful in the section, especially referring to the distinct states as they are labeled in the figure ('Ascl1-like collision state', 'Intermediate MyoD1 fate', etc.).*

**The first Collide-seq experiment presented in the paper gives a general overview of a relatively rich collision space between five factors at 72h after induction. In the second dataset, we do not only effectively sample more cells in this area by choosing fewer and less potent factors next to Ascl1 and MyoD1, but also by sampling in a time series, at 24h, 48h, and 72h after induction. In this way, we gain more power to resolve subtle variations in the collision state and do indeed find meaningful sub-clusters that we can relate back to transgene induction strength, as shown by the FTA in Fig. 5. Here, we label the sub-clusters based on the relative expression of both transgenes and their relative proximity the respective single-positive lineage end-points identified at 72h (EV Fig 4G). We rephrased this in the main text and write now:**

“Using the large number of cells within the Ascl1 and MyoD1 lineages, together with the increased temporal resolution obtained by sampling at different timepoints (24h, 48h & 72h), we were able to stratify the previously identified collision states into two Louvain clusters (**Figure 5B**). Importantly, these transcriptomic states did not occur in the single transgene conditions and the predicted lineage endpoints were distinct from the single positive Ascl1 and MyoD1 endpoints (**Figure 5B**).

“Furthermore, based on their overall similarity to the respective single factor lineages, one collision state was found to be more similar to the Ascl1 program while the other was more similar to the MyoD1 program (Ascl1-like intermediate, MyoD1-like intermediate, **Figure EV3G**).”

*7) In the discussion, in reference to "MyoD1 was most potent in imposing its fate.... This came as a surprise...": It could be useful to add discussion that of the reprogramming factors used here, MyoD1 is the only one (as far as I'm aware) that can successfully reprogram fibroblasts alone. The other factors have to be used in combination with other factors not included in these collide-seq runs for successful reprogramming. For that reason, it's not all that surprising that MyoD1 is the most 'potent'.*

**We would like to thank the reviewer for raising this point. The reviewer is correct that MyoD1, on its own, is used to reprogram fibroblasts. However, among the other factors, Ascl1, FoxA2, Sox2 and Oct4 have also been shown to drive fibroblast reprogramming by themselves (See PMID: 25254342 PMID: 28757167, PMID: 22683203, PMID: 20956998) Moreover, all factors are considered pioneer factors and key drivers of their lineage. Therefore, we expected a similar ability of these factors to regulate their primary targets. We have now extended the discussion in this context and write:**

“Strikingly, we found that the mesodermal factor MyoD1 was most potent in imposing its fate as well as repressing the original mesodermal fibroblast identity. This might seem surprising, since not only MyoD1 but also Ascl1, FoxA2, Sox2 and Oct4 have been attributed pioneer factor activity and are all considered key drivers of their respective reprogramming paradigms (Iwafuchi-Doi & Zaret, 2014; Zaret & Mango, 2016; Zaret, 2020; Sunkel & Stanton, 2021). Indeed, Ascl1 and MyoD1 are, on their own, sufficient to reprogram MEFs into induced neurons and muscles respectively (Chanda et al, 2014; Davis et al, 1987). Additionally, Sox2 and Oct4 have been reported to individually reprogram MEFs into neural progenitor cells and IPSCs as well , albeit under specialized media conditions (Ring et al, 2012; Li et al, 2011). As such, we expected a similar ability between these factors to regulate their primary targets. Moreover, we predicted that non-mesodermal factors would silence more fibroblast genes due to larger differences in the active gene regulatory networks between starter and target cell. Instead, we find that non-mesodermal factors downregulate fewer genes as compared to the mesodermal factor MyoD1, suggesting that a special relationship between starter cells and reprogramming factors exists, possibly related to germ layer identity. In line with this notion is MyoD1’s inefficiency to transdifferentiate cells into myocytes when a starter cell of non-mesodermal origin is used, indicating that MyoD1’s potency might be indeed dependent on the cell of origin (Davis et al, 1987; Weintraub et al, 1989). Taken together, our results support the hypothesis that transdifferentiation into more closely related cell types might be more thorough and/or efficient (Hochedlinger & Plath, 2009; Morris & Daley, 2013). Therefore, therapeutic cell conversions may best be limited to related cell types if possible (Morris & Daley, 2013).

*8) In the discussion, in reference to "Interestingly, the resulting transcriptomic states were found to be a non-linear function.... ("binary switch").": This observation resembles the multi-state systems that exist in development and other fate decisions. It could be worth describing similar "switch" systems that have been described before (e.g. systems where over-expression of each TF leads to specific cell fates, but a 'balance' of them results in a distinct state). For example, the bipotent decision in*

*erythroid vs. megakaryocyte fate (PMID: 17412320) or the "seesaw model" of pluripotency and lineage specification (PMID: 23706735).*

**We would like to thank the reviewer for this excellent suggestion. There are indeed significant similarities between these two models and our findings. We have now incorporated this in the discussion and write:**

“Interestingly, the strong reprogramming ability of MyoD1 on its own did not hold in competitive conditions. We discovered this surprising sensitivity of the pioneer factor through simultaneous induction of multiple divergent fates. MyoD1’s program was effectively perturbed by all other factors, except Oct4, and this effect was largely independent of the expression levels of MyoD1 and of the colliding factors (Figure 4E). Although we generally found antagonistic effects of colliding factors more frequently than synergism, one of the factors tested, Ascl1, was particularly disruptive and the only one capable of hindering MyoD1’s potential to erase the fibroblast identity (Figure 4A). To our knowledge, these experiments are the first to systematically position different reprogramming factors against each other. Some of our findings are, however, reminiscent of the previously proposed ‘seesaw model’ (Shu et al, 2013) in which opposing lineage factors are thought to cancel each other out, stabilizing a pluripotent stem cell identity.”

“We also characterized transcription factor abundance-dependent fate choices with fate titration analysis, showing that Collide-seq can be used to titrate transcription factors against each other to screen fate outcomes. Interestingly, the resulting transcriptomic states were found to be a non-linear function of the reprogramming factor expression levels, which is both highlighted by the emergence of collision states in fate titration analyses as well as the step-like saturating dependency of gene activation of transcription factor induction (“binary switch”). In other words, cell fate conversion does not scale gradually with increasing reprogramming factor expression levels but instead depends on whether a critical expression threshold is reached. This observation is highly reminiscent of a previously described switch system in blood cell differentiation, where transcriptomic changes corresponding to cell fate decisions also occur abruptly rather than gradually (Huang et al, 2007). Taken together, this finding indicates that an optimal level of reprogramming factors expression exists, which is sufficient for reprogramming but also minimizes the risk of failure due to cellular stress as a result of transcription factor overexpression. Thus, identifying optimal reprogramming factor expression levels could significantly improve existing reprogramming protocols. Furthermore, the cell-wise perturbation effects described here may also be leveraged for gene regulatory network modelling, as they tend to be stronger than available data based on single-cell knock out data. Hence, they may facilitate the development of bottom-up models for fate collision on the chromatin and transcriptome level in the future (Kamimoto et al, 2020).”

**References**

Chari T, Banerjee J & Pachter L (2021) The Specious Art of Single-Cell Genomics. *bioRxiv*: 2021.08.25.457696

## 1st Revision - Editorial Decision 17th Aug 2022

Manuscript number: MSB-2022-11129R
Title: Probing cell identity hierarchies by fate titration and collision during direct reprogramming

Thank you again for sending us your revised manuscript. We are now satisfied with the modifications made and I am pleased to inform you that your paper has been accepted for publication.

## EMBO Press Author Checklist

| | |
|---|---|
| Corresponding Author Name: Prof. Dr. Stefan H. Stricker | |
| Journal Submitted to: EMBO Molecular Systems Biology | |
| Manuscript Number: #MSB-2022-11129 | |

**USEFUL LINKS FOR COMPLETING THIS FORM**
The EMBO Journal - Author Guidelines
EMBO Reports - Author Guidelines
Molecular Systems Biology - Author Guidelines
EMBO Molecular Medicine - Author Guidelines

**Reporting Checklist for Life Science Articles (updated January**
This checklist is adapted from Materials Design Analysis Reporting (MDAR) Checklist for Authors. MDAR establishes a minimum set of requirements in transparent reporting in the life sciences (see Statement of Task: 10.31222/osf.io/9sm4x). Please follow the journal's guidelines in preparing your
**Please note that a copy of this checklist will be published alongside your article.**

**Abridged guidelines for figures**
**1. Data**
The data shown in figures should satisfy the following conditions:
- the data were obtained and processed according to the field's best practice and are presented to reflect the results of the experiments in an accurate and unbiased manner.
- ideally, figure panels should include only measurements that are directly comparable to each other and obtained with the same assay.
- plots include clearly labeled error bars for independent experiments and sample sizes. Unless justified, error bars should not be shown for technical
- if n<5, the individual data points from each experiment should be plotted. Any statistical test employed should be justified.
- Source Data should be included to report the data underlying figures according to the guidelines set out in the authorship guidelines on Data

**2. Captions**
Each figure caption should contain the following information, for each panel where they are relevant:
- a specification of the experimental system investigated (eg cell line, species name).
- the assay(s) and method(s) used to carry out the reported observations and measurements.
- an explicit mention of the biological and chemical entity(ies) that are being measured.
- an explicit mention of the biological and chemical entity(ies) that are altered/varied/perturbed in a controlled manner.
- the exact sample size (n) for each experimental group/condition, given as a number, not a range;
- a description of the sample collection allowing the reader to understand whether the samples represent technical or biological replicates (including how many animals, litters, cultures, etc.).
- a statement of how many times the experiment shown was independently replicated in the laboratory.
- definitions of statistical methods and measures:
  - common tests, such as t-test (please specify whether paired vs. unpaired), simple χ2 tests, Wilcoxon and Mann-Whitney tests, can be unambiguously identified by name only, but more complex techniques should be described in the methods section;
  - are tests one-sided or two-sided?
  - are there adjustments for multiple comparisons?
  - exact statistical test results, e.g., P values = x but not P values < x;
  - definition of 'center values' as median or average;
  - definition of error bars as s.d. or s.e.m.

**Please complete ALL of the questions below.**
**Select "Not Applicable" only when the requested information is not relevant for your study.**

**Materials**

| Newly Created Materials | Information included in the manuscript? | In which section is the information available? (Reagents and Tools Table, Materials and Methods, Figures, Data Availability Section) |
|---|---|---|
| New materials and reagents need to be available; do any restrictions apply? | Not Applicable | |

| Antibodies | Information included in the manuscript? | In which section is the information available? (Reagents and Tools Table, Materials and Methods, Figures, Data Availability Section) |
|---|---|---|
| For **antibodies** provide the following information:<br>- Commercial antibodies: RRID (if possible) or supplier name, catalogue number and or/clone number<br>- Non-commercial: RRID or citation | Yes | Appendix Table S3 |

| DNA and RNA sequences | Information included in the manuscript? | In which section is the information available? (Reagents and Tools Table, Materials and Methods, Figures, Data Availability Section) |
|---|---|---|
| **Short novel DNA or RNA including primers, probes**: provide the sequences. | Yes | Appendix Table S4 |

| Cell materials | Information included in the manuscript? | In which section is the information available? (Reagents and Tools Table, Materials and Methods, Figures, Data Availability Section) |
|---|---|---|
| **Cell lines:** Provide species information, strain. Provide accession number in repository **OR** supplier name, catalog number, clone number, and/**OR** RRID. | Not Applicable | |
| **Primary cultures:** Provide species, strain, sex of origin, genetic modification status. | Yes | Materials and Methods |
| Report if the cell lines were recently **authenticated** (e.g., by STR profiling) and tested for mycoplasma contamination. | Not Applicable | |

| Experimental animals | Information included in the manuscript? | In which section is the information available? (Reagents and Tools Table, Materials and Methods, Figures, Data Availability Section) |
|---|---|---|
| **Laboratory animals or Model organisms:** Provide species, strain, sex, age, genetic modification status. Provide accession number in repository **OR** supplier name, catalog number, clone number, **OR** RRID. | Yes | Materials and methods |
| **Animal observed in or captured from the field:** Provide species, sex, and age where possible**.** | Not Applicable | |
| Please detail **housing and husbandry conditions**. | Yes | Materials and methods |

| Plants and microbes | Information included in the manuscript? | In which section is the information available? (Reagents and Tools Table, Materials and Methods, Figures, Data Availability Section) |
|---|---|---|
| **Plants:** provide species and strain, ecotype and cultivar where relevant, unique accession number if available, and source (including location for collected wild specimens). | Not Applicable | |
| **Microbes:** provide species and strain, unique accession number if available, and source. | Not Applicable | |

| Human research participants | Information included in the manuscript? | In which section is the information available? (Reagents and Tools Table, Materials and Methods, Figures, Data Availability Section) |
|---|---|---|
| If collected and within the bounds of privacy constraints report on age, sex and gender or ethnicity for all study participants. | Not Applicable | |

| Core facilities | Information included in the manuscript? | In which section is the information available? (Reagents and Tools Table, Materials and Methods, Figures, Data Availability Section) |
|---|---|---|
| If your work benefited from core facilities, was their service mentioned in the acknowledgments section? | Yes | Acknowledgements |

**Design**

| Study protocol | Information included in the manuscript? | In which section is the information available? (Reagents and Tools Table, Materials and Methods, Figures, Data Availability Section) |
|---|---|---|
| If study protocol has been **pre-registered, provide DOI in the manuscript**. For clinical trials, provide the trial registration number **OR** cite DOI. | Not Applicable | |
| Report the **clinical trial registration number** (at ClinicalTrials.gov or equivalent), where applicable. | Not Applicable | |

| Laboratory protocol | Information included in the manuscript? | In which section is the information available? (Reagents and Tools Table, Materials and Methods, Figures, Data Availability Section) |
|---|---|---|
| Provide DOI OR other citation details if **external detailed step-by-step protocols** are available. | Not Applicable | |

| Experimental study design and statistics | Information included in the manuscript? | In which section is the information available? (Reagents and Tools Table, Materials and Methods, Figures, Data Availability Section) |
|---|---|---|
| Include a statement about **sample size** estimate even if no statistical methods were used. | Not Applicable | |
| Were any steps taken to minimize the effects of subjective bias when allocating animals/samples to treatment (e.g. **randomization procedure**)? If yes, have they been described? | Not Applicable | |
| Include a statement about **blinding** even if no blinding was done. | Not Applicable | |
| Describe **inclusion/exclusion criteria** if samples or animals were excluded from the analysis. Were the criteria pre-established?<br><br>If sample or data points were omitted from analysis, report if this was due to attrition or intentional exclusion and provide justification. | Not Applicable | |
| For every figure, are **statistical tests** justified as appropriate? Do the data meet the assumptions of the tests (e.g., normal distribution)? Describe any methods used to assess it. Is there an estimate of variation within each group of data? Is the variance similar between the groups that are being statistically compared? | Yes | Figure legends |

| Sample definition and in-laboratory replication | Information included in the manuscript? | In which section is the information available? (Reagents and Tools Table, Materials and Methods, Figures, Data Availability Section) |
|---|---|---|
| In the figure legends: state number of times the experiment was **replicated** in laboratory. | Yes | Figure legends |
| In the figure legends: define whether data describe **technical or biological replicates**. | Yes | Figure legends |

## Ethics

| Ethics | Information included in the manuscript? | In which section is the information available? (Reagents and Tools Table, Materials and Methods, Figures, Data Availability Section) |
|---|---|---|
| Studies involving **human participants**: State details of **authority granting ethics approval** (IRB or equivalent committee(s), provide reference number for approval. | Not Applicable | |
| Studies involving **human participants**: Include a statement confirming that **informed consent** was obtained from all subjects and that the experiments conformed to the principles set out in the WMA Declaration of Helsinki and the Department of Health and Human Services Belmont Report. | Not Applicable | |
| Studies involving **human participants:** For publication of **patient photos**, include a statement confirming that consent to publish was obtained. | Not Applicable | |
| Studies involving experimental **animals**: State details of **authority granting ethics approva**l (IRB or equivalent committee(s), provide reference number for approval. Include a statement of compliance with ethical regulations. | Not Applicable | |
| Studies involving **specimen and field samples:** State if relevant **permits** obtained, provide details of authority approving study; if none were required, explain why. | Not Applicable | |

| Dual Use Research of Concern (DURC) | Information included in the manuscript? | In which section is the information available? (Reagents and Tools Table, Materials and Methods, Figures, Data Availability Section) |
|---|---|---|
| Could your study fall under dual use research restrictions? Please check biosecurity documents and list of **select agents and toxins** (CDC): https://www.selectagents.gov/sat/list.htm | Not Applicable | |
| If you used a select agent, is the security level of the lab appropriate and reported in the manuscript? | Not Applicable | |
| If a study is subject to dual use research of concern regulations, is the name of the **authority granting approval and reference number** for the regulatory approval provided in the manuscript? | Not Applicable | |

## Reporting

The MDAR framework recommends adoption of discipline-specific guidelines, established and endorsed through community initiatives. Journals have their own policy about requiring specific guidelines and recommendations to complement MDAR.

| Adherence to community standards | Information included in the manuscript? | In which section is the information available? (Reagents and Tools Table, Materials and Methods, Figures, Data Availability Section) |
|---|---|---|
| State if relevant guidelines or checklists (e.g., **ICMJE, MIBBI, ARRIVE, PRISMA**) have been followed or provided. | Not Applicable | |
| For **tumor marker prognostic studies,** we recommend that you follow the **REMARK** reporting guidelines (see link list at top right). See author guidelines, under 'Reporting Guidelines'. Please confirm you have followed these guidelines. | Not Applicable | |
| For **phase II and III randomized controlled trials**, please refer to the **CONSORT** flow diagram (see link list at top right) and submit the CONSORT checklist (see link list at top right) with your submission. See author guidelines, under 'Reporting Guidelines'. Please confirm you have submitted this list. | Not Applicable | |

## Data Availability

| Data availability | Information included in the manuscript? | In which section is the information available? (Reagents and Tools Table, Materials and Methods, Figures, Data Availability Section) |
|---|---|---|
| Have **primary datasets** been deposited according to the journal's guidelines (see 'Data Deposition' section) and the respective accession numbers provided in the Data Availability Section? | Yes | Data Availability Section |
| Were **human clinical and genomic datasets** deposited in a public access-controlled repository in accordance to ethical obligations to the patients and to the applicable consent agreement? | Not Applicable | |
| Are **computational models** that are central and integral to a study available without restrictions in a machine-readable form? Were the relevant accession numbers or links provided? | Yes | Data Availability Section |
| If publicly available data were reused, provide the respective **data citations in the reference list**. | Yes | Main text & Reference list |
